# Chemical Constituents of *Callistemon subulatus* and Their Anti-Pancreatic Cancer Activity against Human PANC-1 Cell Line

**DOI:** 10.3390/plants11192466

**Published:** 2022-09-21

**Authors:** Juthamart Maneenet, Ahmed M. Tawila, Ashraf M. Omar, Nguyen Duy Phan, Chiharu Ojima, Masahiro Kuroda, Mao Sato, Mio Mizoguchi, Ikue Takahashi, Suresh Awale

**Affiliations:** Natural Drug Discovery Laboratory, Institute of Natural Medicine, University of Toyama, 2630 Sugitani, Toyama 930-0194, Japan

**Keywords:** preferential cytotoxicity, pancreatic cancer, anti-austerity, *Callistemon subulatus*

## Abstract

An n-hexane extract of *Callistemon subulatus* was found to exhibit potent cytotoxicity against PANC-1 human pancreatic cancer cells, preferentially under nutrition starvation conditions, with a PC_50_ value of 6.2 µg/mL. Phytochemical investigation of this bioactive extract resulted in the isolation of fifteen compounds (**1**–**15**), including a new compound, subulatone A (–). The structure of compound **1** was elucidated using HRFABMS and NMR spectroscopic analyses. The isolated compounds were tested for their preferential cytotoxicity against the PANC-1 human pancreatic cancer cell line, using an anti-austerity strategy. Among these, myrtucommulone A (**2**) showed highly potent preferential cytotoxicity, with a PC_50_ value of 0.28 µM. Myrtucommulone A (**2**) was found to alter PANC-1 cell morphology, inhibit cell migration, and downregulate the PI3K/Akt/mTOR and autophagy signaling pathways in nutrient-deprived media, leading to cancer cell death. Therefore, myrtucommulone A (**2**) is a lead compound for anticancer drug development based on an anti-austerity strategy.

## 1. Introduction

Pancreatic cancer is one of the most devastating malignancies, with a 5-year survival rate of less than 5% [1]. It is the fourth leading cause of cancer-related death in Japan for men and the third for women [2]. The annual incidence of pancreatic cancer has continued to increase by 1% since 2000. According to Global Cancer Observatory (GLOBOCAN) 2020, the estimated number of new cases and deaths worldwide was 495,773 and 466,003, respectively. By 2040, pancreatic cancer is expected to spread, and 765,261 people are expected to die from this disease [3]. It is known as the most aggressive human malignant disease among all types of human cancer. Most often, pancreatic cancer is not detected in the early stages and does not show symptoms until it has spread to other organs [4]. If detected at an early stage, immediate surgery is recommended for a greater chance of survival of the patients [5]. Even after surgery, relapse of this disease is observed within a short period. Therefore, adjuvant chemotherapy is given immediately after surgical resection [6]. Chemotherapeutic agents based on gemcitabine (GEM), such as GEM/docetaxel/capecitabine (GTX) [7], GEM/oxaliplatin [8], GEM/capecitabine [9], GEM/erlotinib [10], and GEM/cisplatin [11], have been clinically used to treat pancreatic cancer [12]. However, it does not improve the overall survival of patients with advanced pancreatic cancer. Moreover, gemcitabine-based regimens have shown many adverse effects [13], including aggressive relapse in a short period [14]. Therefore, the search for new candidates with improved therapeutic efficacy against pancreatic cancer is urgently necessary. Clinical angiography of patients with pancreatic cancer shows that pancreatic tumors are generally hypovascular in nature. To cope with limited nutrition supply due to hypovascularity, pancreatic cancer cells within the tumor microenvironment adapt to tolerate nutrition starvation. Pancreatic cancer cells have been shown to survive under the condition of a complete nutrition-deprived medium for more than 72 h, while normal cells usually die within 24 h in the absence of nutrition. Targeting such a phenomenon of tolerance of cancer cells to nutrition deprivation is a very promising anti-austerity approach in pancreatic cancer drug discovery [15]. In this strategy, plant extracts and compounds are screened for their preferential cytotoxicity (PC) against the pancreatic cancer cell line under two different conditions, nutrient-deprived medium (NDM) and normal nutrient-rich condition (DMEM). Compounds that show selective cytotoxicity against pancreatic cancer cells under the nutrient-deprived medium (nutrient starvation condition) without toxicity under normal nutrient conditions are selected as anti-austerity agents [16]. The activity is denoted as a preferential cytotoxicity value (PC_50_), which represents selective cytotoxicity in NDM. Using this strategy, several medicinal plants, such as *Arctium lappa* [15], *Angelica pubescens* [16], *Ancistrocladus likoko* [17], *Uvaria dac* [18] *Bosenbergia pandurata* [19], *Derris scandens* [20], and *Abies spectabliis* [21] have been investigated as a potential source for antipancreatic cancer agents [15,16,17,18,19,20,21,22,23,24,25]. Among these, arctigenin identified from *Arctium lappa* has advanced to an early phase II clinical trial at the National Cancer Center East (Japan), showing a significant survival benefit among advanced pancreatic cancer patients without toxic effects [26].

In our continued study, we recently found that the *n*-hexane extract of *Callistemon subulatus* collected from Egypt showed a highly potent anti-austerity activity with a PC_50_ value of 6.2 µg/mL against the PANC-1 human pancreatic cancer cell line. Therefore, a phytochemical investigation of *C. subulatus* was carried out to discover the active constituents and elucidate their mechanism of action. The work resulted in the isolation of fifteen compounds (**1**–**15**), including a new compound (**1**). In this paper, we describe the structure elucidation of a new compound subulatone A (**1**), and in vitro anticancer activity of the active constituent, myrtucommulone A (**2**), against the PANC-1 human pancreatic cancer cell line.

## 2. Results

### 2.1. Phytochemical Investigation of Callistemon Subulatus

Callistemon subulatus (Myrtaceae), commonly called bottlebrushes, is an ornamental plant praised for its attractive dark crimson spikes [27]. The plant is indigenous to Australia but is cultivated in many parts of the world because of its beauty and medicinal value. It is traditionally used for the treatment of skin infection, cough, diarrhea, and pain and as an antihemorrhoidal agent [27,28]. The plant is rich in eucalyptol-based essential oils with high industrial and economic value owing to its numerous medicinal benefits [29]. Previous studies of this plant species have reported several polyphenols, triterpenoids, and essential oils with antimicrobial, antioxidant, antinociceptive, antidiarrheal, anti-inflammatory, and cytotoxic activities [27,30,31,32,33,34]. However, the potential of this plant as a source of anticancer agents remains largely unknown. In this study, we discovered C. subulatus extract as a potential source of anti-pancreatic cancer agents and carried out a phytochemical investigation.

**Figure 1:**
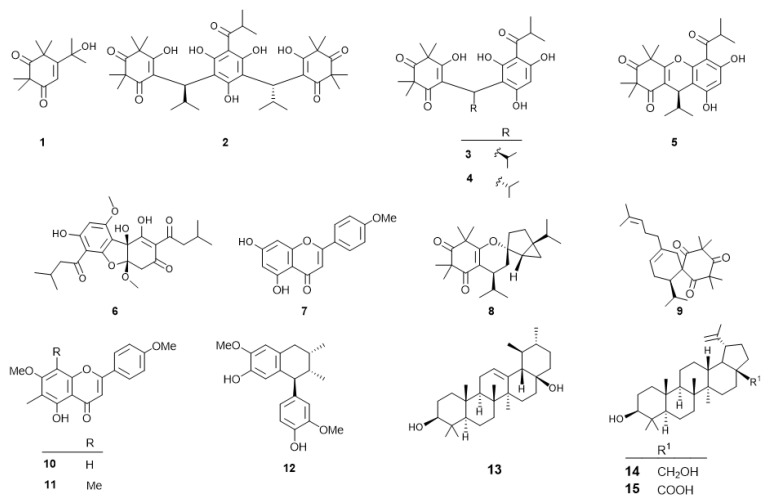
Structures of isolated compounds from *C. subulatus* leaves.

A methanolic extract of *Callistemon subulatus* leaves was suspended in water and partitioned with *n*-hexane. The *n*-hexane fraction was found to be preferentially cytotoxic against the PANC-1 human pancreatic cancer cell line in a nutrient-deprived medium, with a PC_50_ value of 6.2 µg/mL. Phytochemical analysis (see experimental) of the extract led to the isolation of a new compound, subulatone A (**1**), together with 14 known compounds (Figure 1). The known compounds were identified by spectroscopic analysis as (–)-myrtucommulone A (**2**) [35], (–)-myrtucommulone B’ (**3**) [36], (–)-myrtucommulone B’ (**4**) [36], (–)-myrtucommulone B (**5**) [37], callistemenonone A (**6**) [38], acacetin (**7**) [39], callistiviminene F (**8**) [40], calliviminone A (**9**) [40], 8-demethyl eucalyptin (**10**) [41], eucalyptin (**11**) [41], isoguaiacin (**12**) [42], uvaol (**13**) [43], betulin (**14**) [44], and betulinic acid (**15**) [45]. 

**Figure 2:**
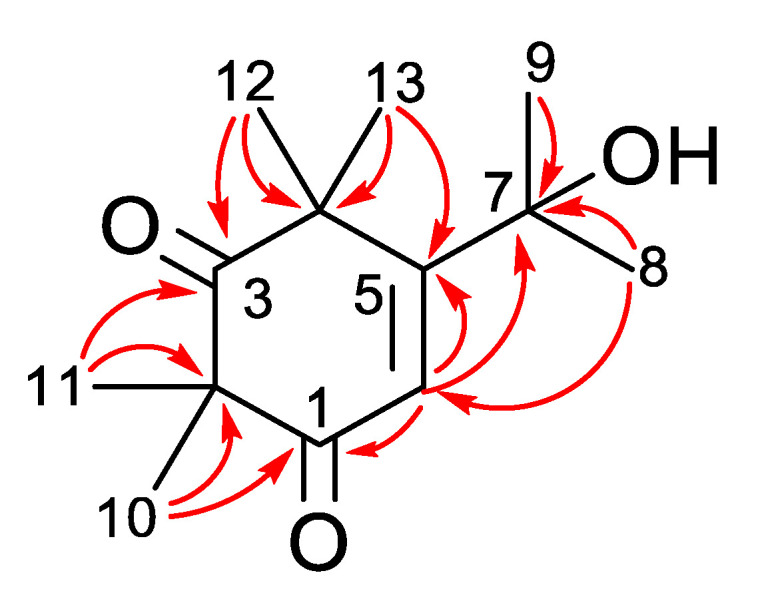
Key HMBC (red arrows) correlations observed in subulatone A (**1**).

Subulatone A (**1**) was obtained as green wax. The molecular formula was deduced from HRFABMS as C_13_H_20_O_3_ [m/z 224.21432 (M)+]. The IR spectrum showed the presence of conjugated carbonyl (1634 cm^−1^) and hydroxyl groups (3479 cm^−1^). The UV spectrum of **1** showed an absorption maxima at 286 nm, indicating the presence of a conjugated carbonyl group. The ^1^H NMR data (Table 1) showed signals of six methyl groups (δ_H_ 1.49, 1.37, 1.37, 1.35, 1.31, 1.03) and an olefinic proton (δ_H_ 7.13). The ^13^C NMR (Table 1) revealed the presence of thirteen carbon signals representing two carbonyls (δ_C_ 210.9, 199.0), two olefinic (δ_C_ 143.1, 97.5), an oxygenated quaternary carbon (δ_C_ 79.5), two quaternary carbons (δ_C_ 55.1, 51.7) and six methyl groups (δ_C_ 26.7, 24.2, 24.0, 23.8, 20.0, and 15.3). Analysis of the Heteronuclear Multiple Quantum Correlation (HMQC) spectra and Heteronuclear Multiple Bond Correlation (HMBC) leads to the assignment of each proton and carbon signal. In the HMBC spectrum, correlations between Me-10 and Me-11 with C-1 and C-3 carbonyl groups suggest the presence of a beta-diketone moiety (Figure 2). Similarly, the HMBC correlation between Me-12 and Me-13 with olefinic carbons C-5 and from H-6 to carbonyl group C-1 and quaternary carbon C-7 established the planar structure of subulatone A (**1**) as 5-(2-hydroxypropan-2-yl)-2,2,4,4-tetramethylcyclohex-5-ene-1,3-dione.

### 2.2. Preferential Cytotoxicity against PANC-1 Cells 

All isolated compounds were investigated for their anti-austerity activity against PANC-1 human pancreatic cancer cells. The preferential cytotoxic activity (PC_50_) represents the concentration at which 50% of the cancer cell death selectively in a nutrient-deprived medium (NDM) (Table 2). Gemcitabine, a clinically used anticancer agent, was used as a standard reference in this study [24]. Arctigenin, a well-known anti-austerity agent, was used as a positive control [15,46].

**Figure 3:**
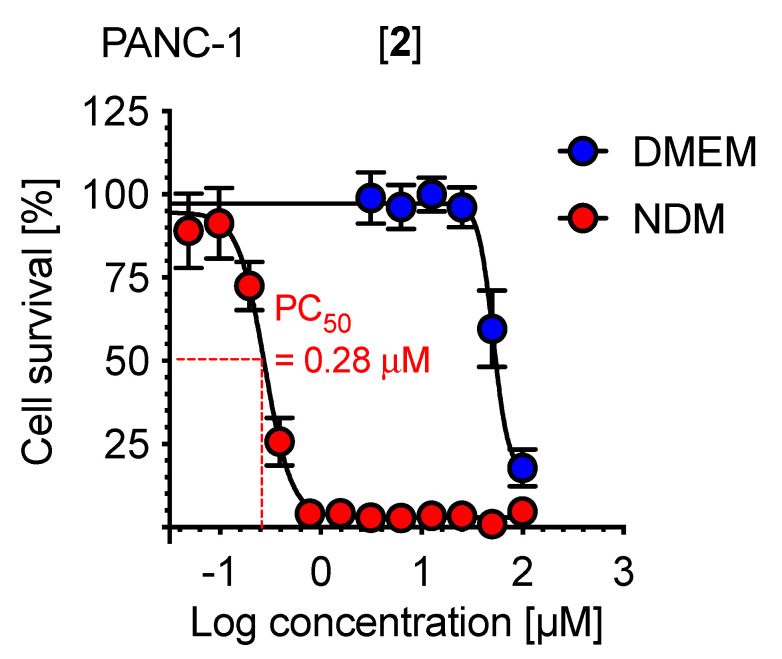
Preferential cytotoxicity activity of myrtucommulone A (**2**) against the PANC-1 human pancreatic cancer cell line in nutrient-deprived medium (NDM) and Dulbecco’s modified Eagle’s medium (DMEM).

The isolated compounds can be categorized as meroterpenoids (**1–6**, **8**, and **9**), flavonoids (**7**, **10**, and **11**), lignan (**12**), and triterpenes (**13–15**). The activity of the meroterpenoids ranged from 0.02 µM to 42.2 µM (Table 2). Although a clear structure and activity could not be deduced in the present study, callistrilone-type meroterpenoids have been found to be the most potent anti-austerity agents [23,47]. Among the flavonoids, a methyl unit at the C-8 position was found to enhance activity (**11** > **10**, **7**). Among the oleanan- and lupan-type triterpenes, the presence of an alcohol group at C-28 leads to a dramatic enhancement of activity compared to the carboxylic acid group (**14** >> **13**, **15**). Among the potent compounds, a callistrilone-type meroterpenoid, myrtucommulone A (**2**) having PC_50_ value of 0.28 µM (Figure 3), was isolated in large amounts (13.7 mg). Therefore, we performed a detailed biological study of compound **2** against the PANC-1 human pancreatic cancer cell line. 

### 2.3. Morphological Change in PANC-1 Cells in NDM

**Figure 4:**
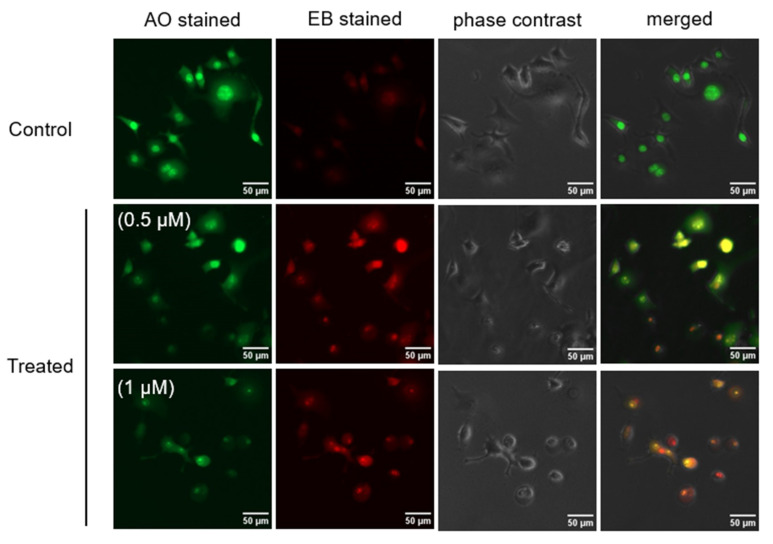
Morphology changes of PANC-1 cells induced by myrtucommulone A (**2**) at concentrations of 0.5 and 1 µM, which incubated for 24 h in nutrient-deprived medium (NDM) compared with control PANC-1 cells.

To investigate changes in the morphology of PANC-1 cells induced by myrtucommulone A (**2**), a fluorescence-microscopy-based ethidium bromide-acridine orange (EB-AO) double staining assay was used. In this assay, cells emitting green fluorescence indicate the live cells, and the cells with red or orange fluorescence indicate the dead or dying cells. As shown in Figure 4, untreated (control) PANC-1 cells emitted green fluorescence, indicating cell survival in the NDM medium. On the other hand, cells treated with myrtucommulone A (**2**) at the concentration of 0.5 µM and 1 µM showed a concentration-dependent increase in the population of cells emitting orange or red fluorescence with condensed nuclei and altered cell membranes.

**Figure 5:**
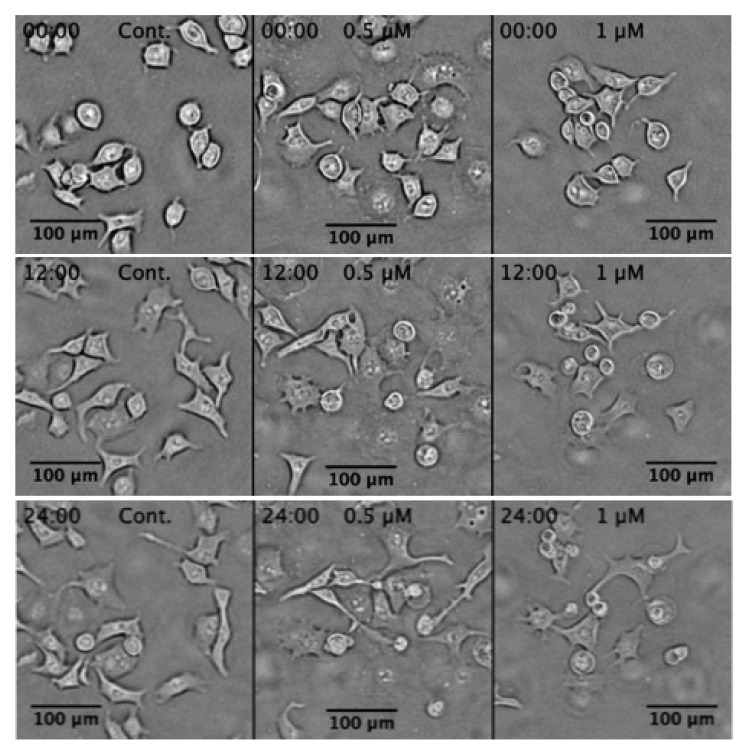
Morphology changes of PANC-1 cells induced by myrtucommulone A (**2**) at concentrations of 0.5 and 1 µM, incubated for 24 h in nutrient-deprived medium (NDM) compared with control PANC-1 cells. (See Supplementary Video S1).

To further investigate the real-time effect of compound **2**, a live time-lapse imaging study was performed on PANC-1 cells. As shown in Figure 5, the untreated PANC-1 cells showed an intact morphology and survived for 24 h. In contrast, treatment of PANC-1 cells with **2** at 0.5 and 1 µM led to cell shrinkage and membrane blebbing. Details of the cell death process induced by **2** in real-time are given in Supplementary Video S1.

### 2.4. Inhibition of PANC-1 Cell Migration in DMEM

**Figure 6:**
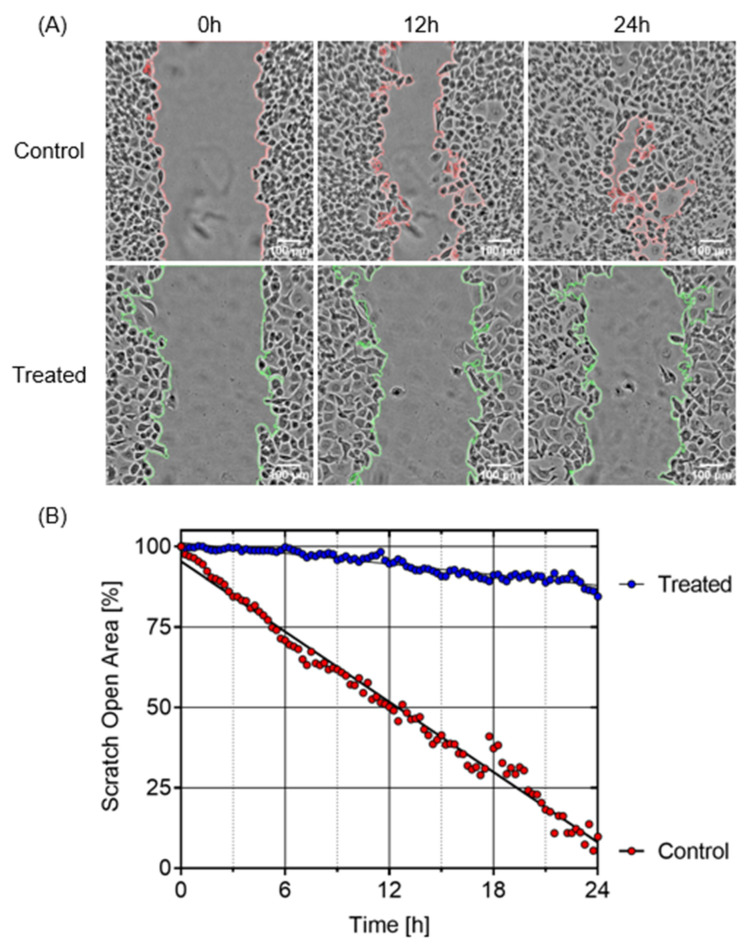
The effect of myrtucommulone A (**2**) on inhibition of the migration of PANC-1 cells in a real-time showing image of the open area at 0 h, 12 h, and 24 h (**A**); and quantification of the migration of PANC-1 cells by determining the percentage of open area at an interval of 15 min for 24 h (**B**) (see Supplementary Video S2).

Pancreatic cancer cells generally migrate from the nutrient-deficient tumor microenvironment to nutrient-rich organs such as the liver and stomach. Therefore, to evaluate the inhibition potential of myrtucommulone A (**2**) against PANC-1 cell migration in normal nutrient-rich conditions, the assay was performed in DMEM. For this purpose, PANC-1 cells were seeded in a ibidi two-well culture insert µ-dish to create a symmetric open area. The cells were then treated with myrtucommulone A (**2**) at 0.5 and 1 µM, or untreated control, and were allowed to migrate for 24 h within the CO_2_ incubator. Real-time images were captured at an interval of 15 min for 24 h. The open area at each time point was quantified using ImageJ software. As shown in Figure 6, the untreated control cells migrated to the open area very fast and closed in 24 h. In contrast, treatment with myrtucommulone A (**2**) significantly inhibited cell migration, with an open area of 84.5% compared to T_0_ (see Supplementary Video S2).

### 2.5. Inhibition of PI3K/Akt/mTOR and Autophagy Signaling Pathway

**Figure 7:**
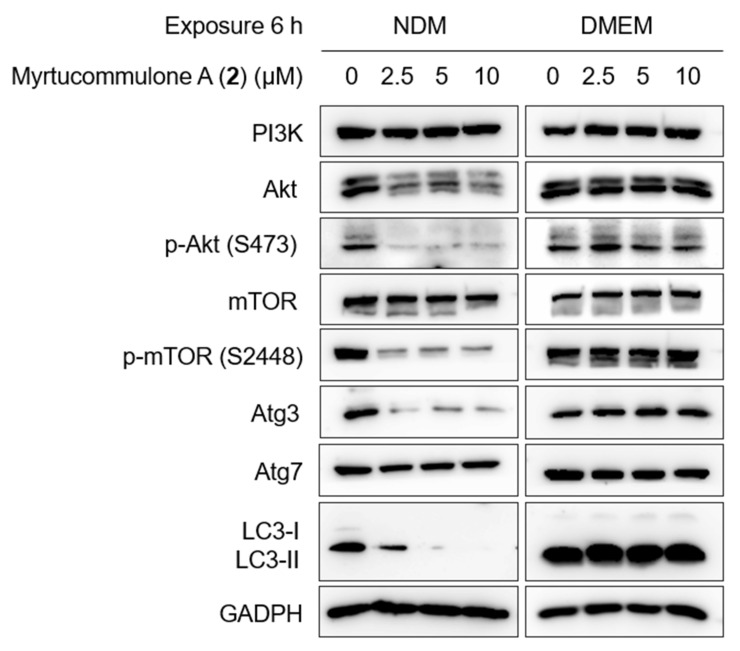
Effect of myrtucommulone A (**2**) on protein related to the PI3K/Akt/mTOR and autophagy signaling pathway under NDM and DMEM.

**Figure 8:**
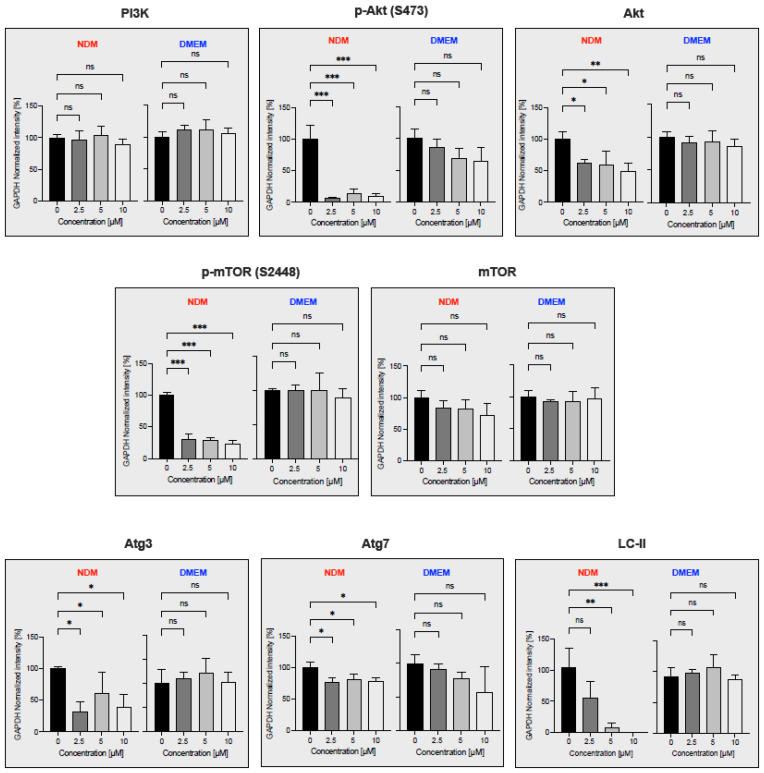
The percentage of GAPDH normalized intensity of myrtucommulone A (**2**) on protein related to the PI3K/Akt/mTOR and autophagy signaling pathway under NDM and DMEM. The statistical significance was calculated using one-way ANOVA of three independent experimental results using GraphPad Prism 9. * *p* < 0.05; ** *p* < 0.01; *** *p* < 0.001 indicates a significant difference from the control.

PI3K/Akt/mTOR and autophagy Signaling Pathway is frequently activated in pancreatic cancer and confers the tolerance to nutrition starvation within tumor microenvironment {reference}. Therefore, to determine whether myrtucommulone A (**2**) modulates the proteins involved in PI3K/Akt/mTOR and autophagy signaling pathways, Western blot analysis was performed. For this purpose, the PANC-1 cells were treated with myrtucommulone A (**2**) for a brief period of 6 h at concentrations of 2.5 µM, 5 µM, and 10 µM in two different mediums, nutrient-deprived medium (NDM) and normal nutrient-rich medium (DMEM). As shown in Figure 7 and Figure 8, there were no significant changes in the expression of each protein in DMEM. On the contrary, most protein components of these pathways have been significantly down-regulated by myrtucommulone A in a concentration-dependent manner under the tumor microenvironment mimicking the condition of nutrient deprivation. The inhibition of Akt and mTOR phosphorylation was 93.3% and 70%, respectively, at a concentration of 2.5 µM (Figure 8). Under nutrition deprived condition, nonessential and damaged cell organelles are recycled through lysosomal-mediated degradation to maintain the ATP within tumor cells and promote cancer cell survival. This process, also known as autophagy, is regulated by several autophagy-related (Atg) proteins and microtubule-associated protein light chain 3 (LC3). Remarkably, treatment with myrtucommulone A significantly inhibited key autophagy regulatory proteins Atg3 and LC3-II in NDM (Figure 7 and Figure 8) in NDM and, in turn, contributed to PANC-1 cell death.

## 3. Discussion

Pancreatic cancer is one of the deadliest forms of cancer, with the lowest five-year survival rate. The mortality due to pancreatic cancer is increasing dramatically throughout the world [4]. Currently, various chemotherapeutic agents are used clinically for the treatment of advanced pancreatic cancer, but none of these agents are found to be effective in treating pancreatic cancer. It has the lowest 5-year overall survival among all known human cancer types, with median survival only 6 months after diagnosis. The failure of conventional chemotherapeutic agents in clinical use is partly attributed to the hypovascular nature of the pancreatic tumor and adaptation of cancer cells to nutrition starvation condition within the tumor microenvironment [48,49]. In earlier studies, PANC-1 cancer cells have been shown to survive for more than 3 days, even in the complete absence of essential nutrients such as glucose, amino acids, and serum. This survival capacity of cancer cells under conditions of nutrient starvation is commonly called ‘austerity’ [25,50]. The search for a candidate that eliminates the cancer cells’ tolerance to nutrition starvation is a unique approach to anticancer drug discovery. Based on this approach, an anti-austerity screening strategy has been developed to find anti-pancreatic cancer agents from medicinal plants of diverse origins [15,16,17,18,19,20,21,22,23,24,25]. In a continued effort, recently, we observed highly potent anti-austerity activity in the *n*-hexane extract of *Callistemon subulatus* at the sub micromolar level, the strongest amongst plant extracts ever screened in the past. Therefore, to discover potential anticancer agents from this extract, a phytochemical investigation was carried out and isolated fifteen compounds (**1**–**15**). Anti-austerity activity evaluation of the isolated compound revealed that myrtucommulone A (**2**) was a major active compound with a PC_50_ value of 0.28 µM. Therefore, it was subjected to an in-depth study. At first, the effect of myrtucommulone A (**2**) on the changes in PANC-1 cell morphology and apoptosis in NDM was investigated using an ethidium bromide-acridine orange (EB-AO) double staining assay. As shown in Figure 4, the control cells showed intact cell morphology and emitted exclusive green fluorescence of live cells due to AO. On the other hand, cells treated with **2** resulted in rounding of cell membranes leading to cell death in a concentration-dependent manner and emitted orange or red fluorescence due to EB staining. Furthermore, a live imaging study showed that treatment with myrtucommulone A (**2**) lead to cell shrinkage and membrane blebbing in PANC-1 cells within 12 h, leading to total cell death within 24 h (Figure 5, Supporting Video S1). Therefore, a subsequent investigation on myrtucommulone A (**2**) was carried out for its antimetastatic potential.

Most pancreatic cancer patients are diagnosed at an advanced metastatic stage when cancer has already spread to other organs such as the liver, lung, or peritoneal cavity [21,51]. To evaluate the antimetastatic potential of myrtucommulone A (**2**), a cell migration assay was performed. As shown in Figure 6 and see Supplementary Video S2, myrtucommulone A (**2**) potently inhibited PANC-1 cell migration in real time with 85% of open area compared to control with total closure of the open area within 24 h. It should be noted that most of the published studies on cell migration in the literature reported only end point data, which are less reliable and even lead to a false interpretation of the data. Live imaging performed in this study provides an unbiased and accurate interpretation of the antimetastatic effect of the myrtucommulone A (**2**). 

The PI3K/Akt/mTOR signaling pathway is the most often activated in many cancer types, including pancreatic cancer, and regulates cancer cell survival, proliferation, metabolism, metastasis, and cancer invasion [52,53,54]. Inhibitors of this pathway are the potential target in cancer therapy. Several FDA-approved small-molecule inhibitors such as idelalisib [55], copanlisib [56], and duvelisib [57] targeting the PI3K/Akt/mTOR pathway are currently in clinical development at various stages for the treatment of lymphoma. The Akt inhibitor MK2206, in combination with dinaciclib, has shown a favorable response in advanced pancreatic cancer patients in phase II clinical trials in patients with advanced, unresectable/metastatic pancreatic cancer [58]. Therefore, inhibition of Akt/mTOR signaling components by myrtucommulone A (**2**) further suggests its potential for drug development against pancreatic cancer. Another major process by which cancer cells tend to survive during nutrition deprivation is the activation of autophagy, a process of recycling unwanted cellular debris and maintaining energy balance under nutrition deprivation within the tumor microenvironment [59,60]. Autophagy is regulated by the Atg proteins and the microtubule-associated protein light chain 3 (LC3) [61,62]. Inhibition of autophagy components under nutrition starvation will cause energy catastrophe within the tumor microenvironment, leading to cancer cell death. Remarkably, autophagy regulatory proteins Atg3 and LC3 were significantly downregulated by myrtucommulone A (**2**) in a concentration-dependent manner (Figure 7 and Figure 8). This evidence suggests that myrtucommulone A (**2**) and related compounds are promising agents for the development of anticancer drugs based on the anti-austerity strategy.

## 4. Materials and Methods

### 4.1. General Experimental Procedure

IR spectra were measured with a JASCO FT/IR-460 Plus spectrophotometer. One-dimensional and two-dimensional NMR spectra were recorded in the JEOL ECA400II Delta spectrometer, using chloroform-*d* as solvent and TMS as internal standard, and chemical shifts are expressed in δ values. HRFABMS measurements were carried out on a JEOL JMS-AX505HAD mass spectrometer, and glycerol was used as a matrix. Medium-pressure liquid chromatography (MPLC) was performed with a Büchi MPLC C-605 double gradient pump system with normal-phase silica gel (silica gel 60N, Kanto Chemical, Japan). Analytical TLC was carried out on pre-coated silica gel 60F254 and RP-18F254 plates (0.25 or 0.50 mm, Merck KGaA, Darmstadt, Germany). Preparative HPLC separations were performed on an Agilent 1260 infinity quaternary LC VL instrument with a TSKgel ODS-100V column (250 × 30 mm i.d.; 5 μm, Tosoh).

### 4.2. Chemical and Antibodies

Fetal bovine serum (FBS) was purchased from Nichirei Biosciences Inc. (Tokyo, Japan). Antibiotic/antimycotic solutions were purchased from Sigma-Aldrich. Dulbecco’s modified Eagle’s medium (DMDM) was purchased from Wako Pure Chemical (Osaka, Japan). Nutrient-deprived medium was prepared according to a protocol described previously [15]. HEPES and the cell counting kit-8 were purchased from Djindo Laboratories (Kumamoto, Japan). Rabbit polyclonal antibodies against PI3K, Akt, phosphoryl Akt (S473), mTOR, phosphoryl mTOR (S2448), Atg3, Atg7, LC3, and GAPDH were purchased from Cell Signaling Technology (Danvers, MA, USA). Horseradish peroxidase-conjugated goat polyclonal anti-rabbit was purchased from DakoCytomation (Glostrup, Denmark).

### 4.3. Extraction and Isolation

The dried leaves of *C. subulatus* (1.6 kg) were collected from El-Zohreya Botanical Garden, Giza, Egypt, in April 2019. The plant was authenticated by senior botanist Mrs. Therese Labib, Consultant in Plant Taxonomy at the Ministry of Agriculture and El-Orman Botanical Garden, Giza, Egypt. A voucher specimen (000094CC@06-01-04-18) was deposited in the Herbarium of the El-Orman Garden. The plant material was dried, ground, and kept in a tight container. The plants were macerated with methanol by sonication (5 L, 90 min ×3) at room temperature. The solvent was evaporated to obtain the MeOH extract (417.0 g). The extract was suspended in water and partitioned with *n*-hexane to obtain a fraction of *n*-hexane (53.4 g). This fraction was chromatographed on silica gel by MPLC (Buchi MPLC, C-601/C-605 dual pump) using an *n*-hexane/EtOAc solvent system gradient mixture (0–100) to obtain seven fractions (Fr. 1, 23.1 g; Fr. 2, 5.1 g; Fr. 3, 8.8 g; Fr. 4, 3.1 g; Fr. 5, 2.9 g; Fr. 6, 1.7 g; Fr. 7, 3.0 g). Fraction 1 (23.1 g) was chromatographed on normal-phase silica gel MPLC using *n*-hexane/CH_2_Cl_2_ solvent mixtures with increasing ratios of CH_2_Cl_2_ to obtain five subfractions (Fr. 1-1, 3.0 g; Fr. 1-2, 1.2 g; Fr. 1-3, 4.2 g; Fr. 1-4, 5.6 g; Fr. 1-5, 8.5 g). Subfractions 1-2 (1.2 g) were purified by passage over a Sephadex LH-20 column using MeOH/CH_2_Cl_2_ to obtain seven fractions. Subfractions 1-2-6 (123 mg) were subjected to preparative HPLC on an ODS column (30 × 250 mm, 5 μm, Tosoh) using an eluent system consisting of MeOH containing 0.03% formic acid to obtain myrtucommulone A (2, 13.7 mg), calliviminone A (9, 1.1 mg), and callistiviminene F (8, 3.0 mg). Fraction 3 (8.8 g) was rechromatographed by normal-phase silica gel MPLC using *n*-hexane/CH_2_Cl_2_/0.1% MeOH as solvent system with a gradual increase in CH_2_Cl_2_ to afford five subfractions (Fr. 3-1, 1.3 mg; Fr. 3-2, 1.7 g; Fr. 3-3, 723 mg; Fr. 3-4, 2.5 g; Fr. 3-5, 1.5 mg). Subfractions 3-2 (1.7 g) were purified via normal-phase silica gel MPLC using a gradient solvent mixture of *n*-hexane/EtOAc to obtain subulatone A (1, 5.2 mg), 8-demethyl eucalyptin (10, 5.2 mg), eucalyptin (11, 7.6 mg), and isoguaiacin (12, 2.3 mg). Subfractions 3-4 (2.5 g) were purified by normal phase silica gel MPLC using a gradient solvent mixture of *n*-hexane/EtOAc to give acacetin (7, 36.3 mg), uvaol (13, 4.1 mg), betulin (14, 16.0 mg), and betulinic acid (15, 72.3 mg). Fraction 6 (1.7 g) was re-chromatographed over normal-phase silica gel MPLC using *n*-hexane/CH_2_Cl_2_/0.1% MeOH as solvent system with a gradual increase in CH_2_Cl_2_, to afford four subfractions (Fr. 6-1, 112 mg; Fr.6-2, 123 mg; Fr.6-3, 315 mg; Fr. 6-4, 987 mg). Subfractions 6-2 (123 mg) were subjected to preparative HPLC on an ODS column (30 × 250 mm, 5 μm, Tosoh) using an eluent system consisting of 90% MeOH/H_2_O to obtain (–)-myrtucommulone B’ (3, 29.5 mg), (+)-myrtucommulone B’ (4, 13.8 mg), (–)-myrtucommulone B (5, 9.6 mg), and callistemenonone A (6, 1.3 mg).

*Subulatone A* (**1**): green wax; UV (MeOH) λ_max_ (log ε) 286 (4.21) nm; IR (KBr) ν_max_ 3479, 1654 cm^−1^; HRFABMS *m/z* 224.21432 [M]^+^ (calcd for C_13_H_20_O_3_); ^1^H and ^13^C NMR (400 MHz, chloroform-d: see Table 1. (See Supplementary Material for the original spectroscopic data).

### 4.4. Cell Line and Cell Culture of PANC-1

The human pancreatic cancer cell line PANC-1 (RBRC-RCB2095) was purchased from the Riken BRC cell bank. Cells were maintained in standard DMEM supplemented with 10% FBS, 0.1% sodium bicarbonate, and a 1% antibiotic antimycotic solution [15].

### 4.5. Preferential Cytotoxicity Assay against PANC-1 Cells

The preferential cytotoxicity assay was performed according to the previously described procedure using the Cell counting kit-8 [15]. Briefly, PANC-1 cancer cells (2 × 10^4^/well) were seeded in 96-well plates and incubated at 37 °C under 5% CO_2_ for 24 h. The cells were then washed with PBS and treated with serially diluted test samples in DMEM and NDM with control and blank on each plate. After 24 h incubation, the media were replaced with 100 μL of DMEM containing 10% WST-8 cell counting kit solution in each well and further incubated for 3 h. Finally, the absorbance at 450 nm was measured (Perkin-Elmer EnSpire multilabel reader). Cell viability was calculated from the mean values of data from three wells by using the following equation:

Cell viability [%] = [{Abs_(test sample)_/Abs_(control)_} − Abs_(blank)_] × 100

### 4.6. Morphological Assessment of PANC-1 Cells

The morphological experiments were determined by ethidium bromide-acridine orange (EB/AO) double staining assay [24]. For this purpose, 2.0 × 10^5^ PANC-1 cells were seeded in 35 mm dishes and incubated for 24 h for cell attachment. The cells were then treated with myrtucommulone A (2) at concentrations of 0.5 and 1 µM in NDM. The control and treated cells were then placed inside a CO_2_ incubator for 24 h. At the end of the experiment, 10 µL of EB-AO double staining reagent was added to each dish and incubated in the dark for 10 min. The images were then captured using an EVOS-FL digital imaging system in the phase-contrast and fluorescence modes.

### 4.7. Live Cell imaging of PANC-1 Cells

To further investigate the real-time effect of myrtucommulone A (2) on PANC-1 cell death, a live cell imaging system was performed. PANC-1 cell lines (2.0 × 10^5^ cells) were seeded in 35 mm dishes and incubated for 24 h for cell attachment. The cells were then washed with PBS and replaced with NDM containing myrtucommulone A at 0.5 and 1 µM (treated) or NDM only (control). All these dishes of control and treated cells were then placed immediately over CytoSmart real-time microscopy system, and live imaging was performed in parallel. Images were captured every 15 min for 24 h.

### 4.8. Cell Migration Assay

The cell migration assay was performed as previously described [20]. Briefly, PANC-1 cells (1.0 × 10^6^) were seeded in the ibidi culture insert two-well µ-dish (35 mm) and incubated overnight under 5% CO_2_, 37 °C. After the cell attachment, the culture insert was removed, which created an open area of 500 µm cell-free gap. The cells were then treated with myrtucommulone A (2, 20 µM) for the treated group and without the test compound in DMEM for the control. Real-time images were captured at 15 min intervals for 24 h using the CytoSMART digital microscopy system. Finally, the percentage of the open area at each time point was determined using a Fiji (ImageJ) platform.

### 4.9. Western Blot Analysis

Proteins were separated by electrophoresis on a 0.1% SDS-containing polyacrylamide gel and transferred to nitrocellulose membranes. The membranes were blocked with TBST containing 5% (*w*/*v*) skim milk, washed with TBST containing 0.3% Tween 20 (Sigma, St. Louis, France), and incubated overnight at room temperature with primary antibody [PI3K, Akt, phosphoryl Akt (S473), mTOR, phosphoryl mTOR (S2448), LC3, and GAPDH; Cell Signaling Technology, Danvers, MA, USA]. The membranes were washed with TBST and incubated with horseradish peroxidase-conjugated goat anti-rabbit IgG secondary antibody (Santa Cruz Biotechnology, Santa Cruz, CA, USA). The bands were detected using an enhanced chemiluminescence system (Amersham Biosciences UK Ltd., Buckinghamshire, UK).

## 5. Conclusions

Phytochemical investigation of the *n-hexane* extract of *Callistemon subulatus* leaves led to the isolation of 15 compounds, including a new compound named subulatone A (**1**). Among the isolated compounds, myrtucommulone A (**2**) was identified as the most potent compound having selective cytotoxicity against PANC-1 human pancreatic cancer cells with a PC_50_ value of 0.28 µM. Myrtucommulone A (**2**) strongly inhibited PANC-1 cell migration under normal nutrient-rich conditions, suggesting its antimetastatic potential. Furthermore, Myrtucommulone A (**2**) was found to significantly down-regulate Akt/mTOR/autophagy signaling pathway proteins in tumor microenvironment mimicking the condition of nutrient deprivation. Therefore, myrtucommulone A (**2**) is a promising lead compound for developing an antipancreatic cancer drug based on an anti-austerity strategy. The present study warrants further investigation on myrtucommulone A (**2**) as a monotherapy or in combination with conventional anticancer agents against in vivo pancreatic cancer models. It is expected that combination therapy, including myrtucommulone A (**2**), will be useful in overcoming the chemoresistance in pancreatic cancer. Further effort in this area is in due progress.

## Figures and Tables

**Table 1:** ^1^H and ^13^C NMR spectroscopic data for subulatone A (**1**) in CDCl_3_ (δ in ppm, J in Hz).

No.	Subulatone A (1)	
	*δ_C_*	*δ_H_*
1	199.0 *^a^*	-
2	55.1	-
3	210.9 *^a^*	-
4	51.7	-
5	97.5	-
6	143.1	7.13, s
7	79.5	-
8	24.0	1.49, s
9	24.2	1.35, s
10	20.0	1.03, s
11	15.3	1.31, s
12	26.7	1.37, s
13	23.8	1.37, s

*^a^* deduced from HMBC.

**Table 2:** Preferential cytotoxicity (PC_50_) of compounds **1**–**15** against the PANC-1 human pancreatic cancer cell line in nutrient-deprived medium (NDM).

Compound	PC_50_, µM	Compound	PC_50_, µM
1	16.5	9	0.02
2	0.28	10	6.1
3	4.4	11	1.0
4	6.0	12	88.3
5	9.2	13	5.2
6	42.8	14	0.7
7	7.8	15	15.2
8	10.0	Gemcitabine *^a^*	>100
		Arctigenin *^b^*	0.7

*^a^* Standard reference; *^b^* positive control.

## Data Availability

Data used in the analysis are available in the Supplementary Materials.

## Supplementary Materials

The following supporting information can be downloaded at: https://www.mdpi.com/article/10.3390/plants11192466/s1, Figure S1: ^1^H NMR spectrum of subulatone A (**1**); Figure S2: 13C NMR spectrum of subulatone A (**1**); Figure S3: HMQC spectrum of subulatone A (**1**); Figure S4: HMBC spectrum of subulatone A (**1**); Figure S5: HRFABMS spectrum of subulatone A (**1**); Figure S6: IR spectrum of subulatone A (**1**); Figure S7: UV spectrum of subulatone A (**1**); Figure S8: The effect of myrtucommulone A (**2**) on protein related to the PI3K/Akt/mTOR and autophagy signaling pathway under NDM and DMEM of three independent experiments. Video S1: Morphology changes of PANC-1 cells induced by myrtucommulone A (**2**); Video S2: Effect of myrtucommulone A (**2**) on inhibition of PANC-1 cell migration in real-time.
