# Supplementary material for: Chemical Constituents of Callistemon subulatus and Their Anti-Pancreatic Cancer Activity against Human PANC-1 Cell Line
Source: Plants (Basel). 2022 Sep 21;11(19):2466. doi: 10.3390/plants11192466 (PMC9570665; doi:10.3390/plants11192466)
Supplement: Supplementary file 1 [file plants-11-02466-s001.zip › Supplementary information-I.pdf]

Supplementary Materials

Chemical Constituents of *Callistemon subulatus* and Their Anti-pancreatic Cancer Activity Against Human PANC-1 Cell Line

Juthamart Maneenet, Ahmed M. Tawila, Ashraf M. Omar, Nguyen Duy Phan, Chiharu Ojima, Masahiro Kuroda, Mao Sato, Mio Mizoguchi, Ikue Takahashi and Suresh Awale *

Natural Drug Discovery Laboratory, Institute of Natural Medicine, University of Toyama, 2630 Sugitani, Toyama 930-0194, Japan.
* **Correspondence:** suresh@inm.u-toyama.ac.jp; Tel: +81-76-434-7640. Fax: +81-76-434-7640.

Table of Contents

Figure S1. ^{1}H NMR spectrum of subulatone A (**1**) …………………………………… 3

Figure S2. ^{13}C NMR spectrum of subulatone A (**1**) …………………………………… 3

Figure S3. HMQC spectrum of subulatone A (**1**) ……………………………………... 4

Figure S4. HMBC spectrum of subulatone A (**1**) ……………………………………… 5

Figure S5. HRFABMS spectrum of subulatone A (**1**) ……………………………….... 6

Figure S6. IR spectrum of subulatone A (**1**) ……………………………..................... 7

Figure S7. UV spectrum of subulatone A (**1**) ……………………………................... 8

Figure S8. Three independent experiments on the effect of myrtucommulone A (**2**) on proteins related to the PI3K/Akt/mTOR and autophagy signaling pathways in NDM and DMEM. ………………………..…………………..…………………...…….. 9

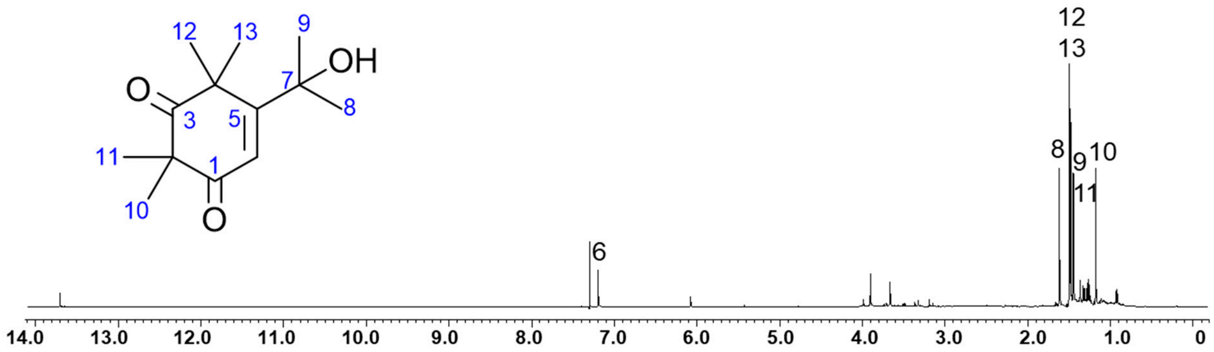

Figure S1. ^{1}H NMR spectrum of subulatone A (**1**).

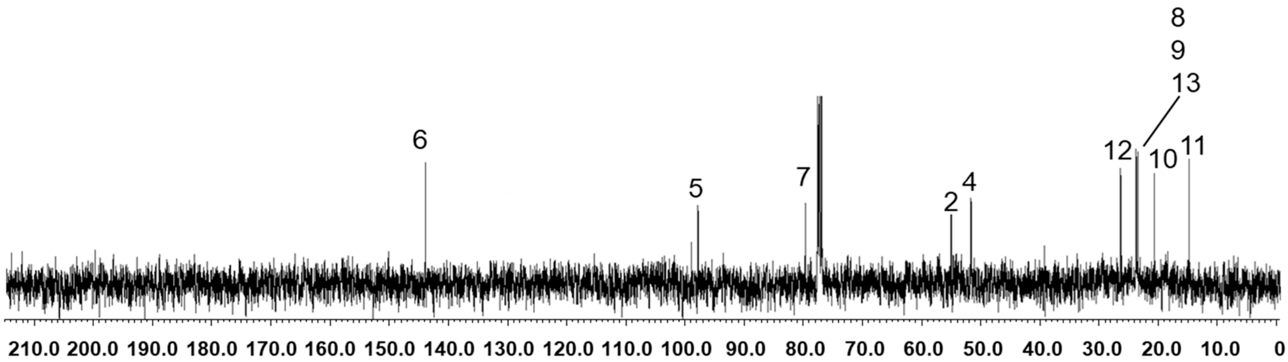

Figure S2. ^{13}C NMR spectrum of subulatone A (**1**).

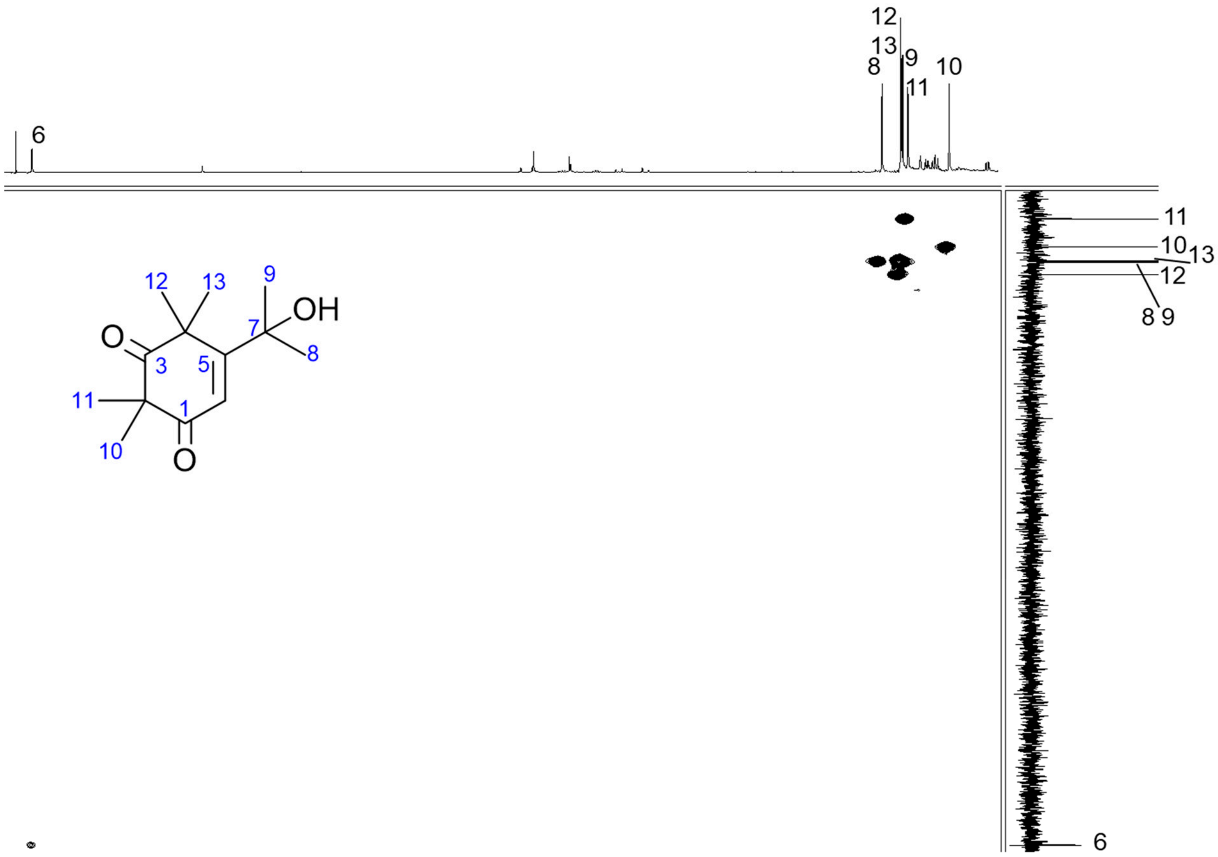

Figure S3. HMQC spectrum of subulatone A (**1**).

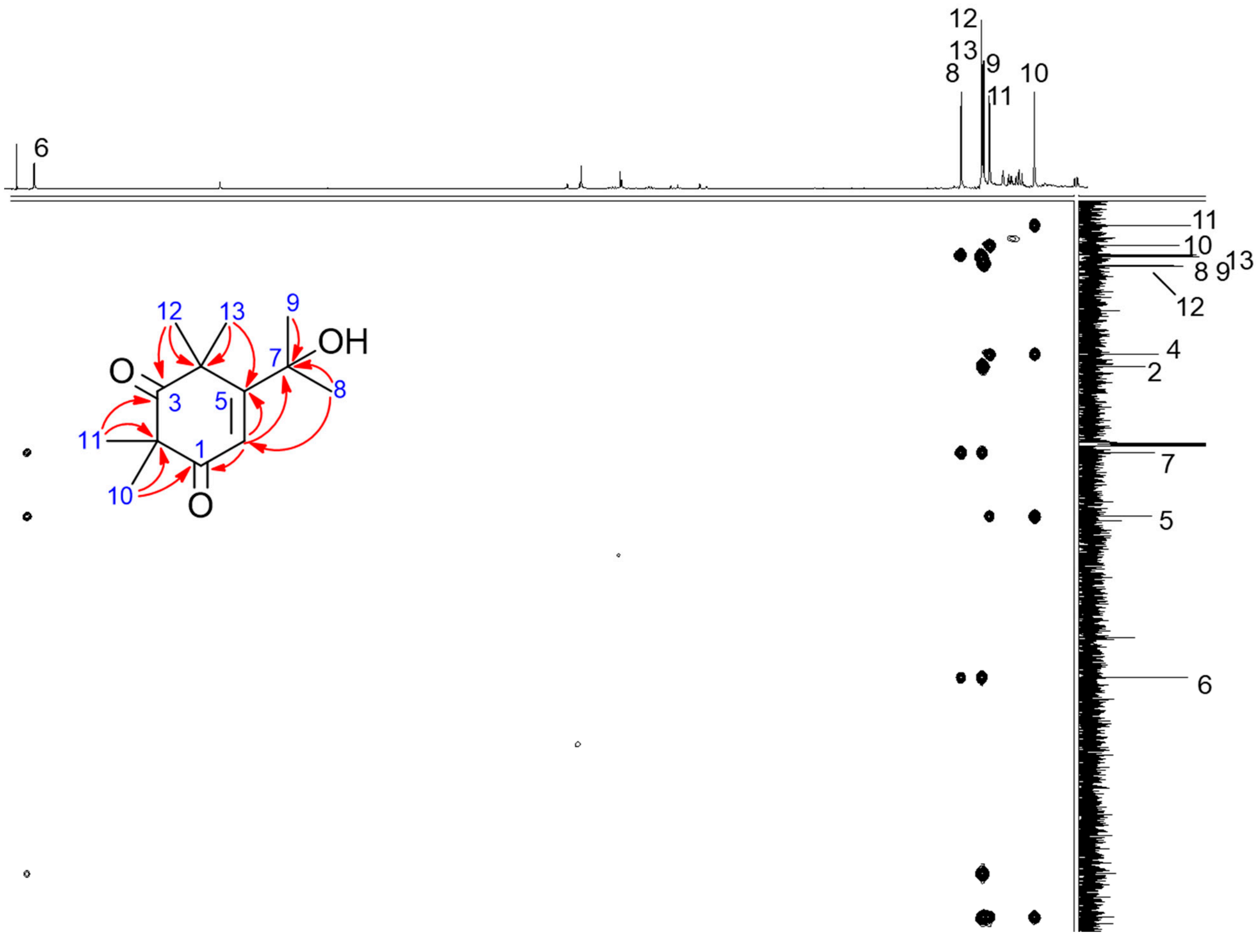

Figure S4. HMBC spectrum of subulatone A (**1**).

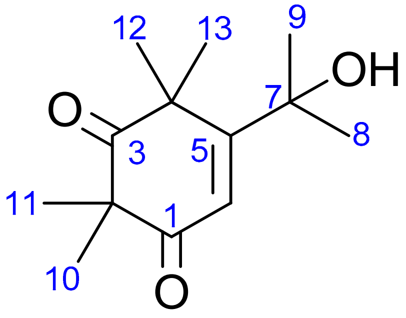

File: Cs-4-4002
Sample: - -
Instrument: AX505W
Inlet: Direct

Date Run: 6-17-2020 (Time Run: 12:35:25)

Ionization mode: El+

R.T.: 3.1

Scan: 65
Base: m/z224; 28%FS TIC: 13825

#Ions: 8

Selected Isotopes : H $C_{0.13}0_{03}$

Error Limit : 20 mmu

Unsaturation Limits : 0 to 50

Measured Mass	*% Base*	*Formula*	*Calculated Mass*	*Error*	*Unsaturation*
22421432	1000%	$C_{13}H_{20}0_3$	224.14000	-03	4.0

Figure S5. HRFABMS spectrum of subulatone A (**1**).

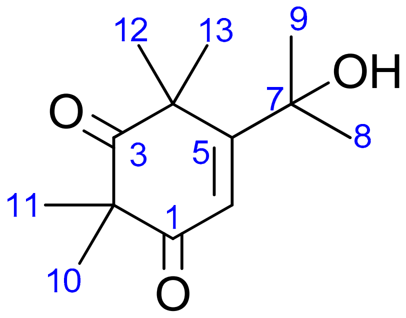

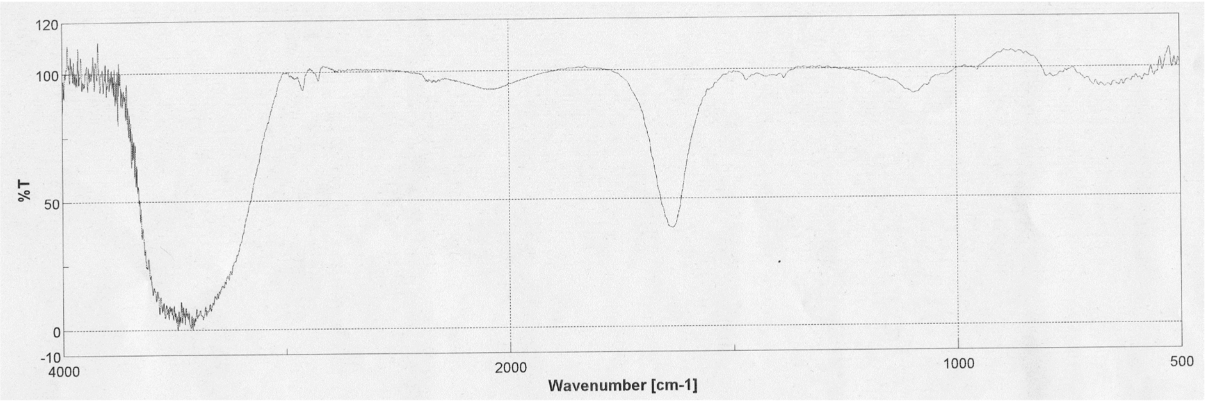

Figure S6. IR spectrum of subulatone A (**1**).

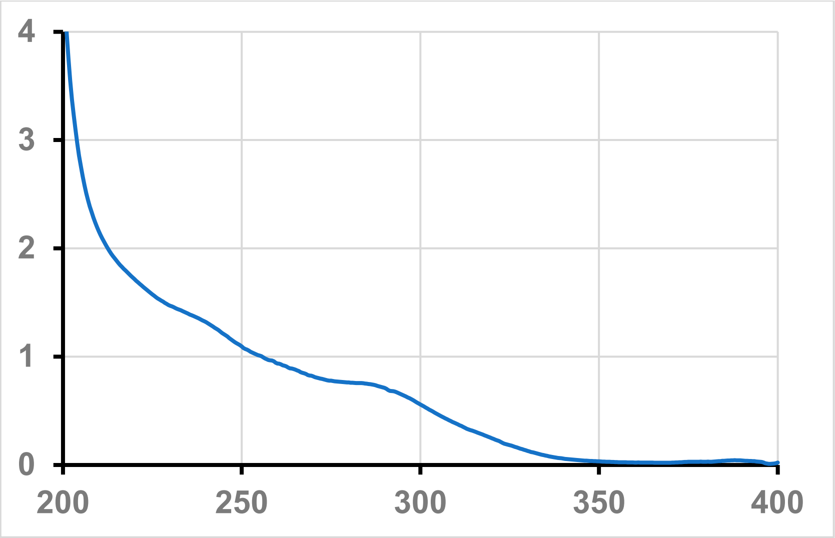

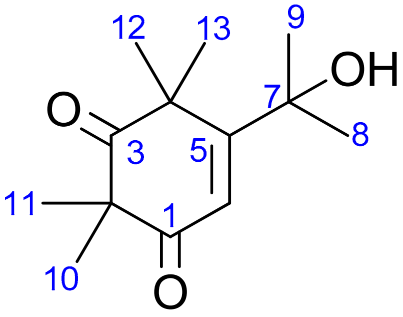

Peaks	1	2
λ_{max} (nm)	286	238
Absorbance at λ_{max}	2.60	1.35
Log [ε($M^{-1}cm^{-1}$)]	4.21	4.48

Figure S7. UV spectrum of subulatone A (**1**).

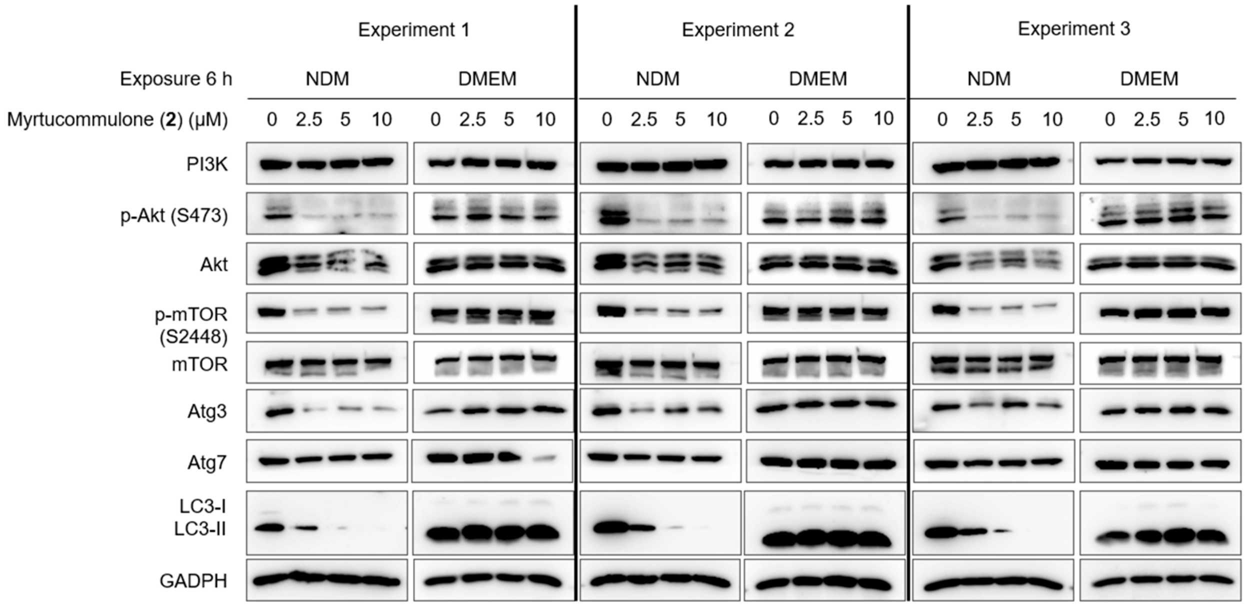

Figure S8. Three independent experiments on the effect of myrtucommulone A (**2**) on proteins related to the PI3K/Akt/mTOR and autophagy signaling pathways in NDM and DMEM.
