# Supplementary figures and images for: Chemical Constituents of Callistemon subulatus and Their Anti-Pancreatic Cancer Activity against Human PANC-1 Cell Line
Source: Plants (Basel). 2022 Sep 21;11(19):2466. doi: 10.3390/plants11192466 (PMC9570665; doi:10.3390/plants11192466)

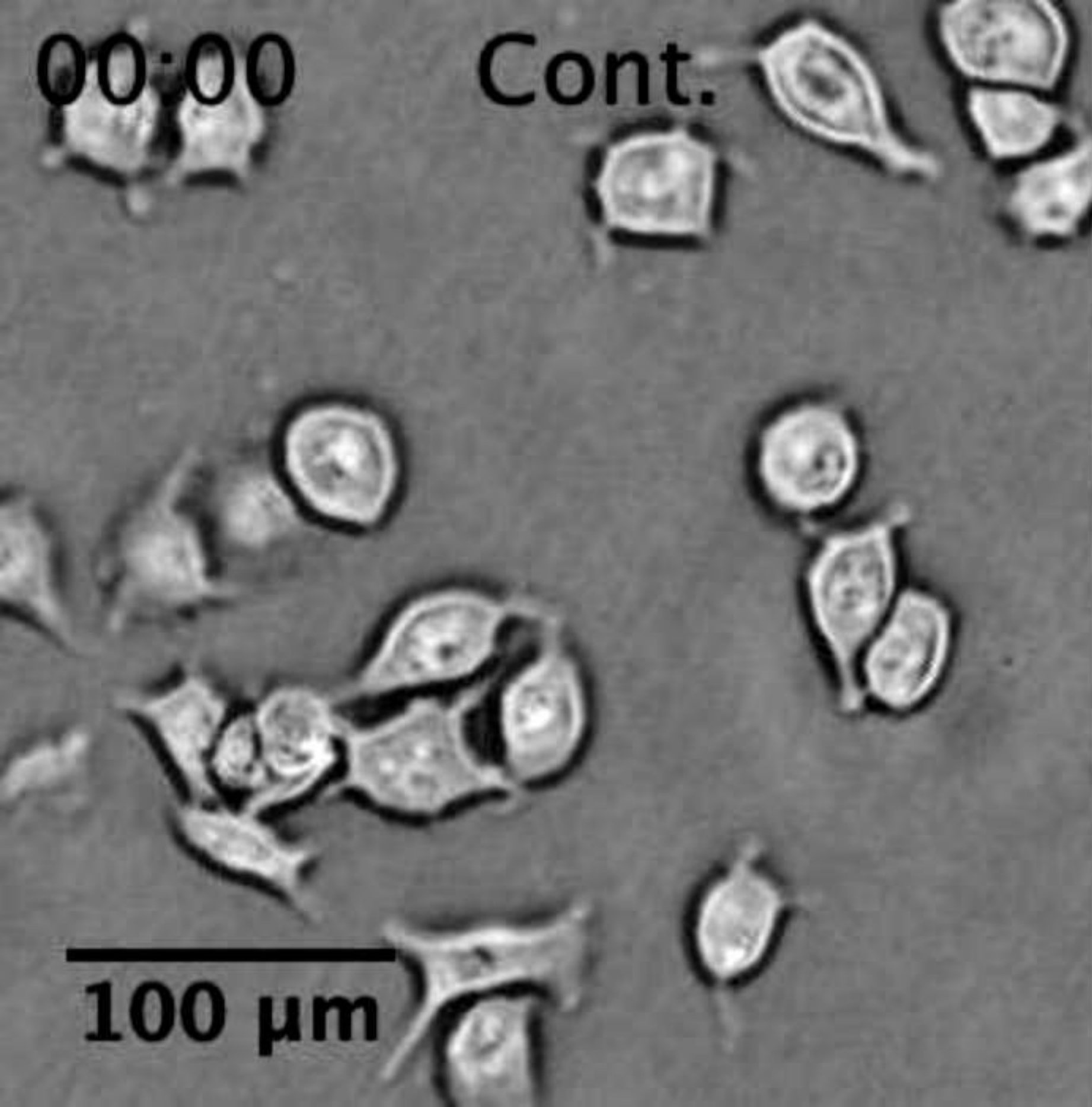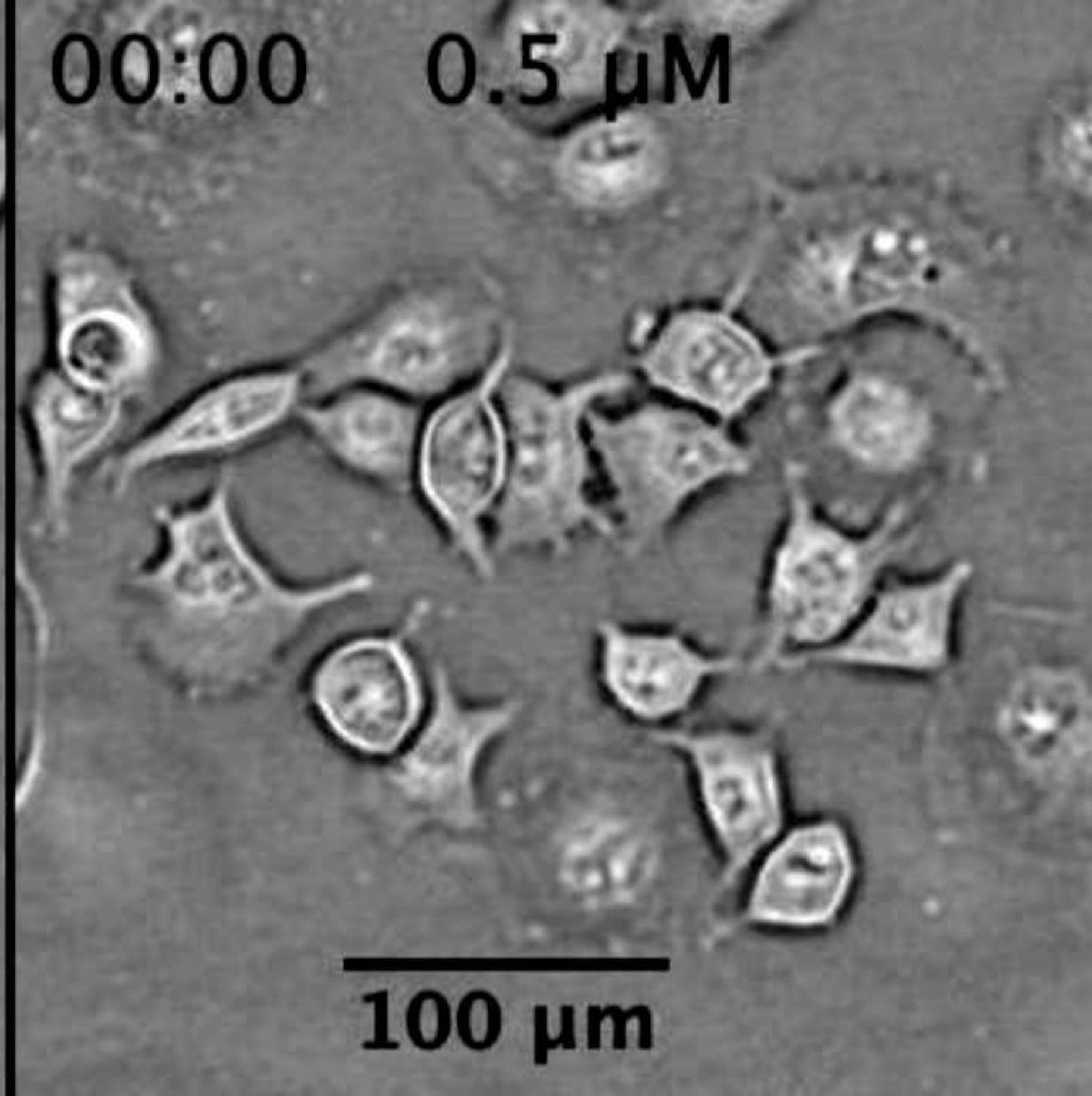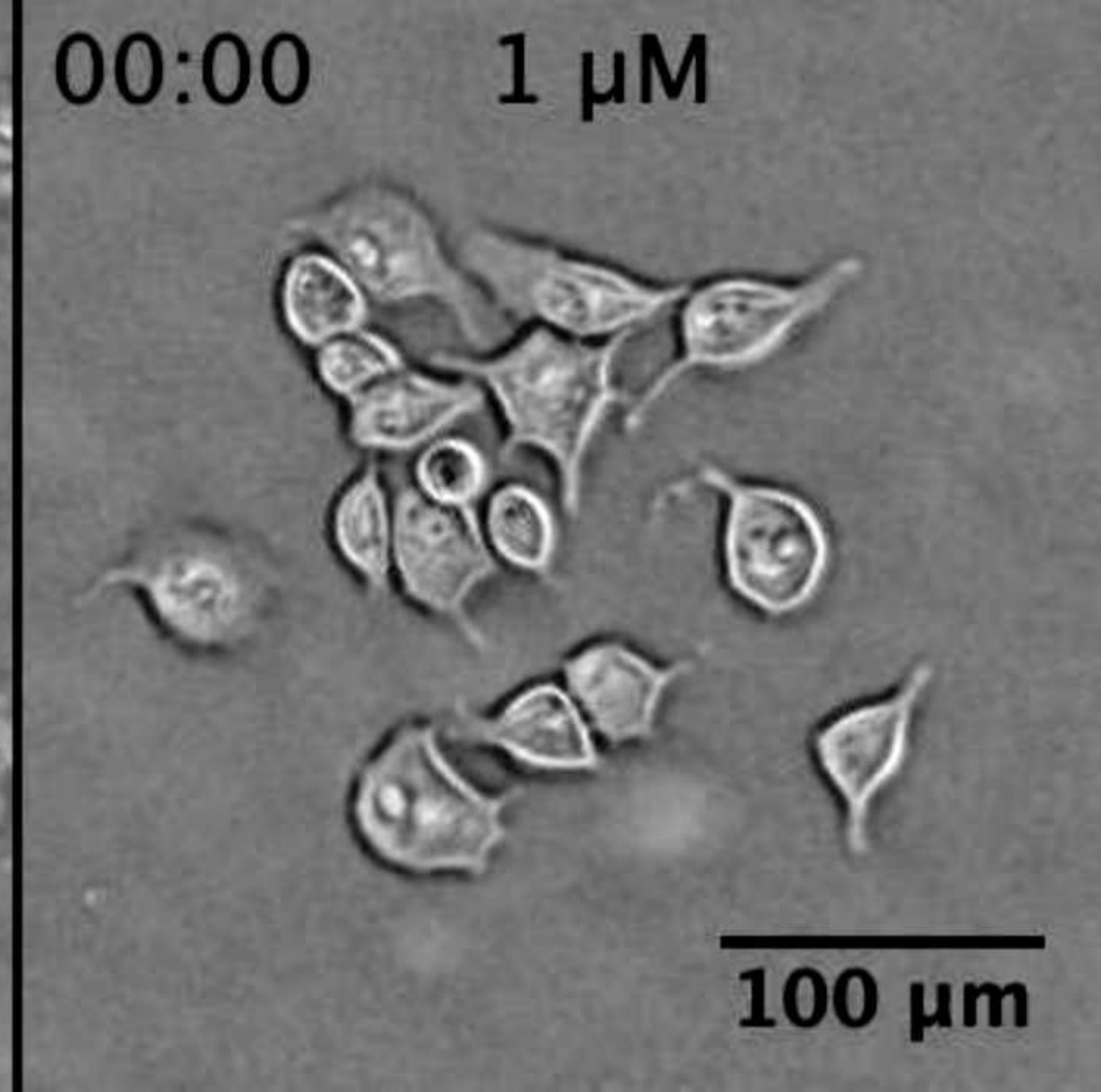

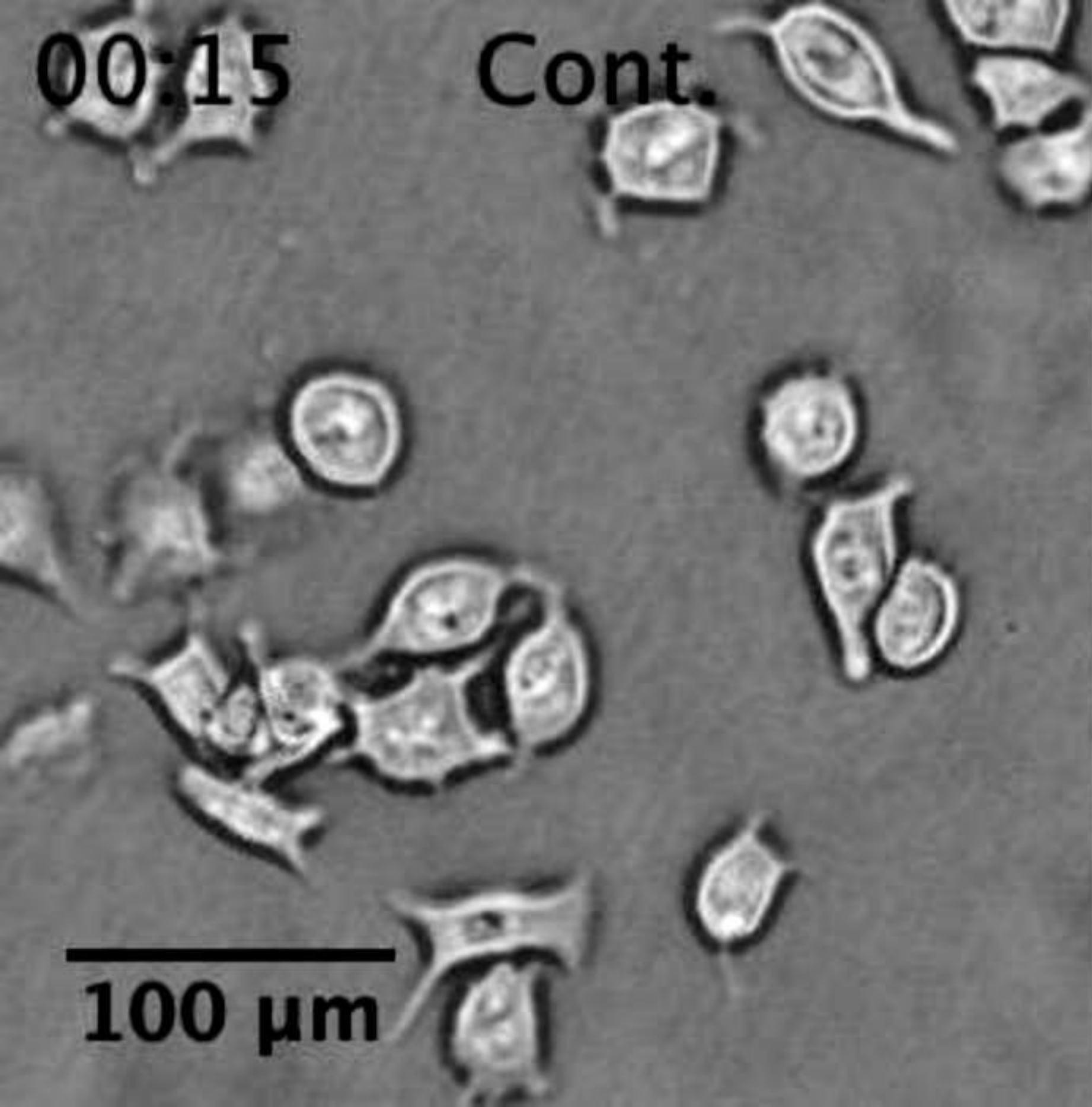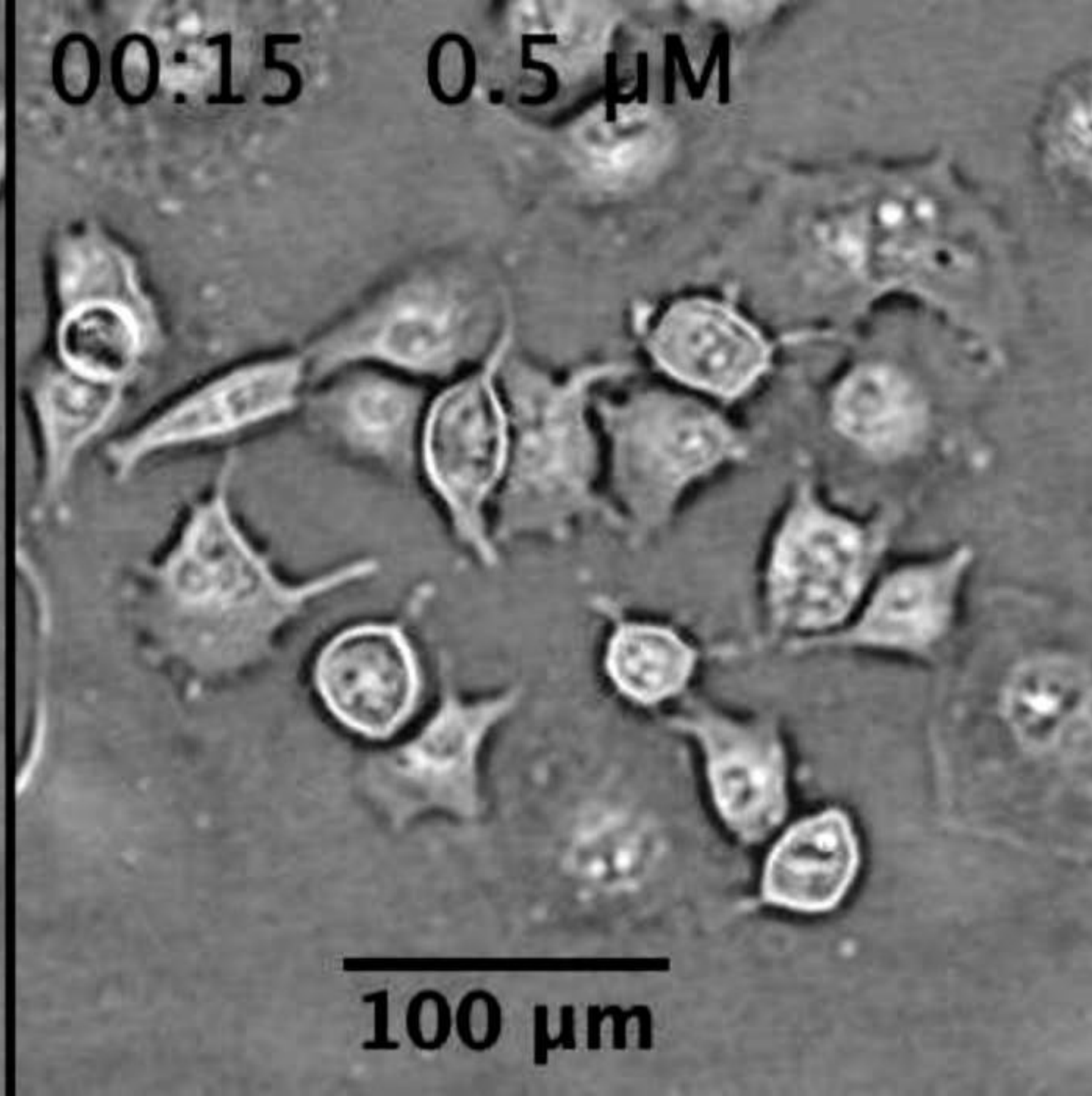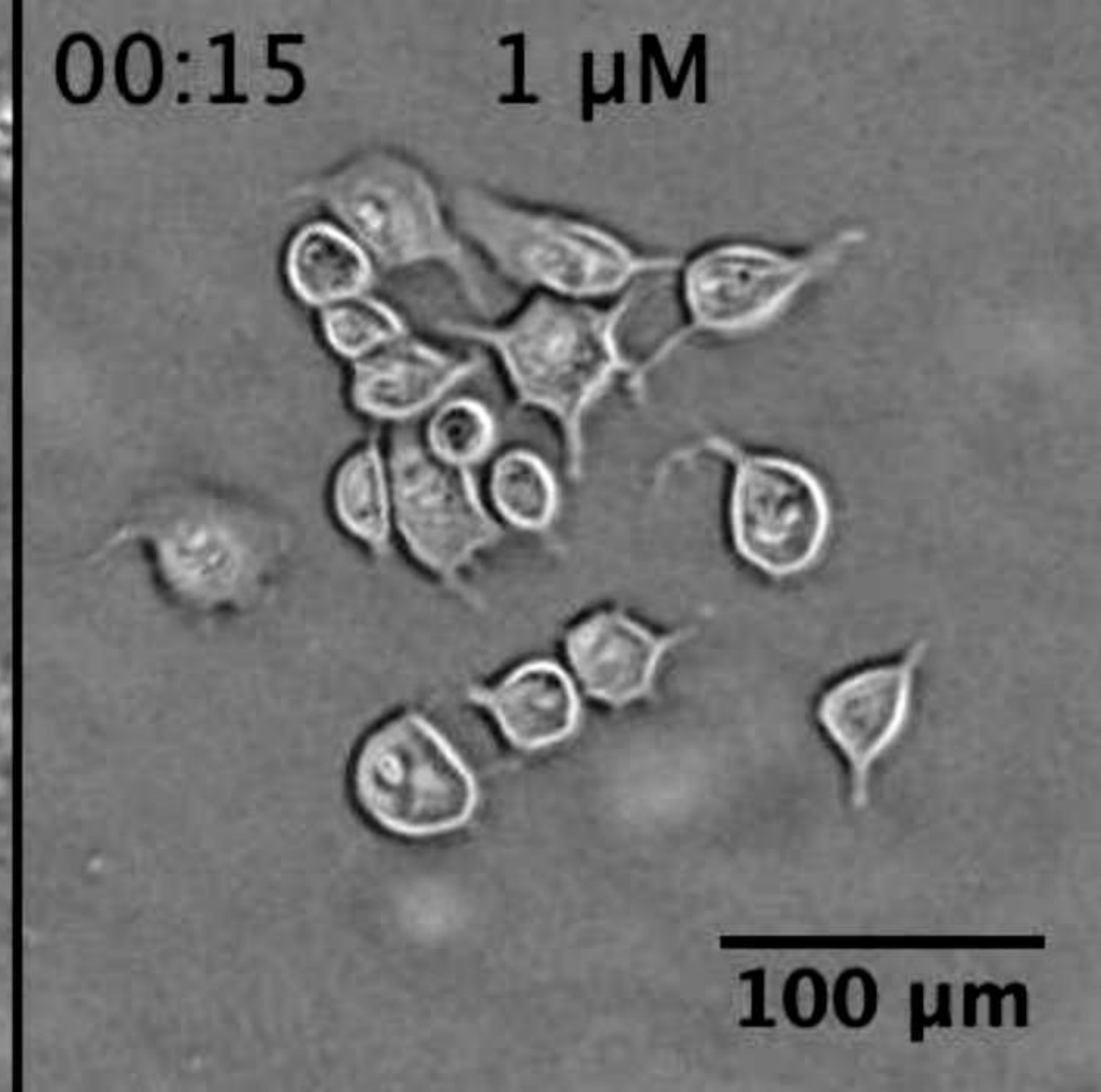

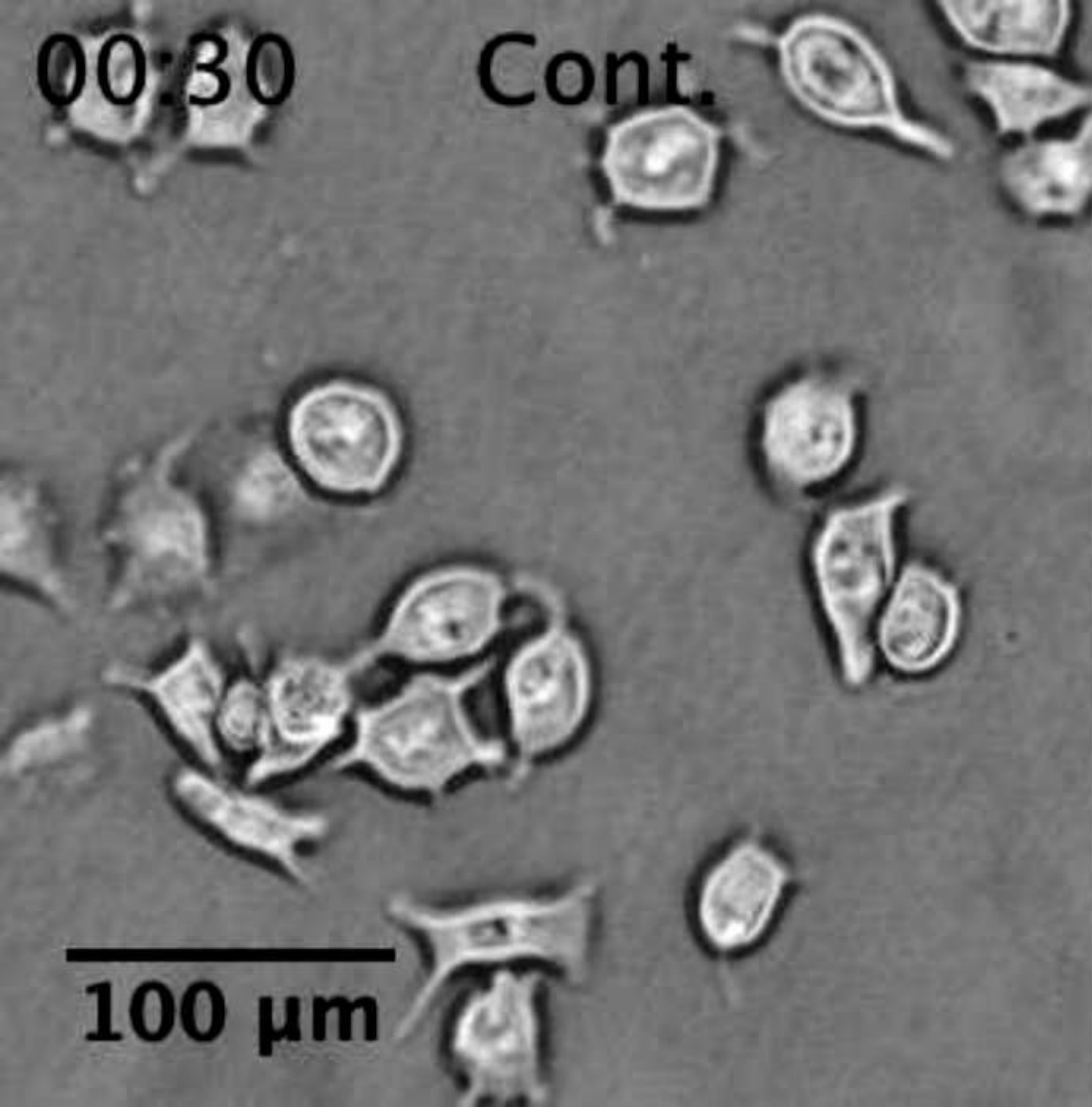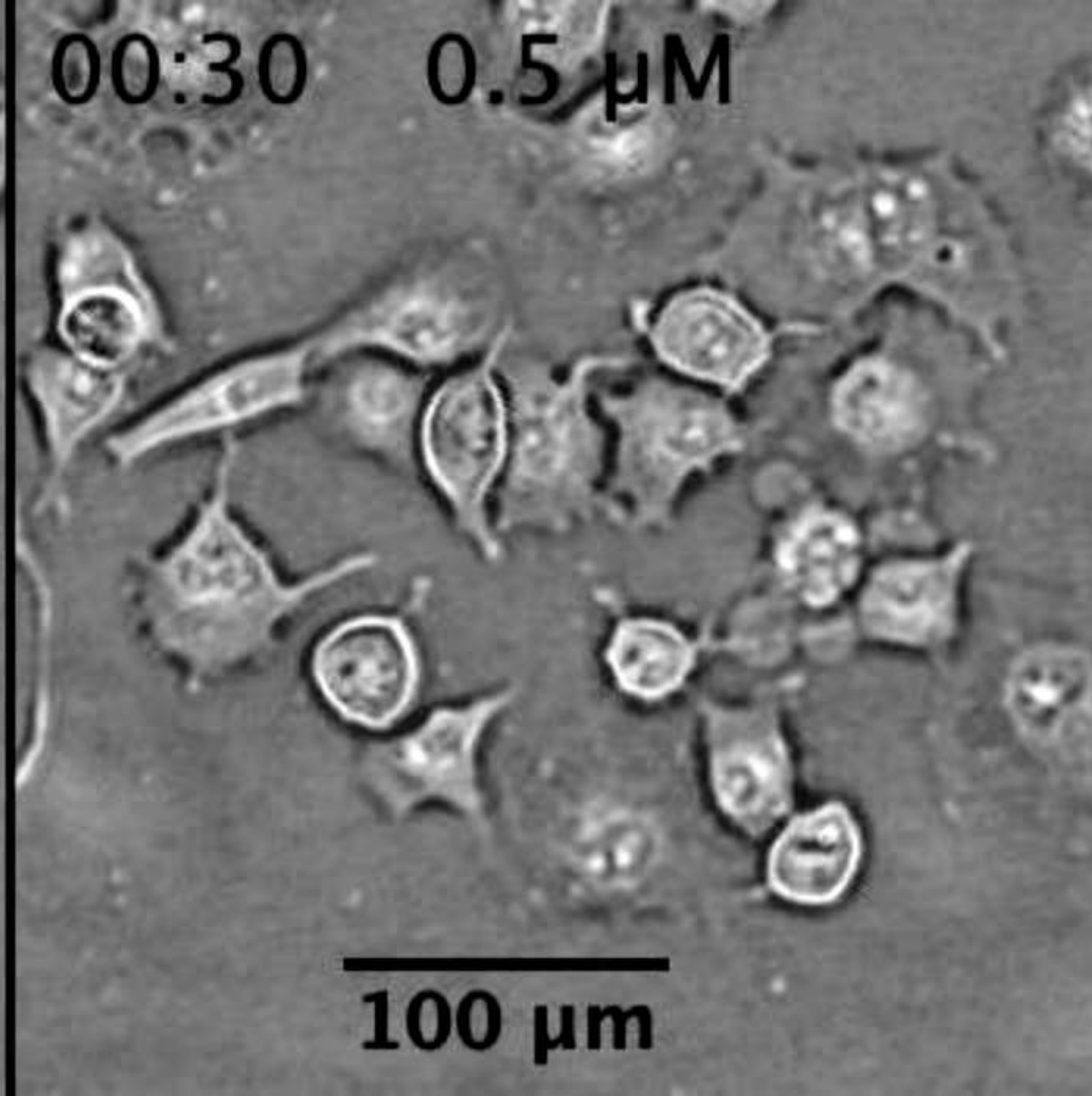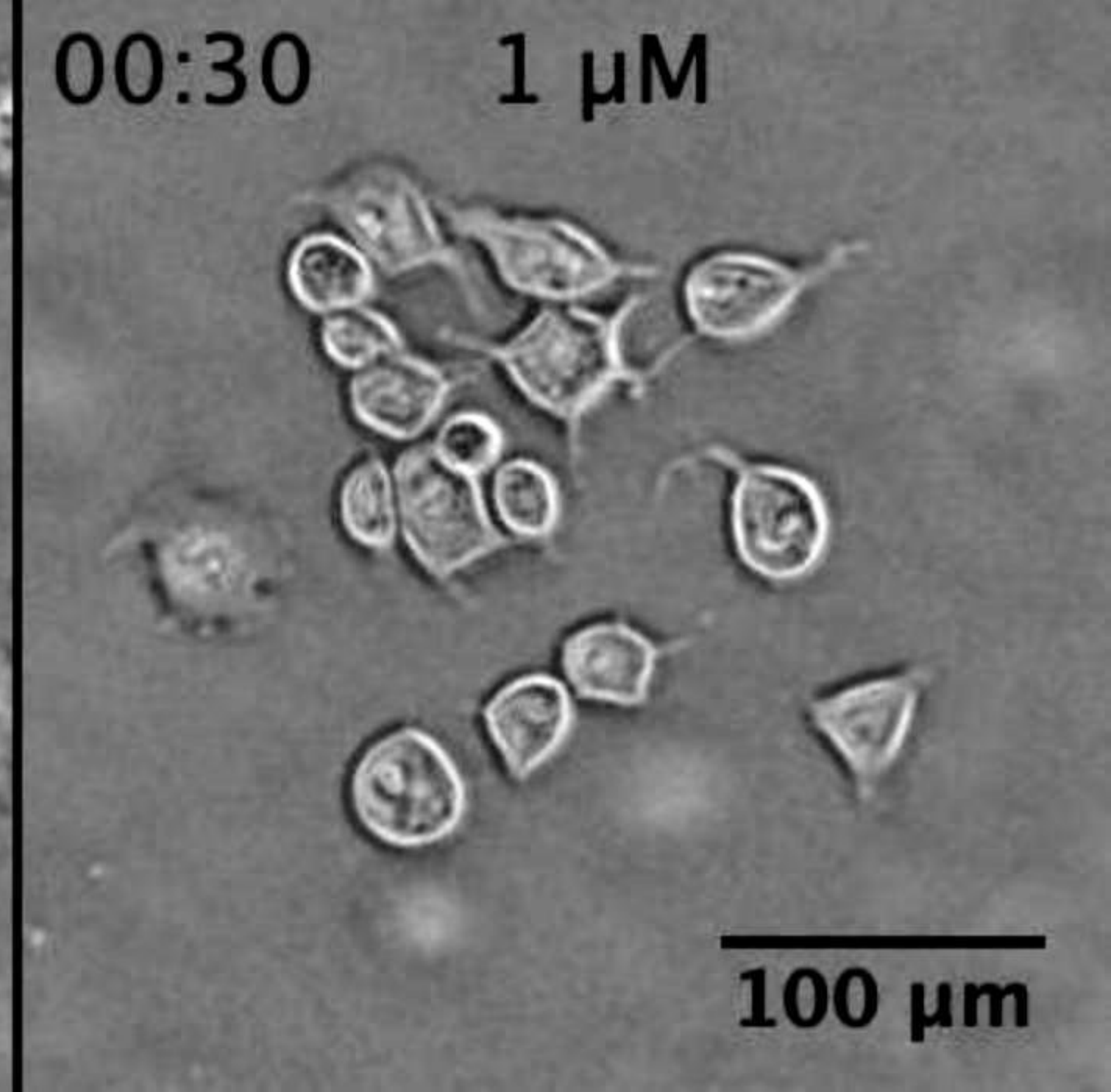

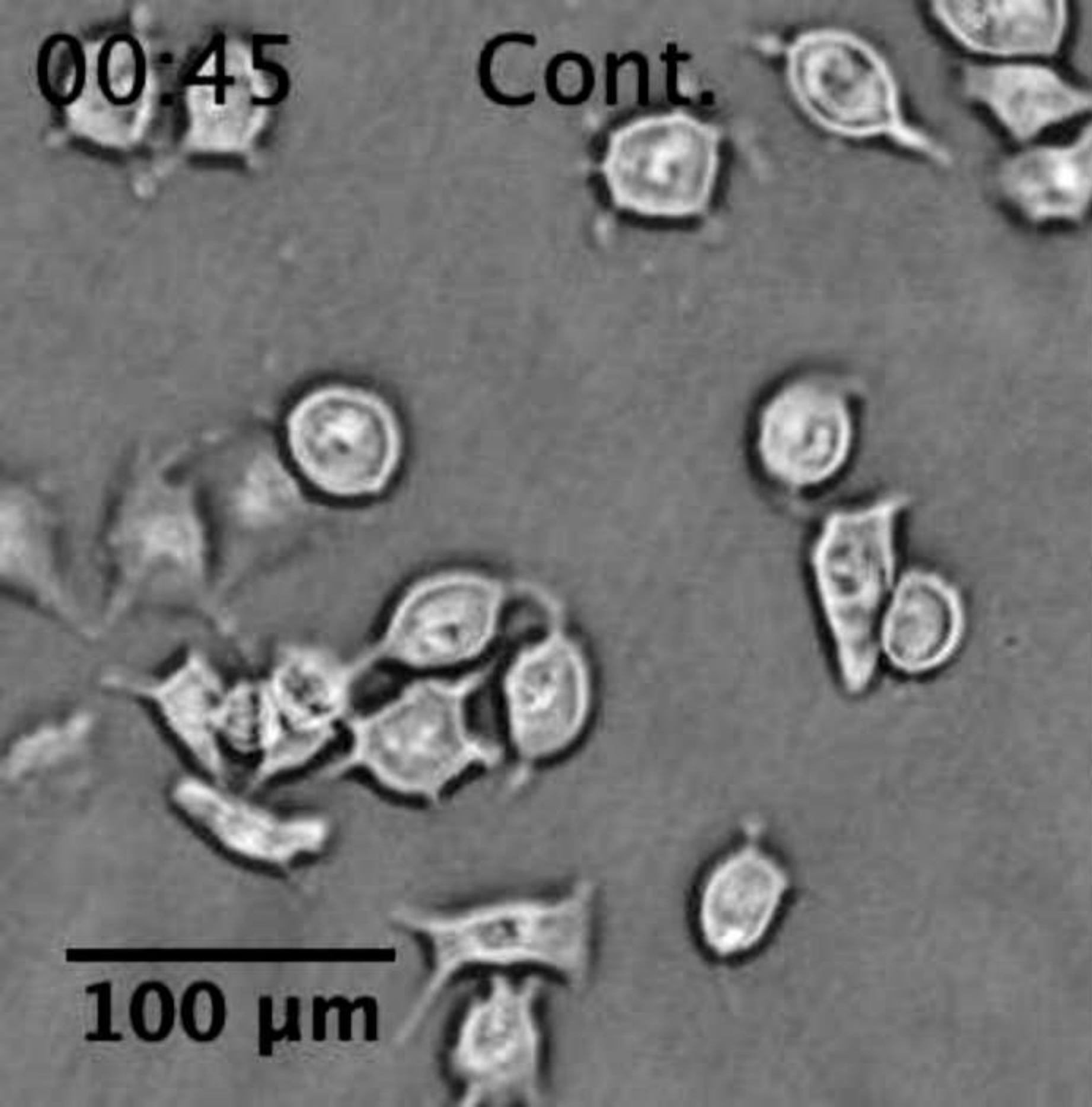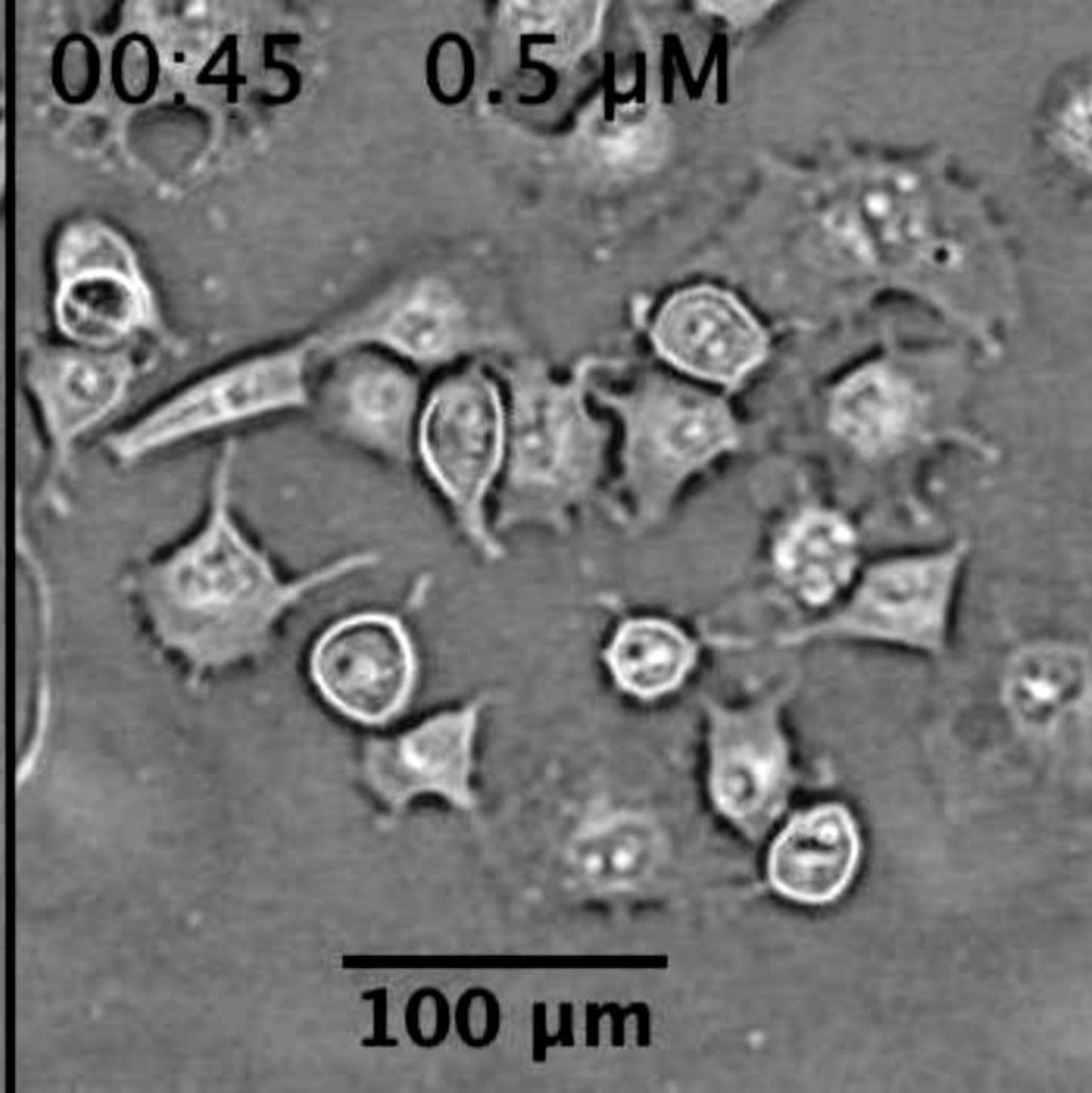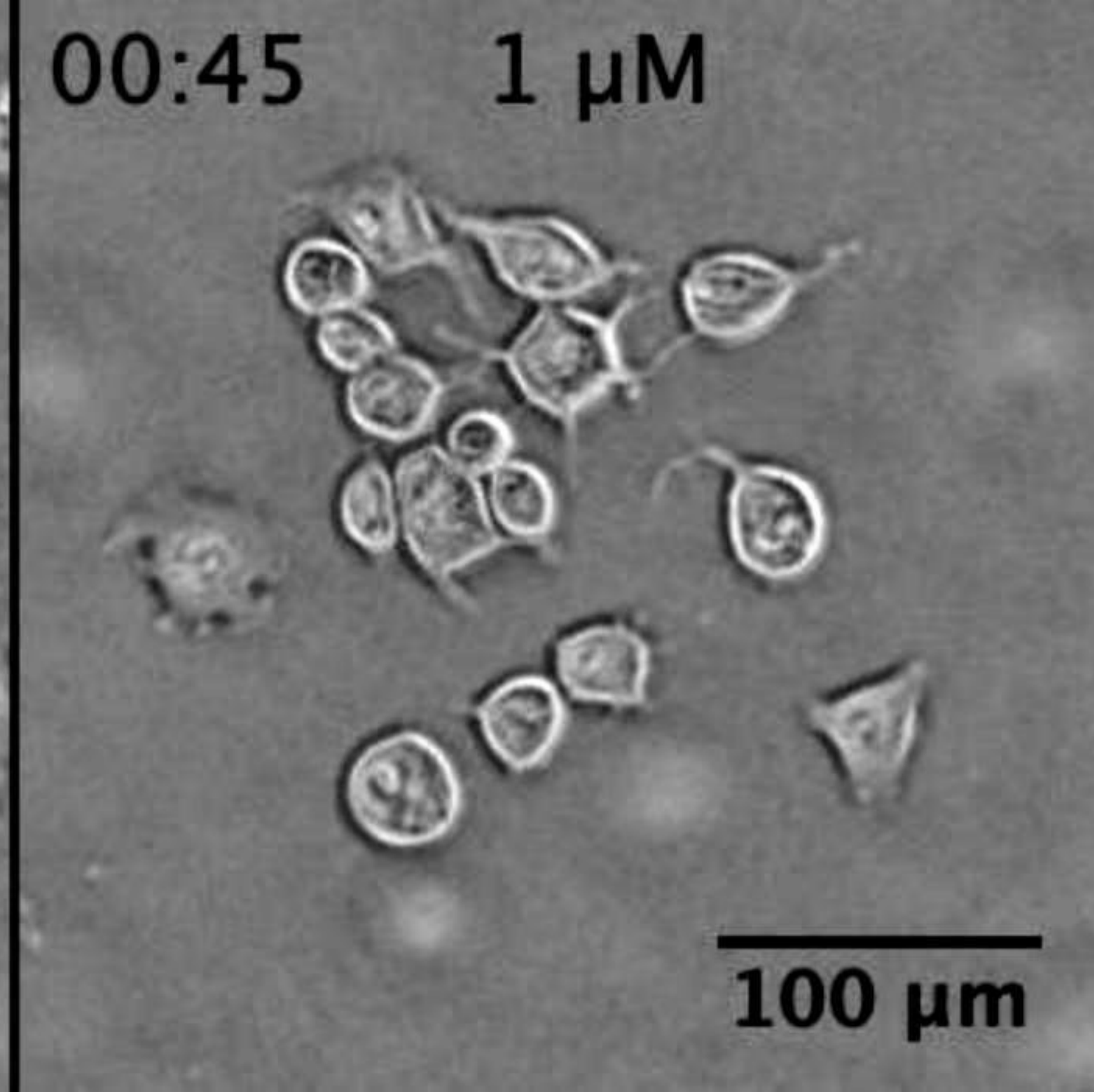

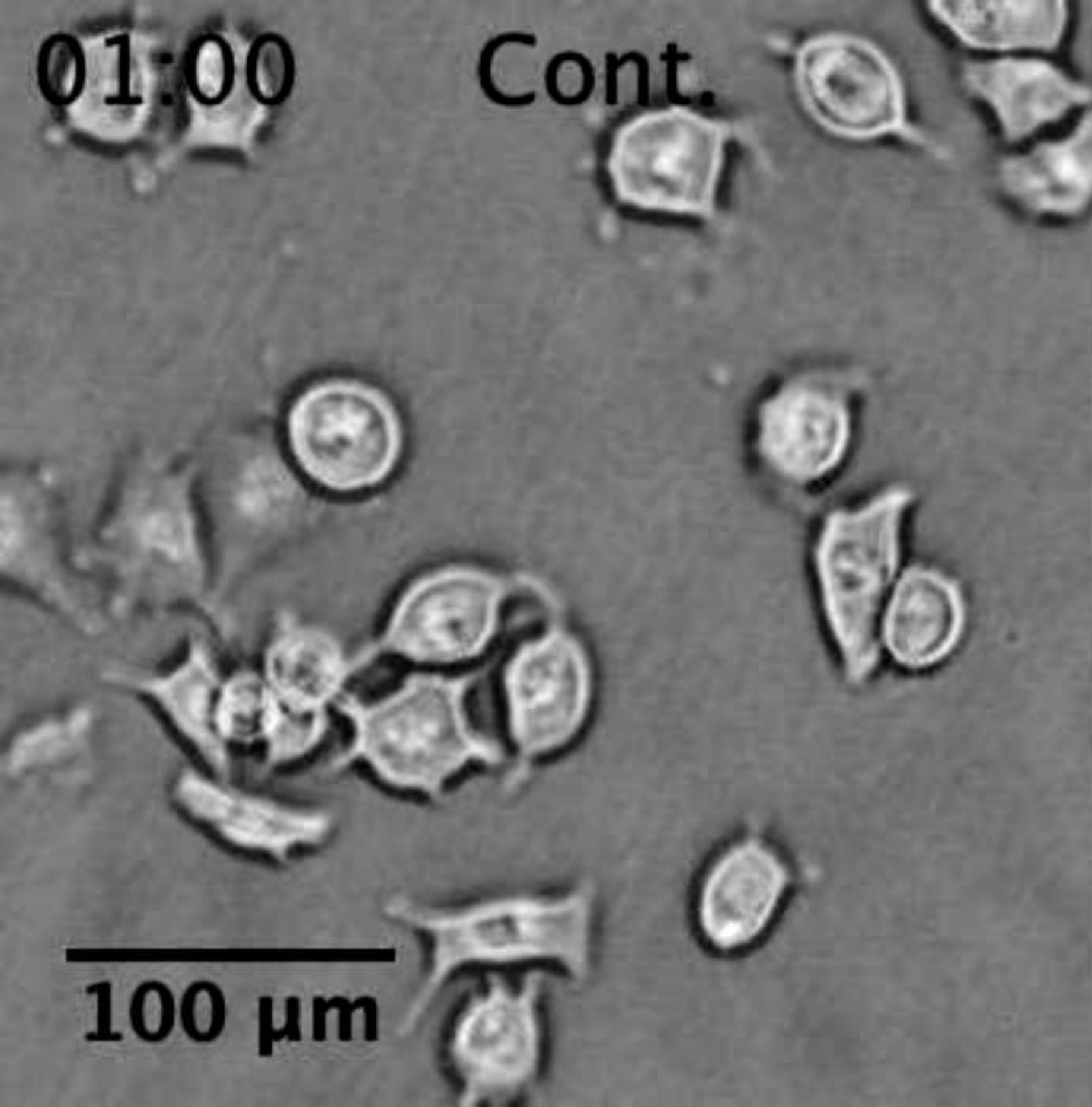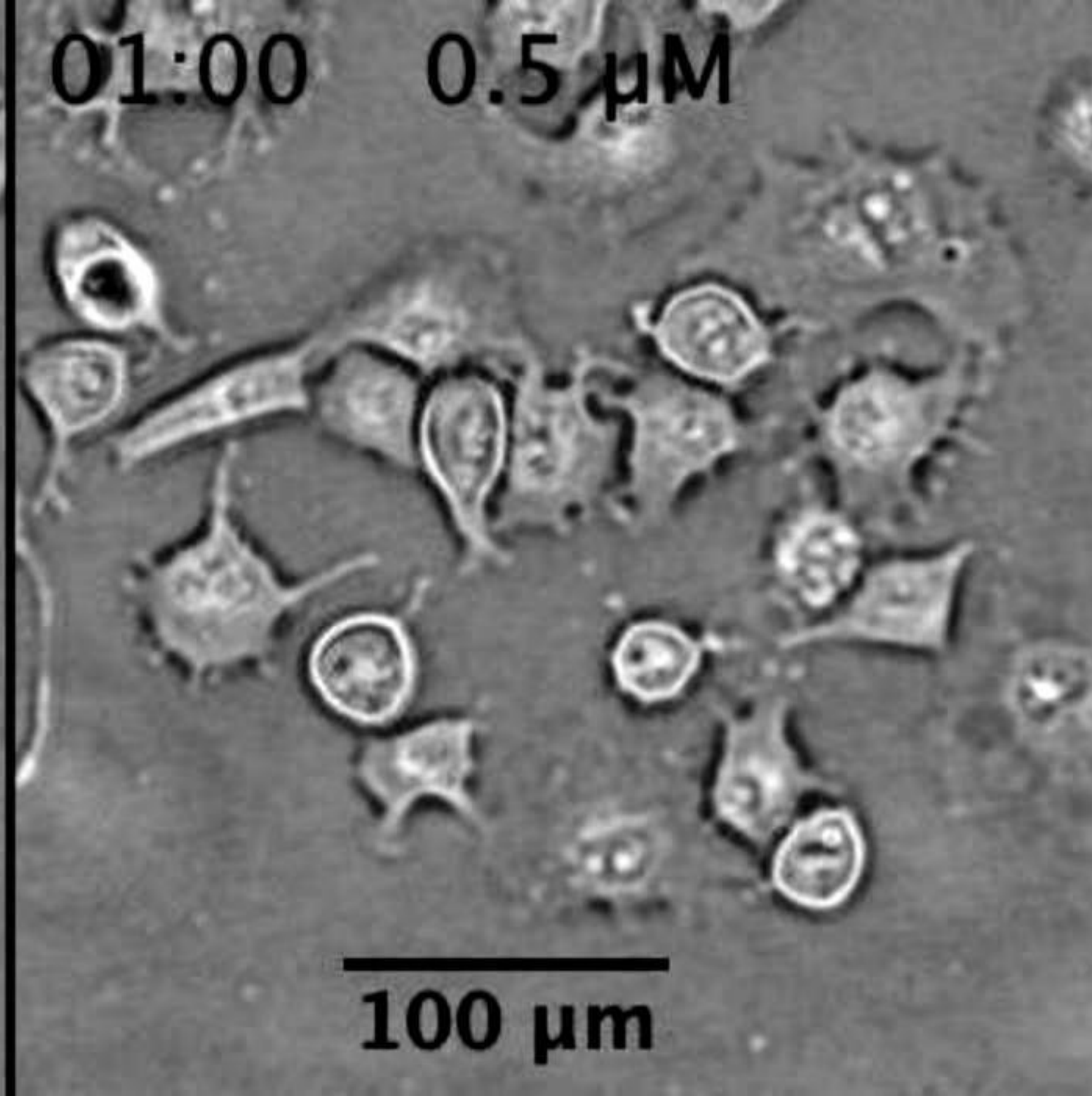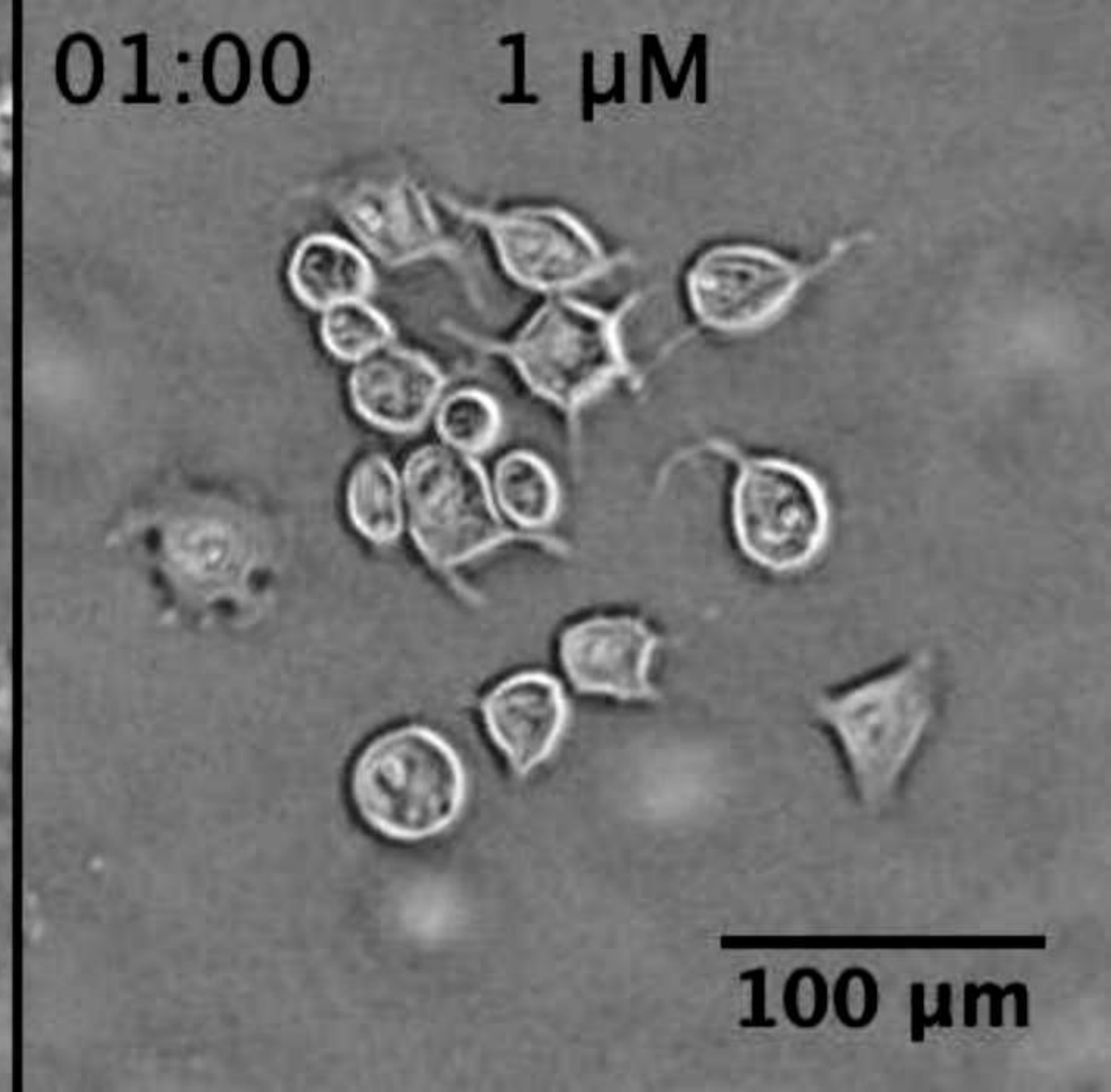

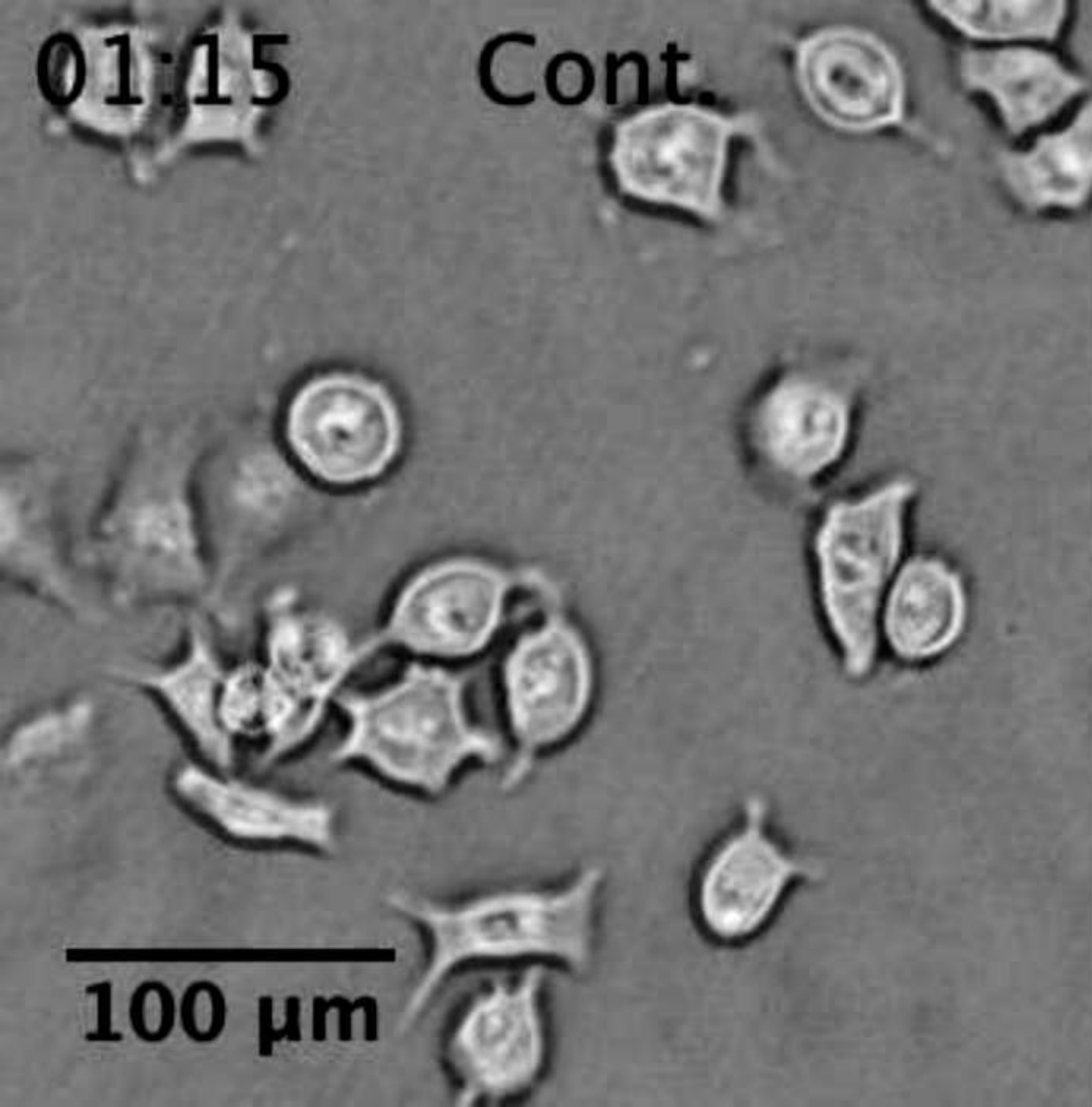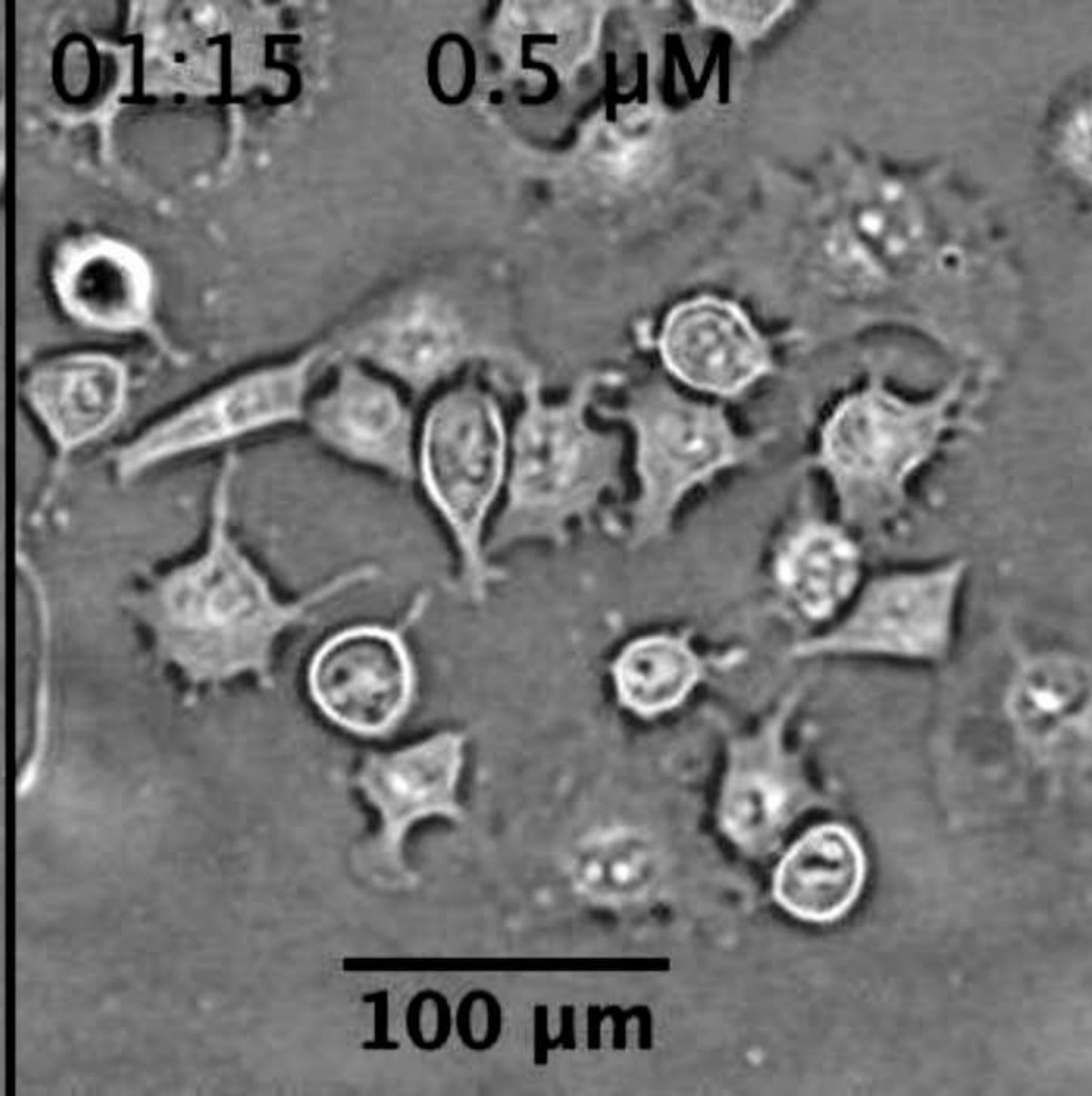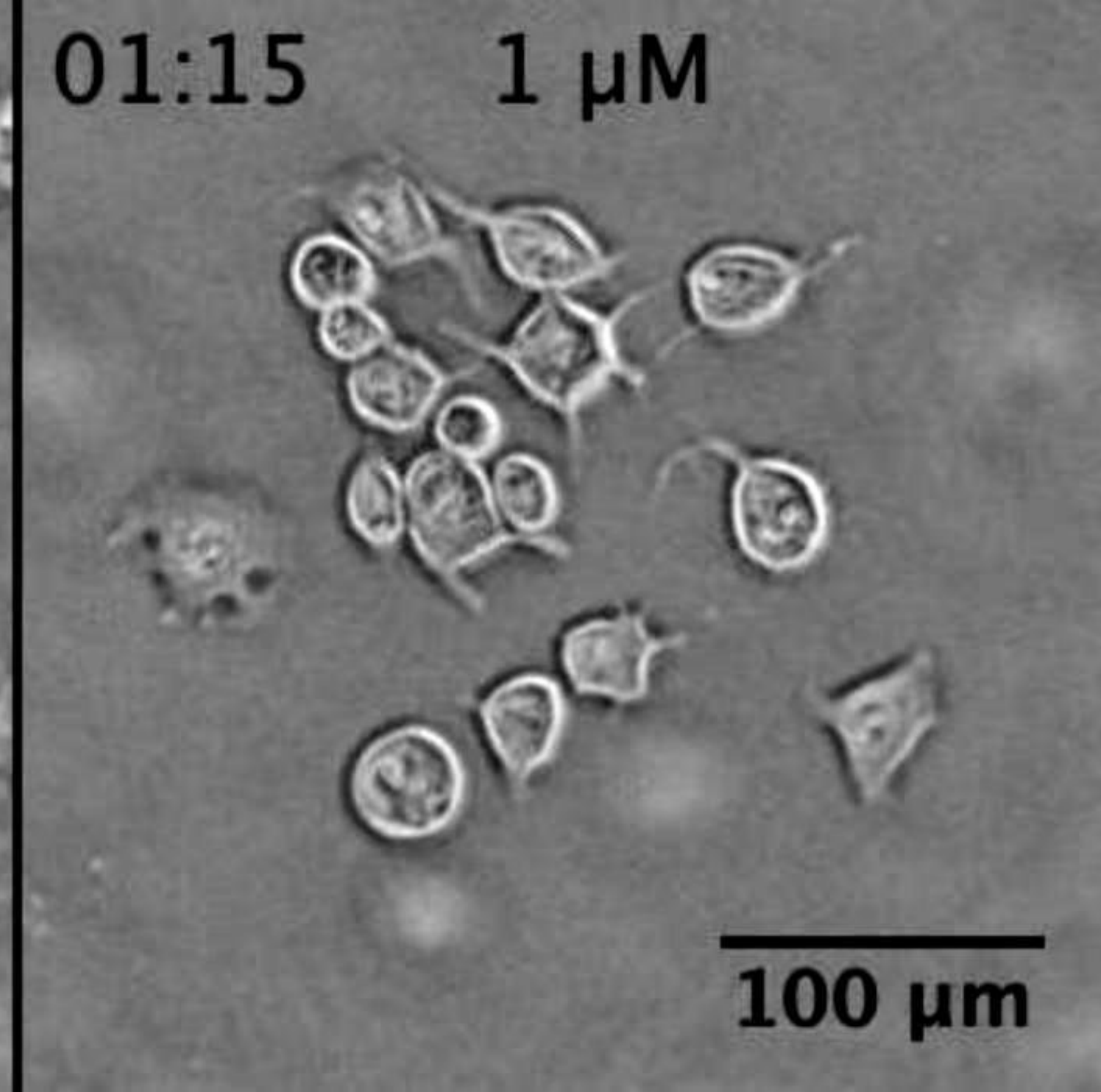

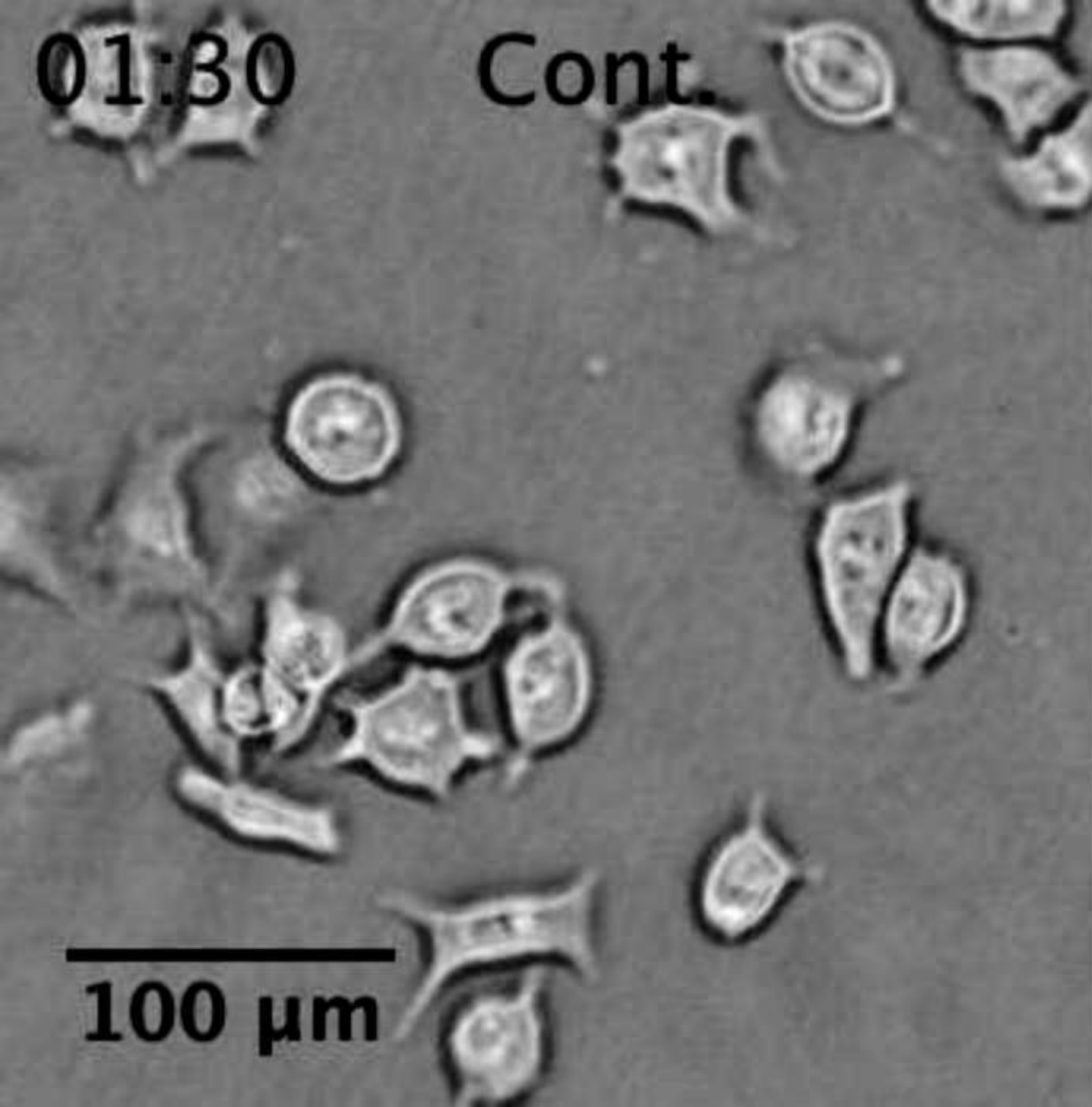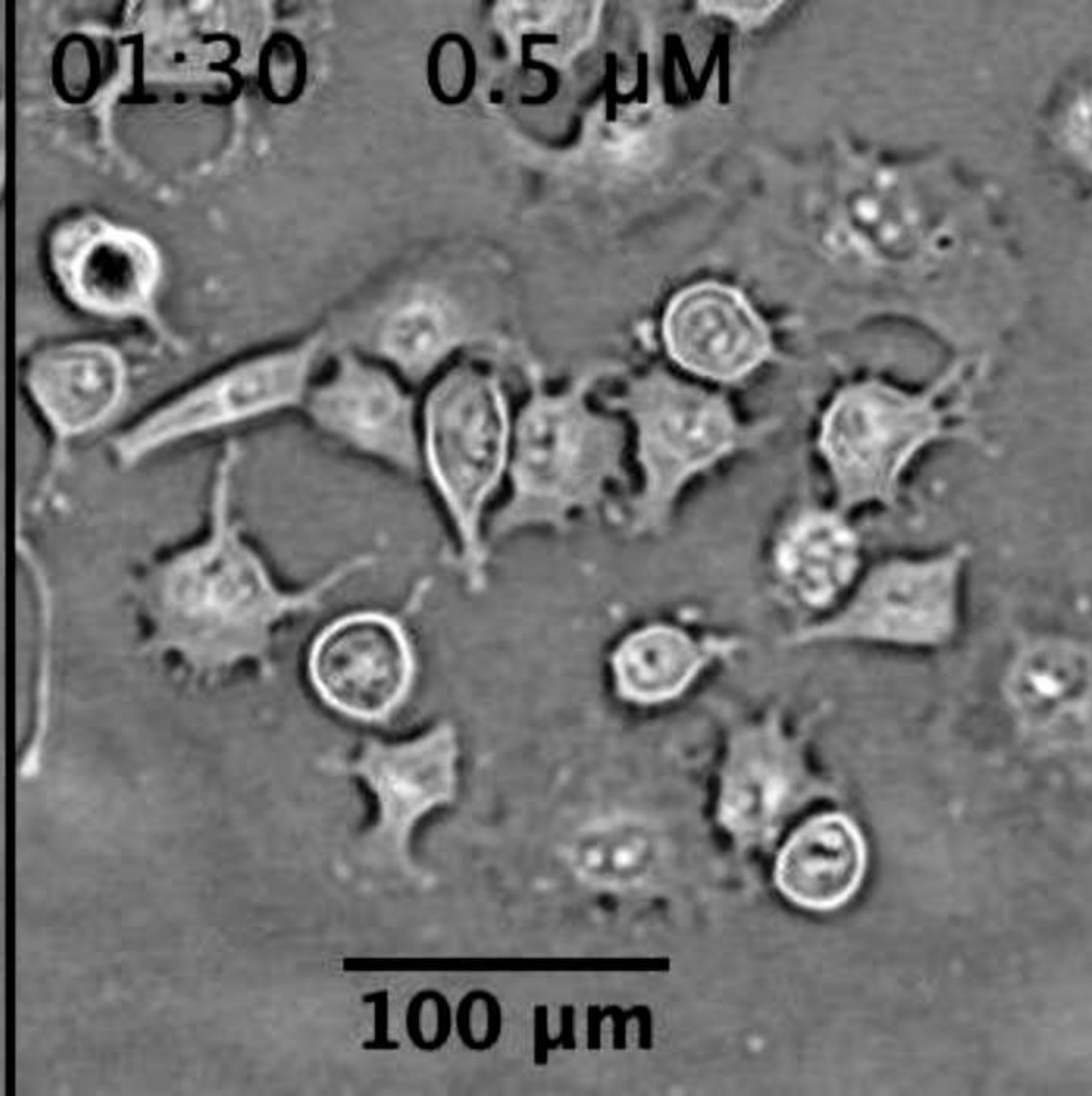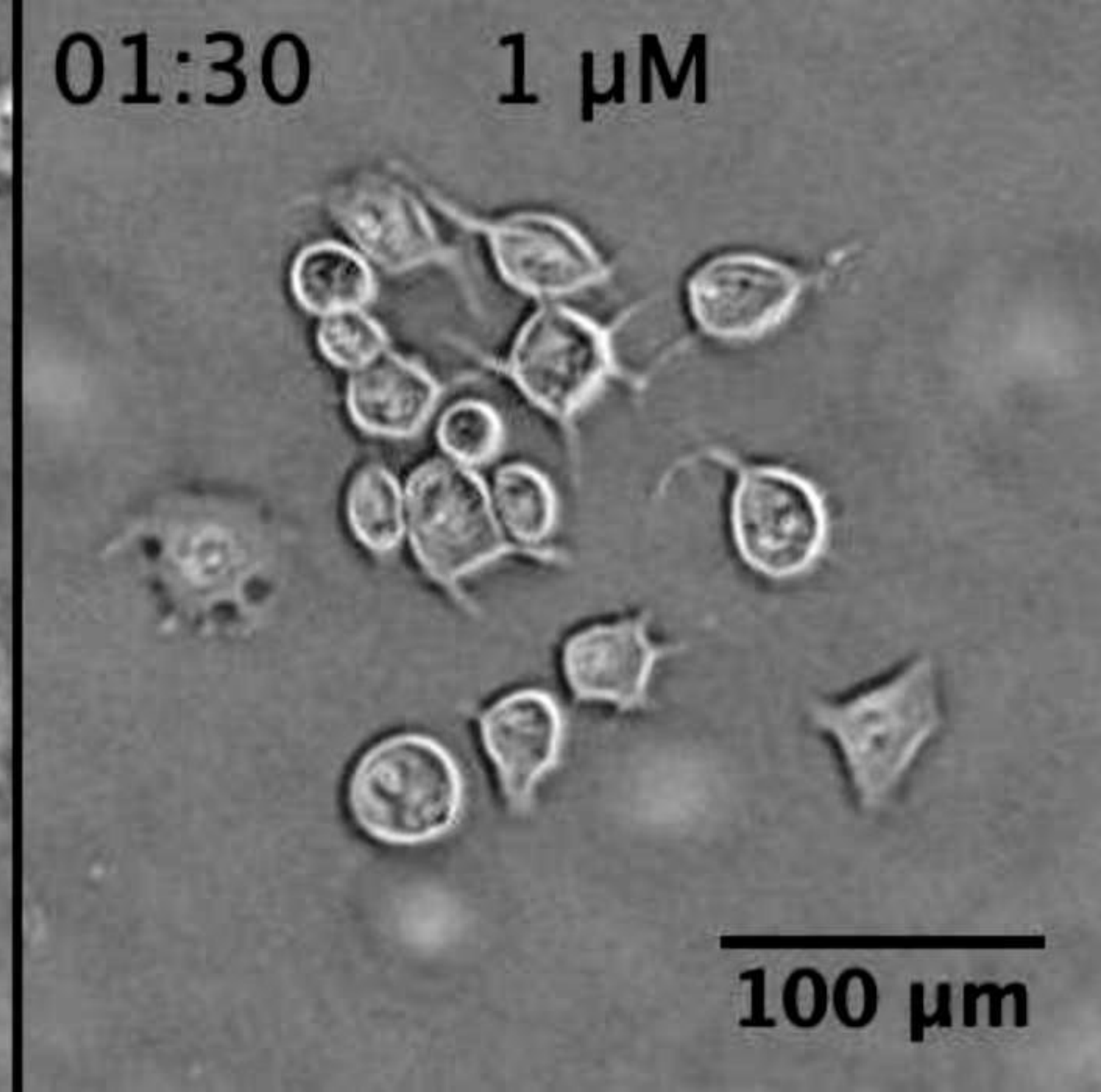

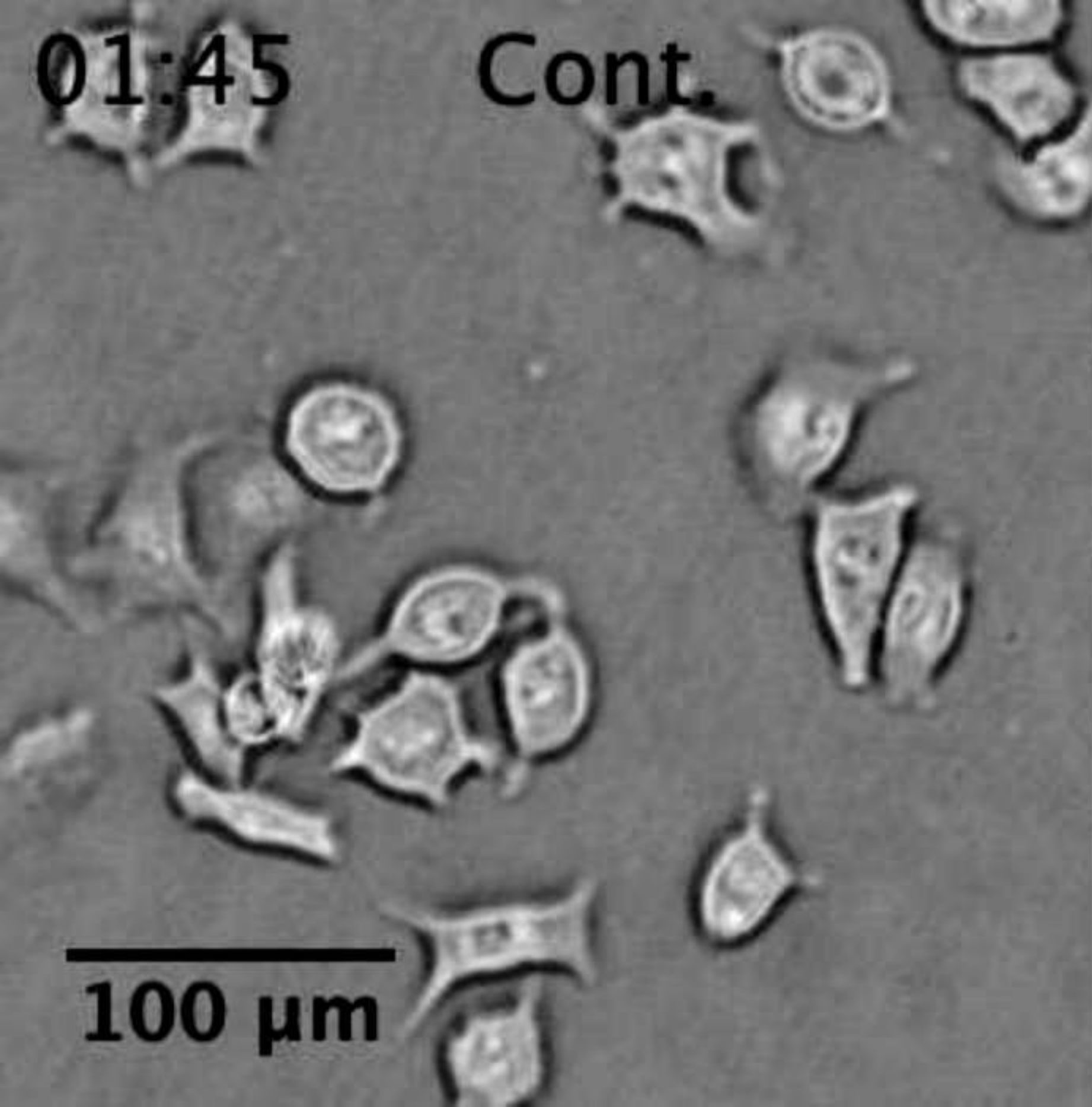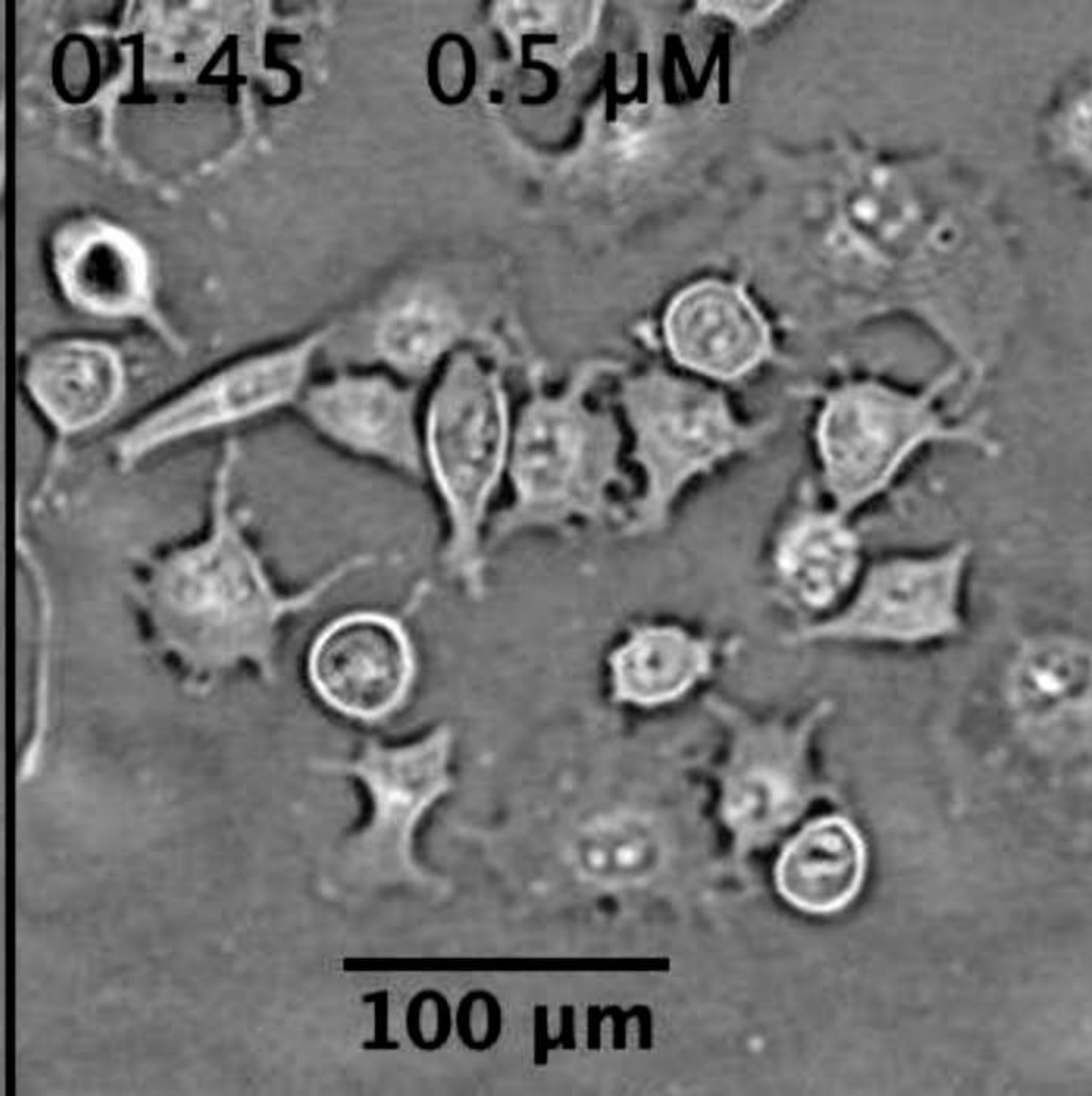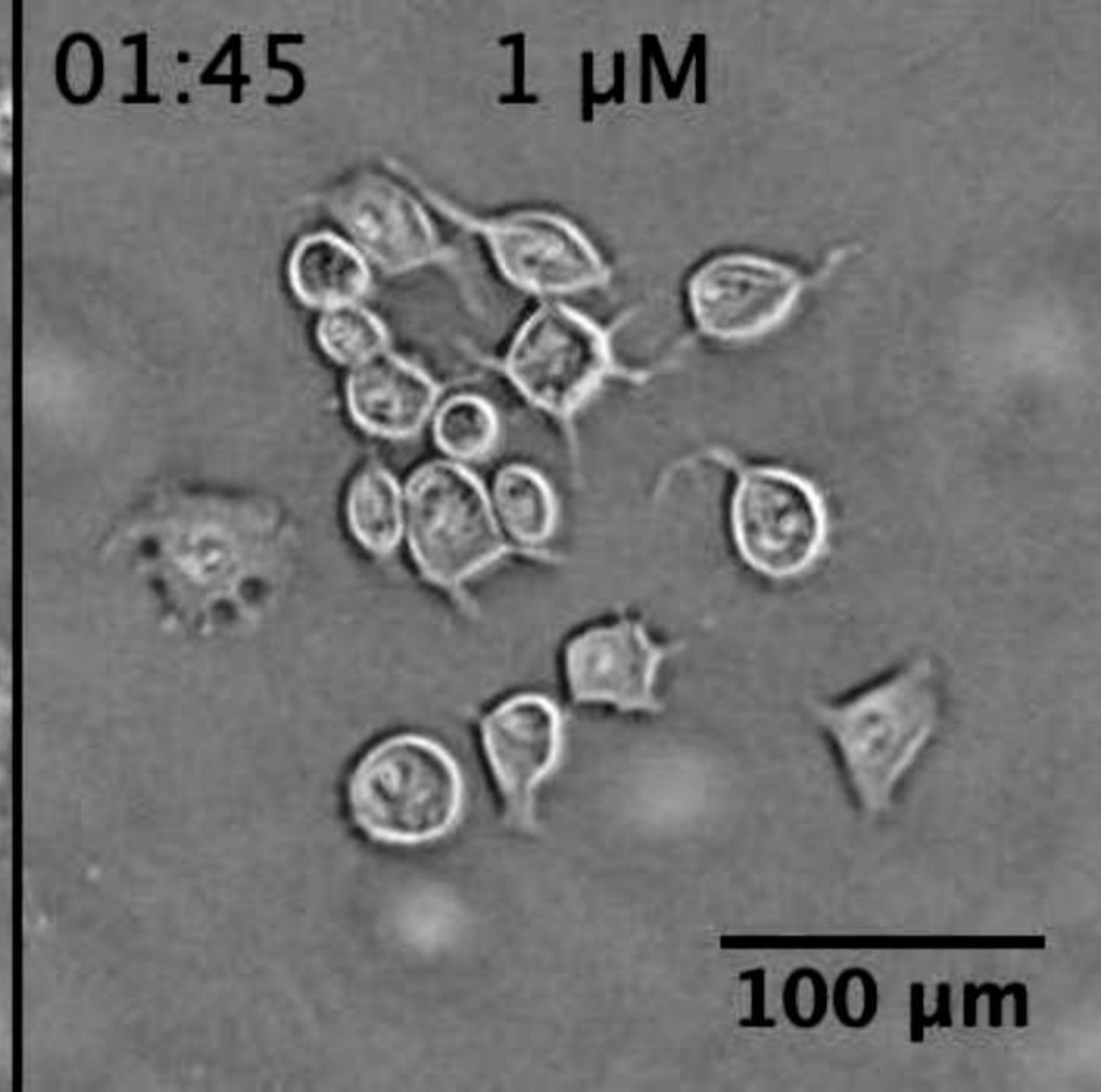

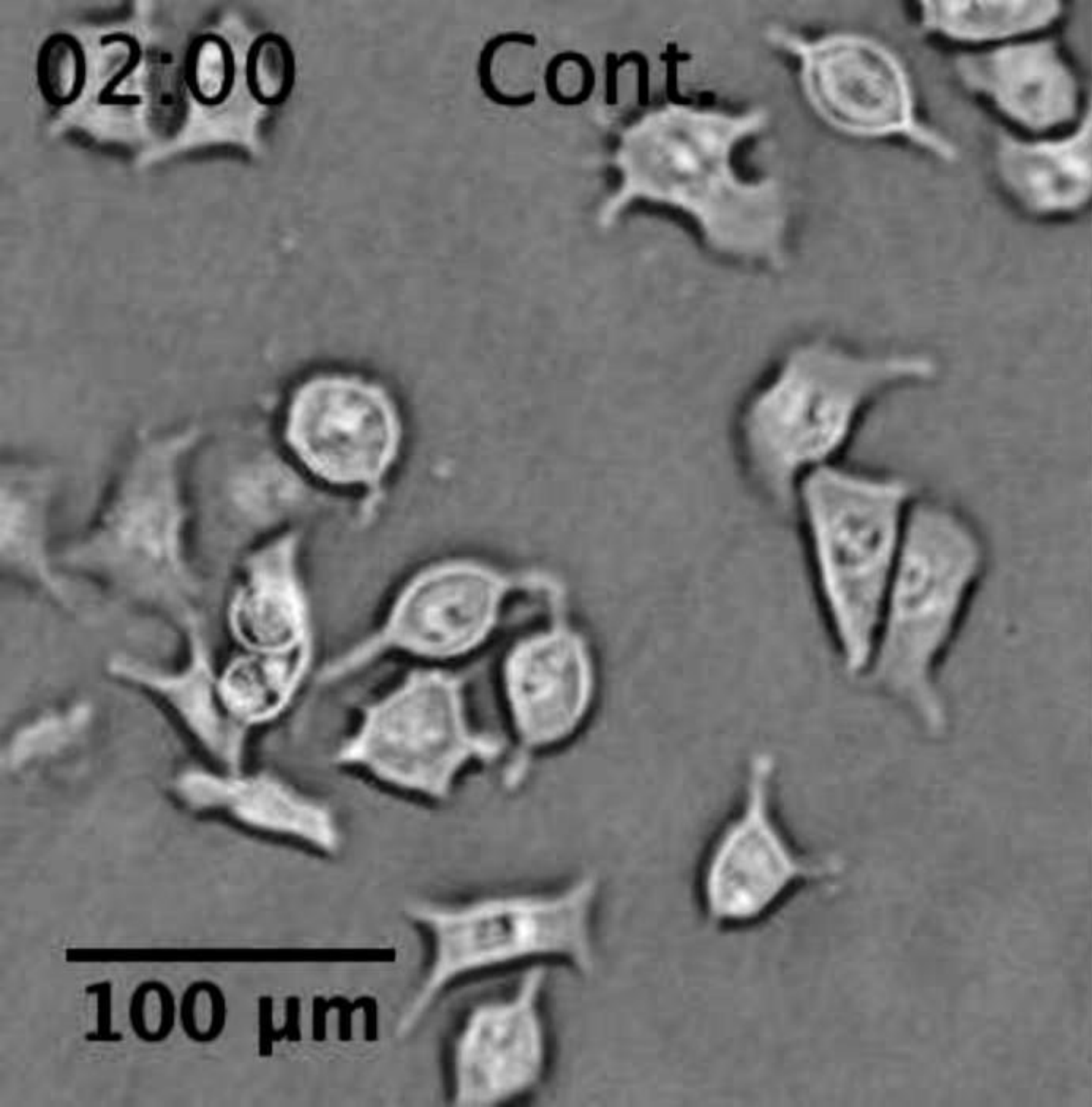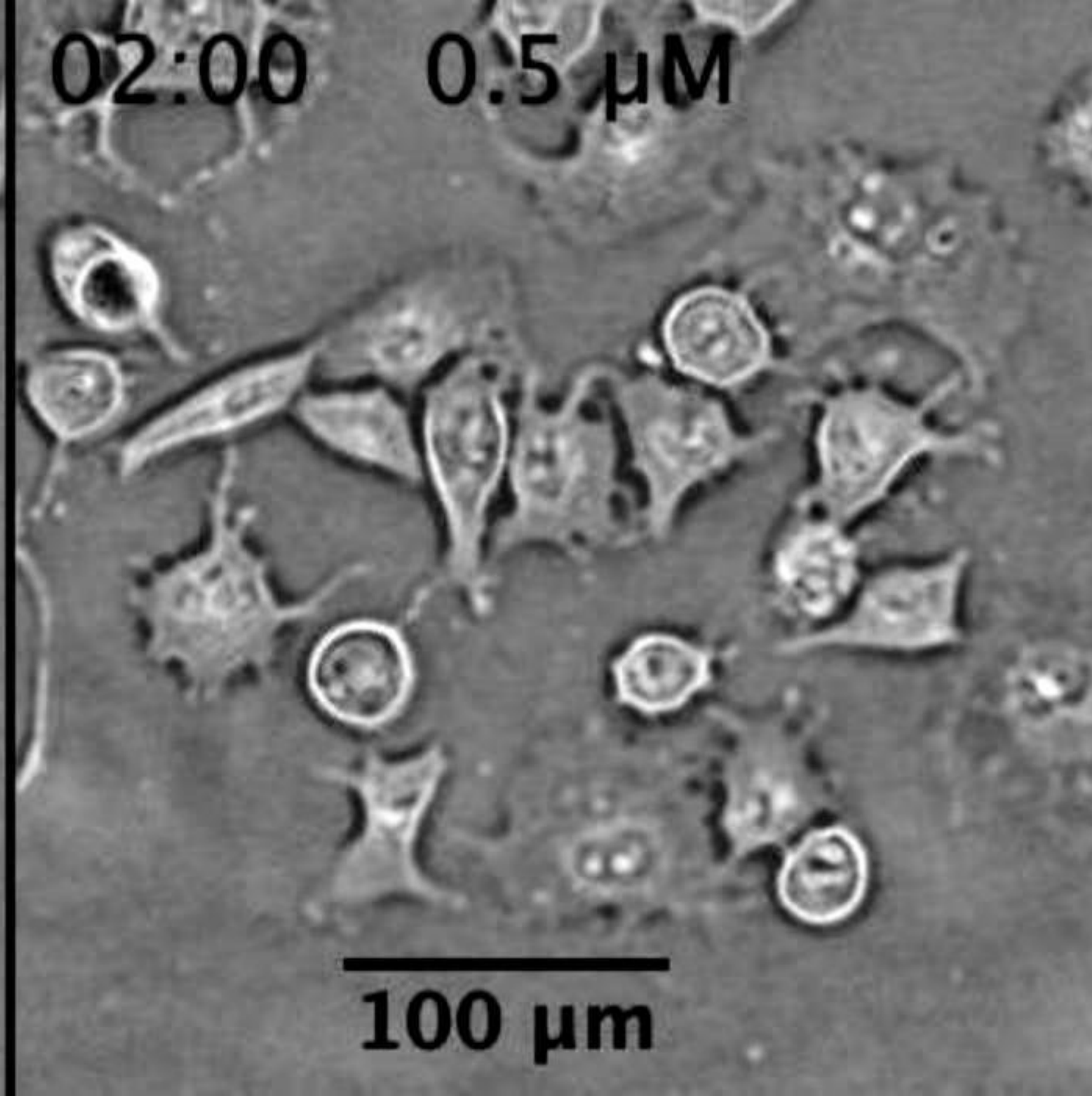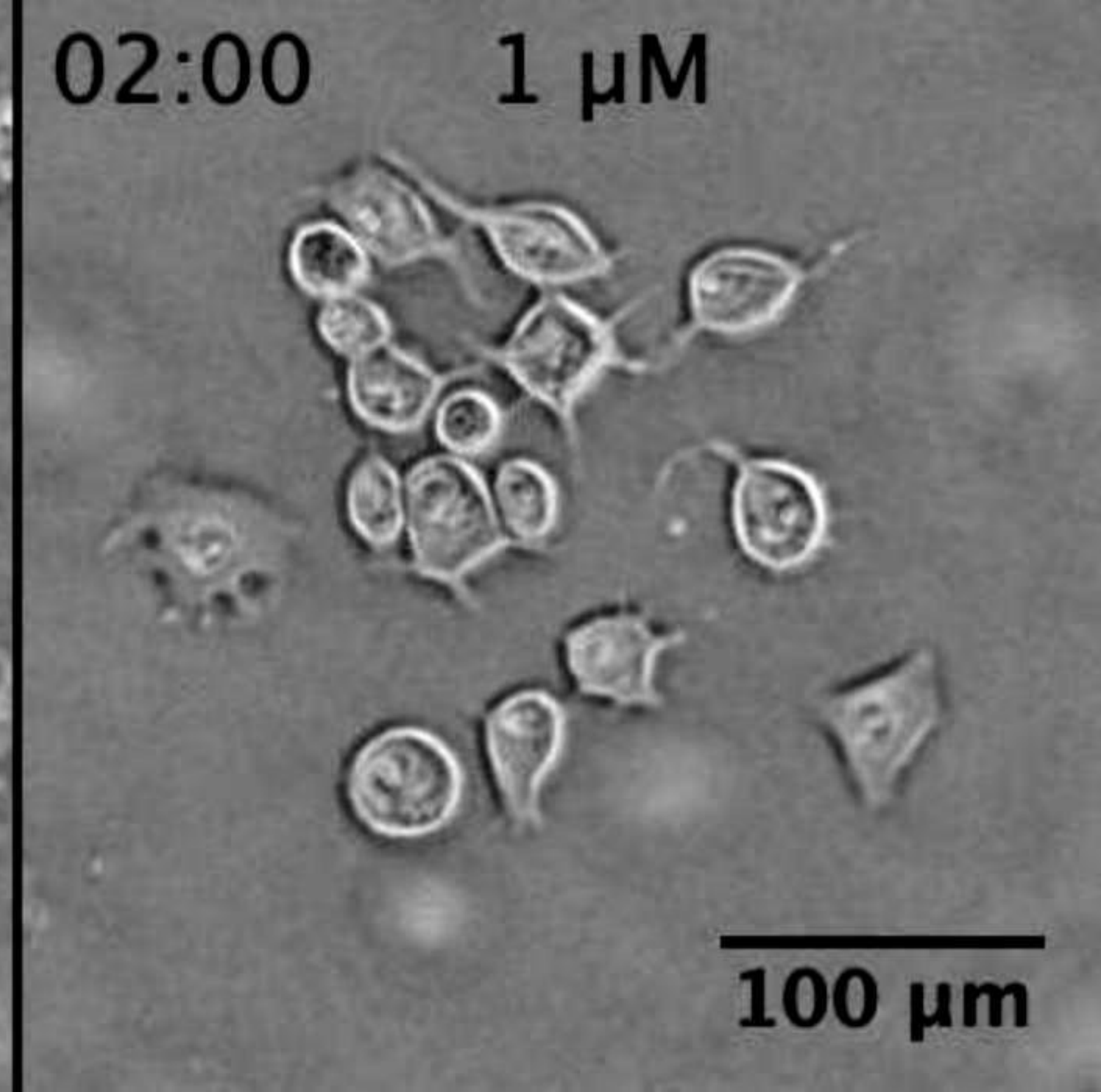

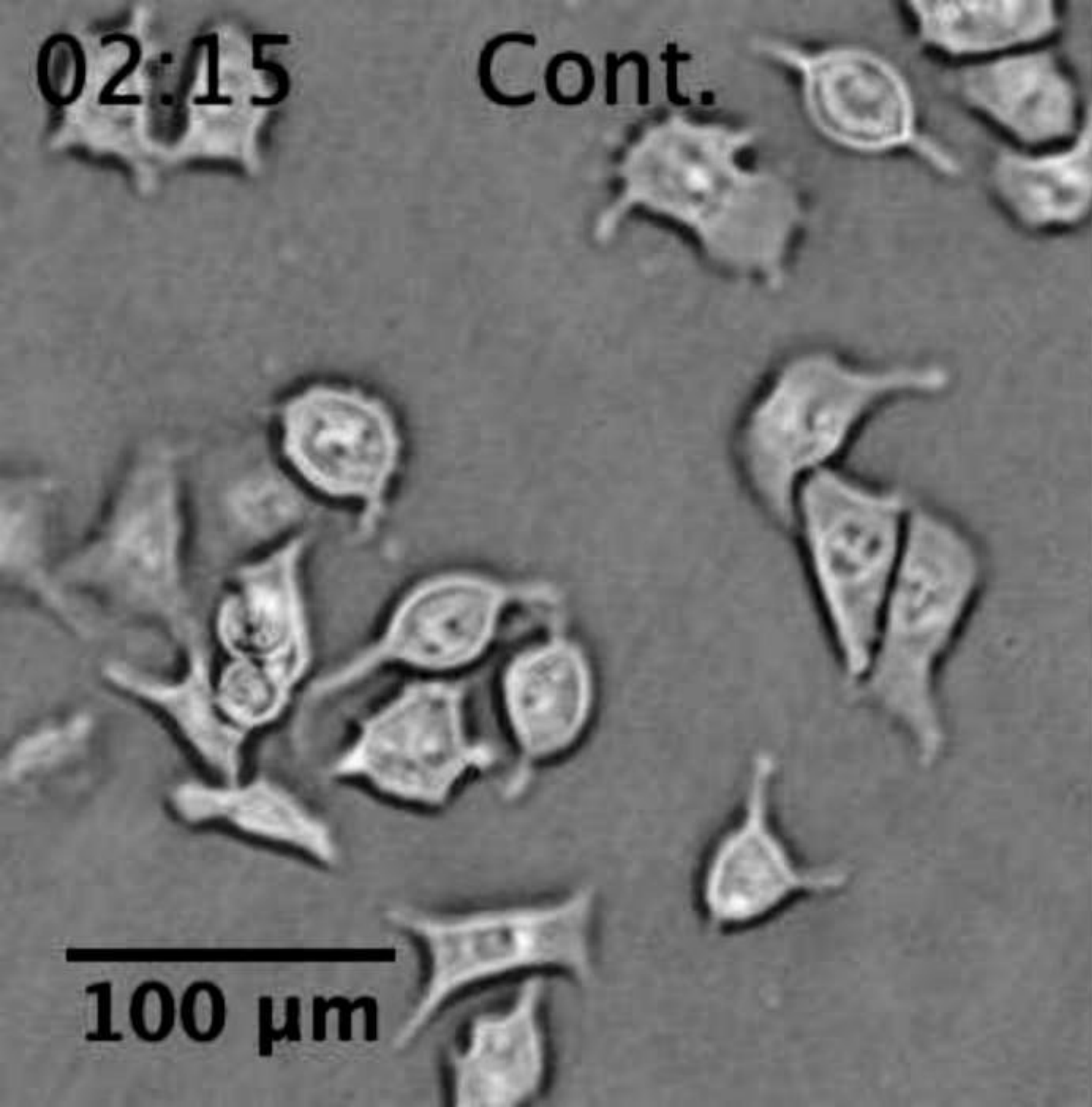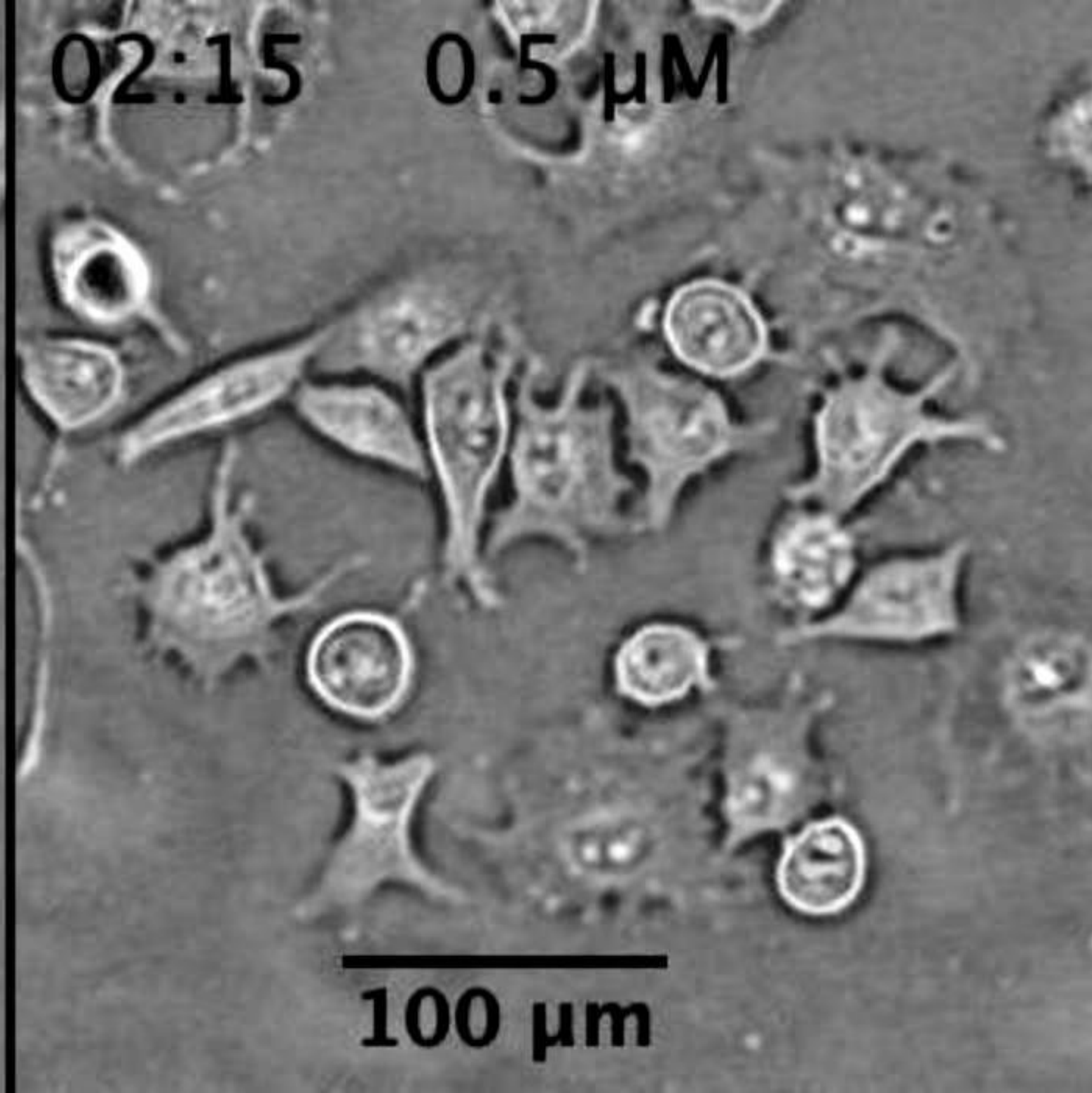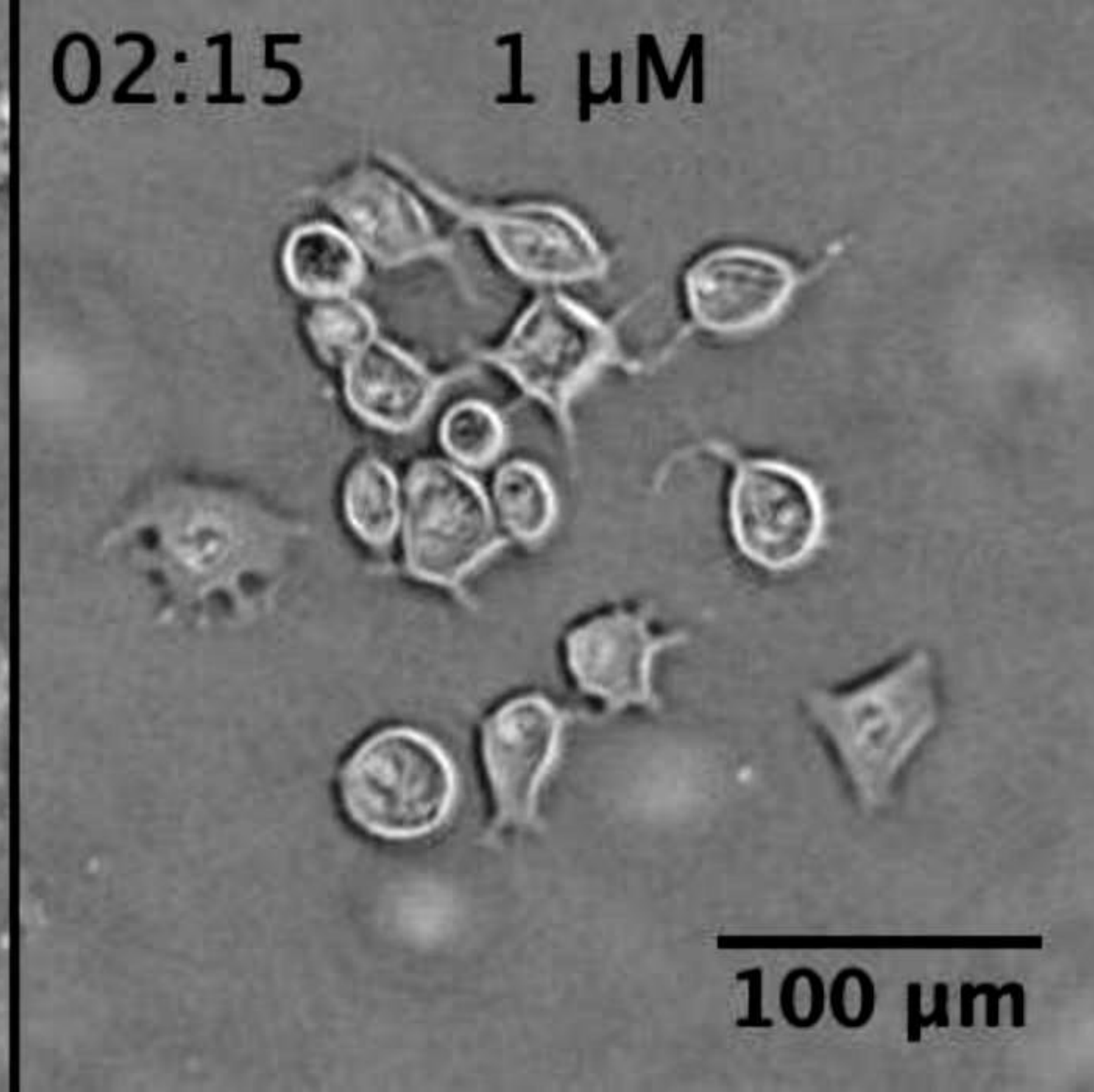

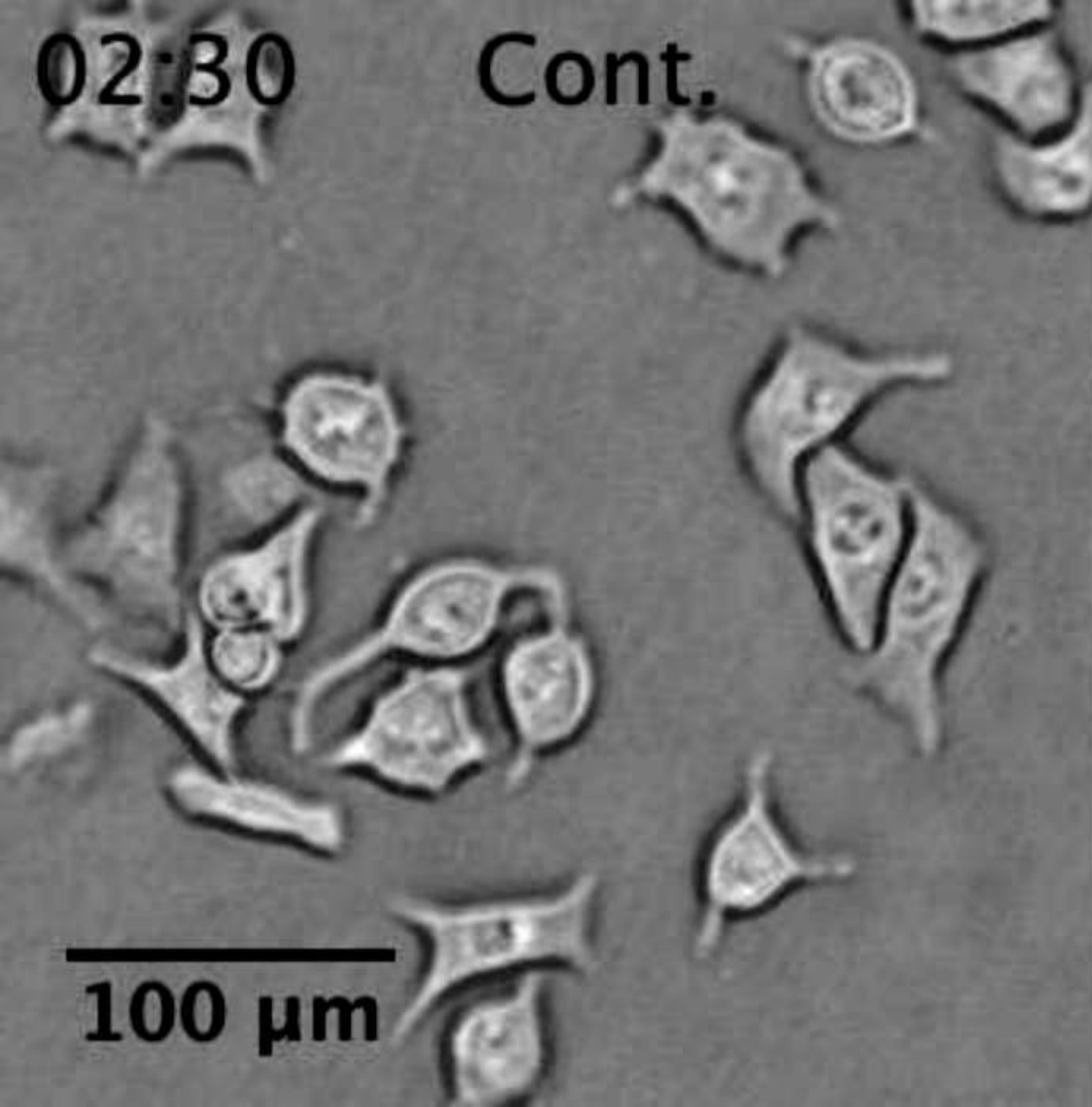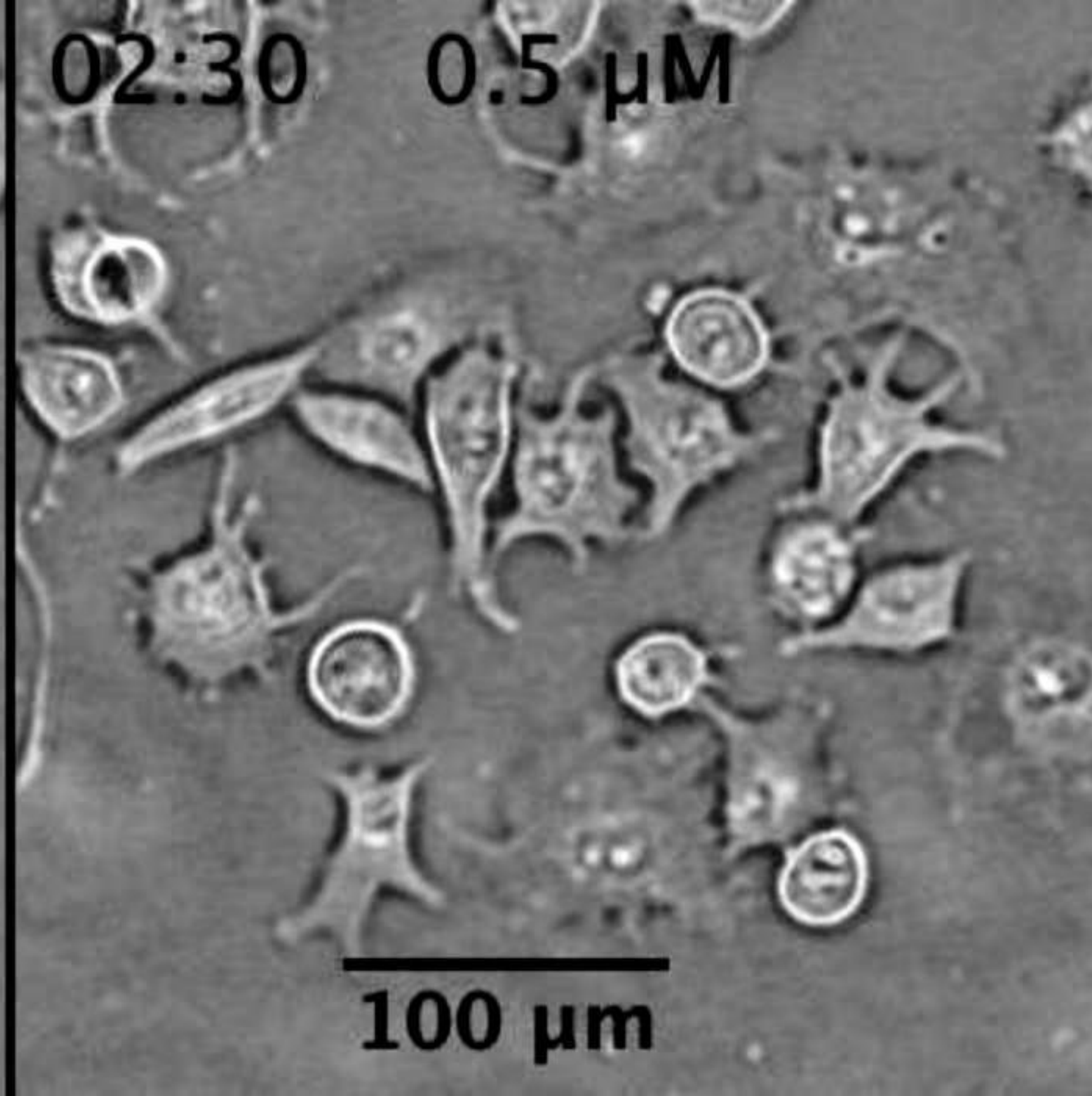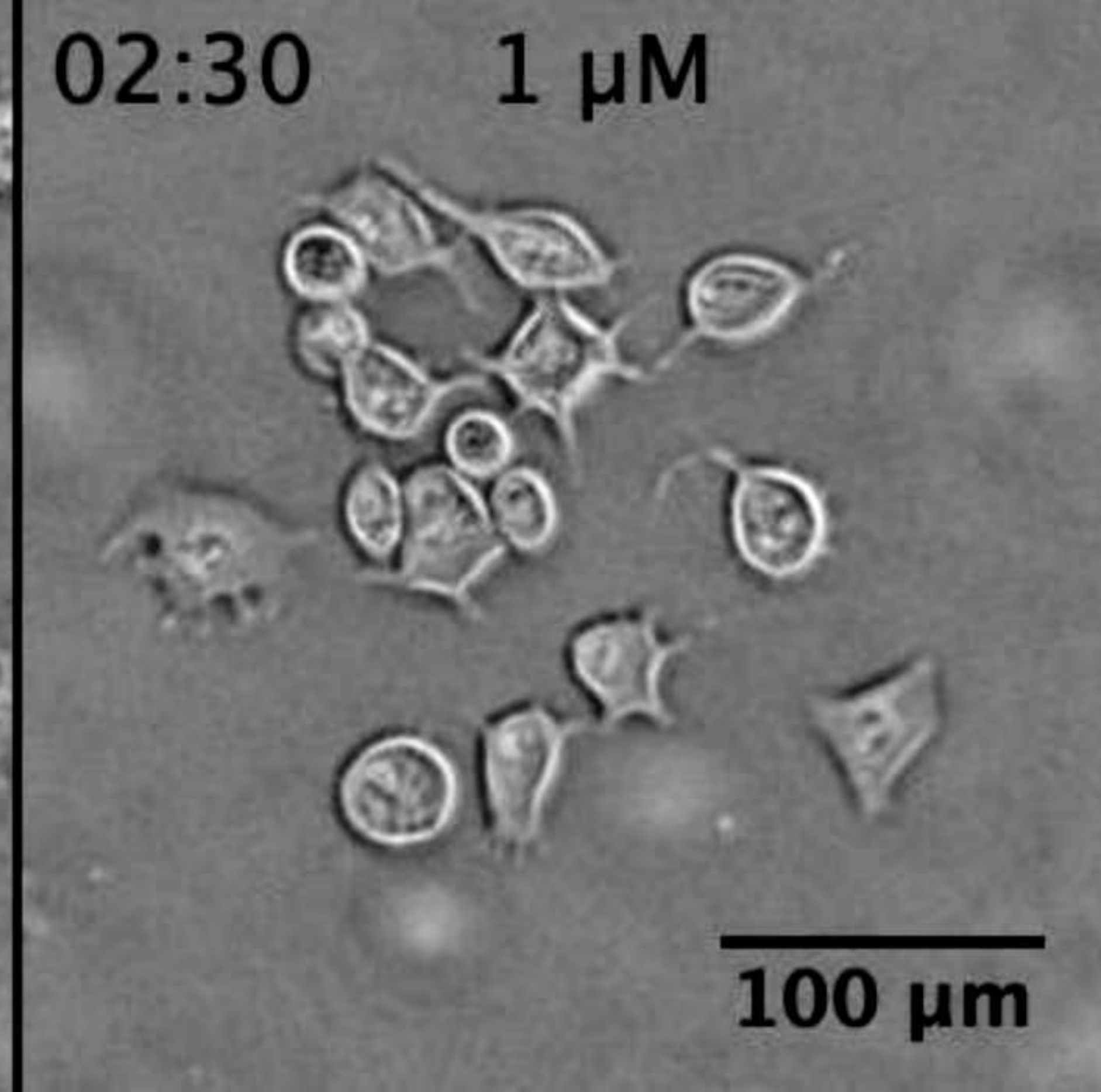

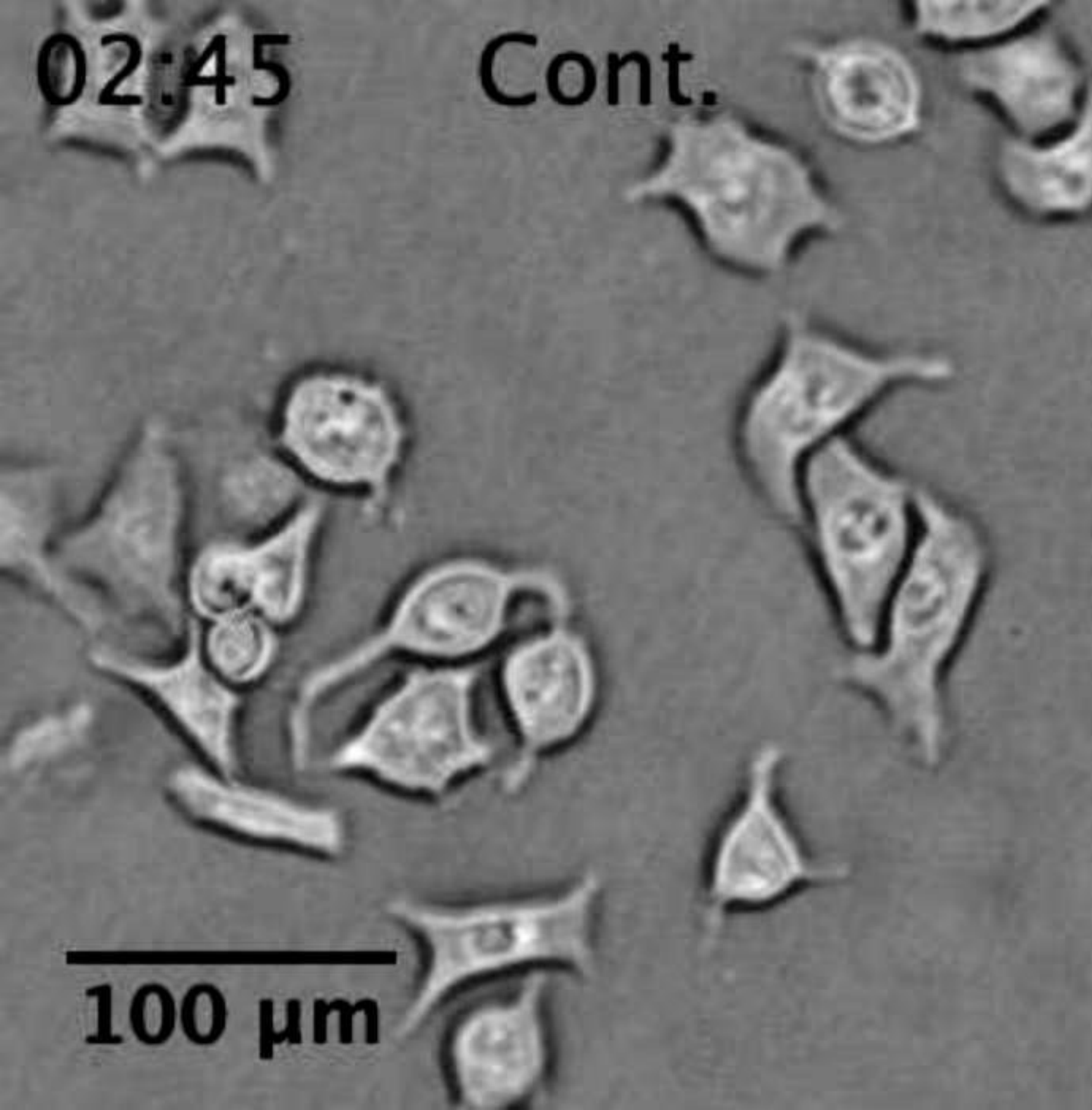

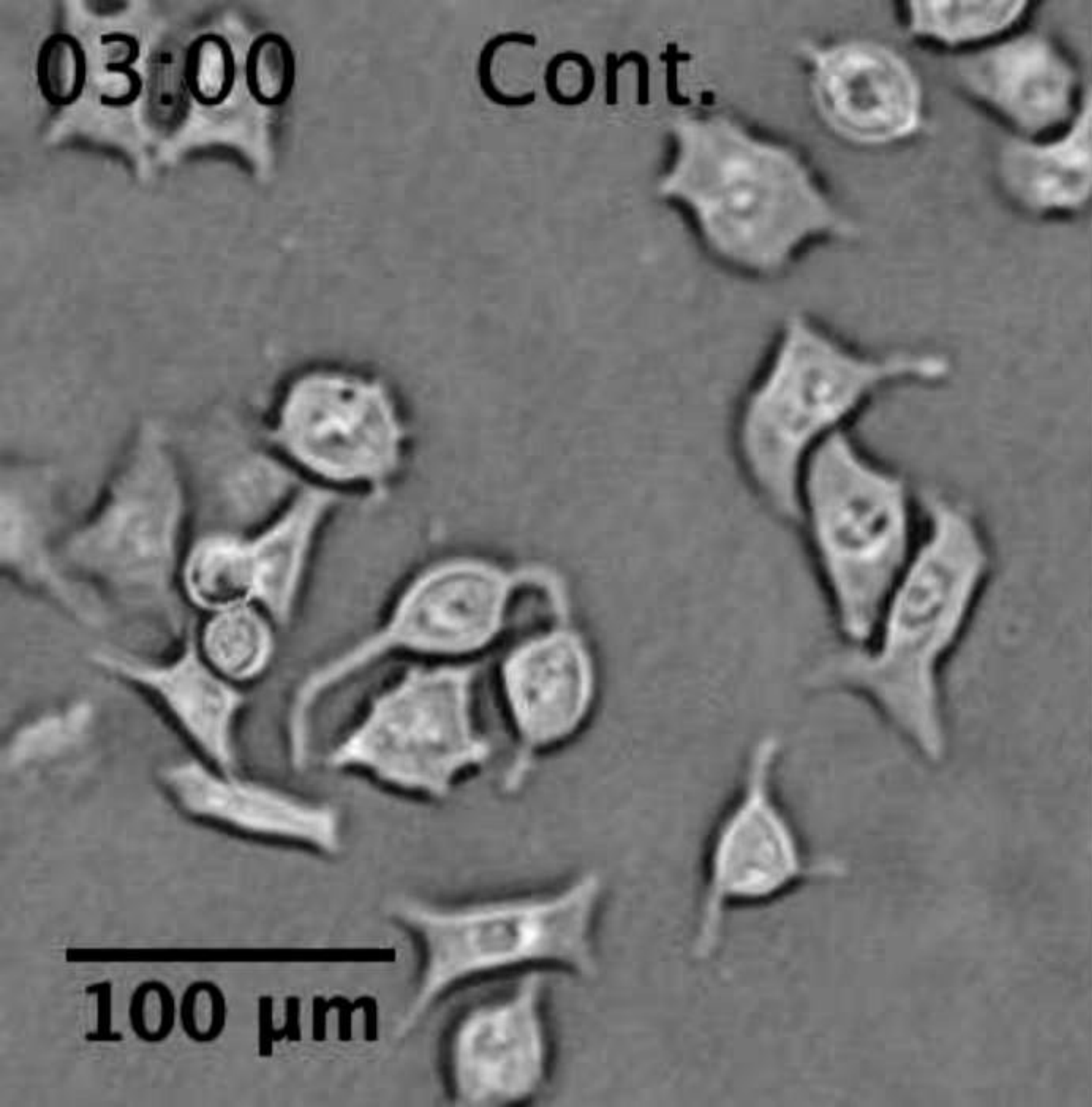

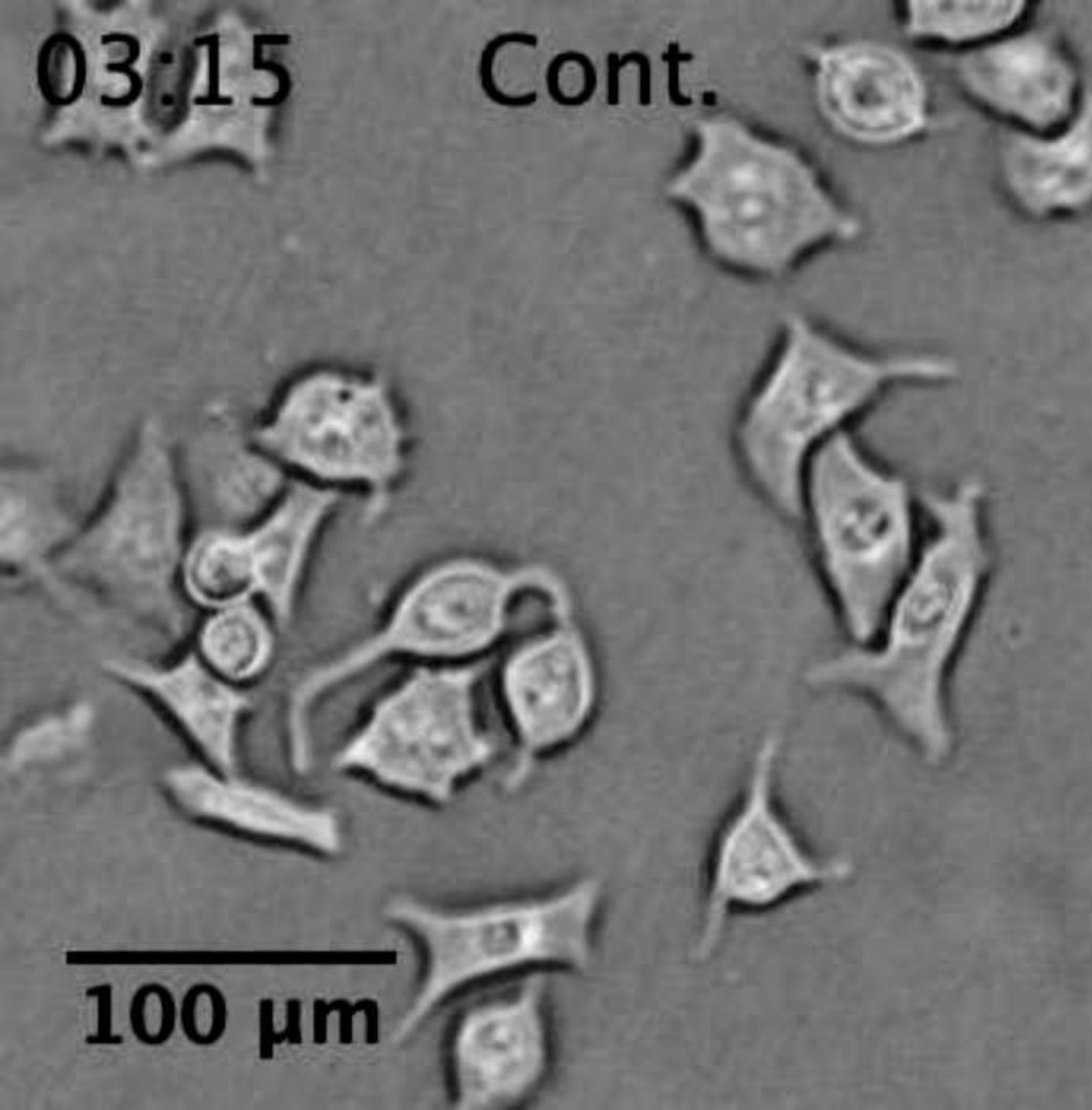

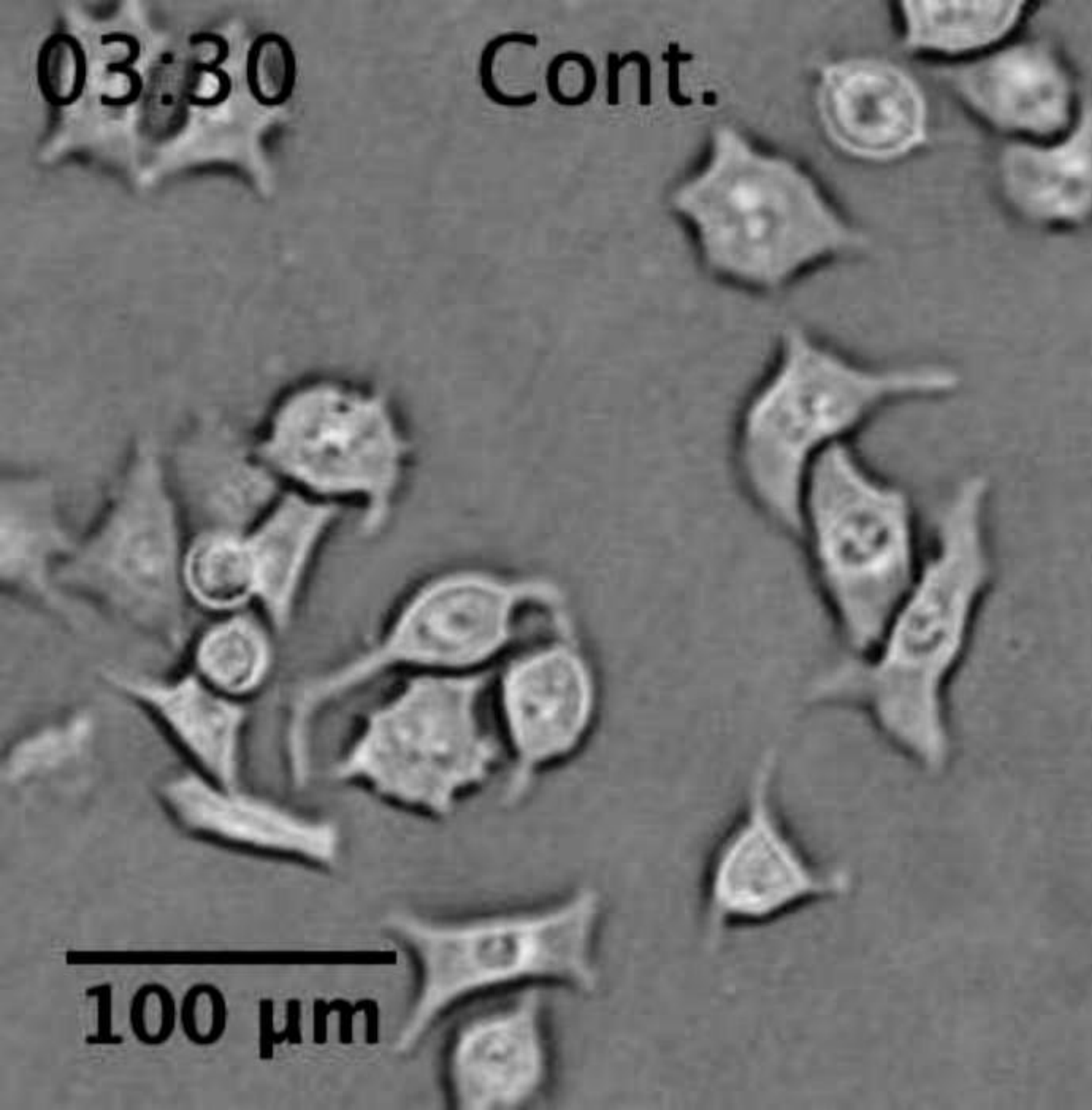

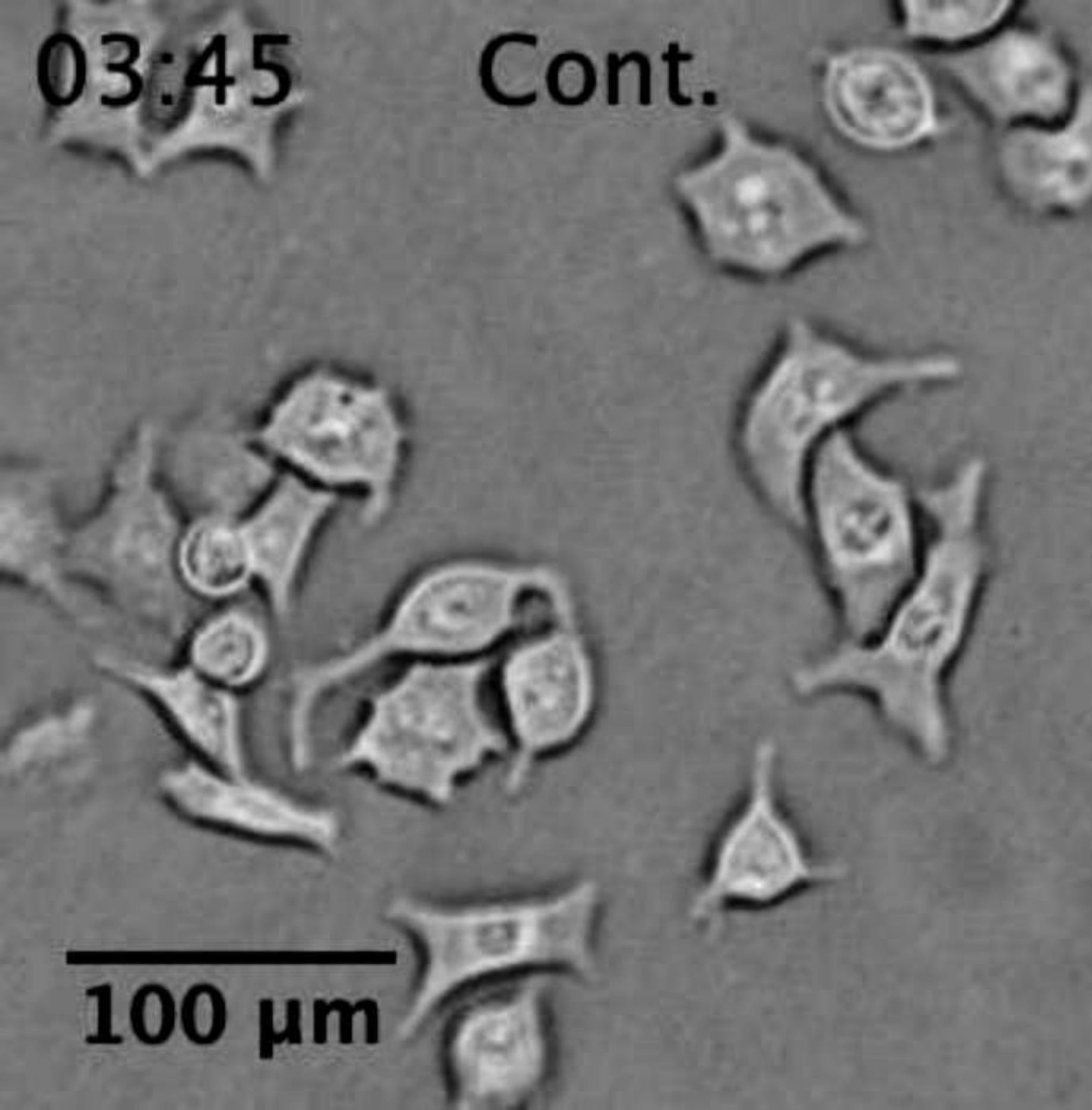

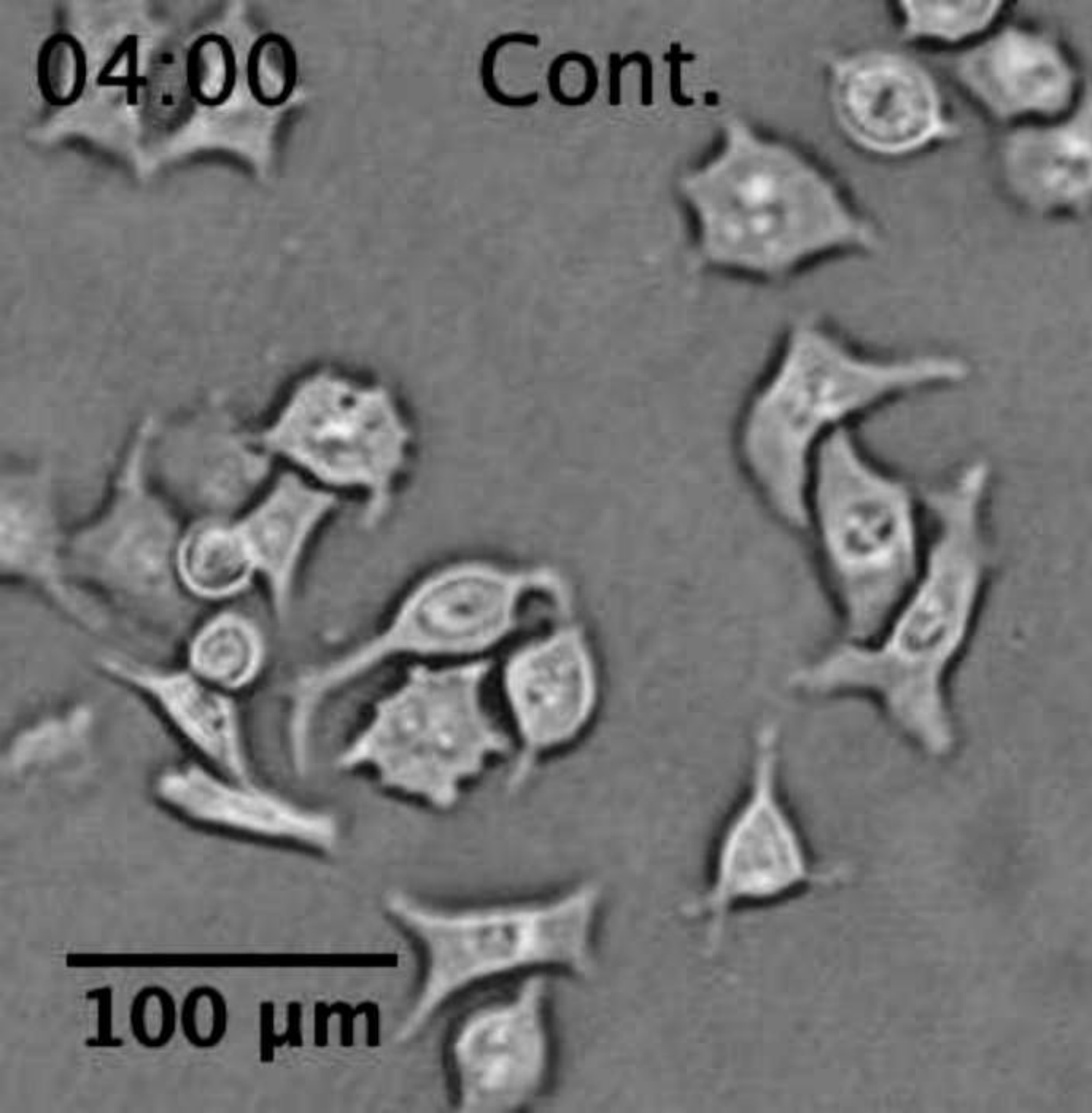

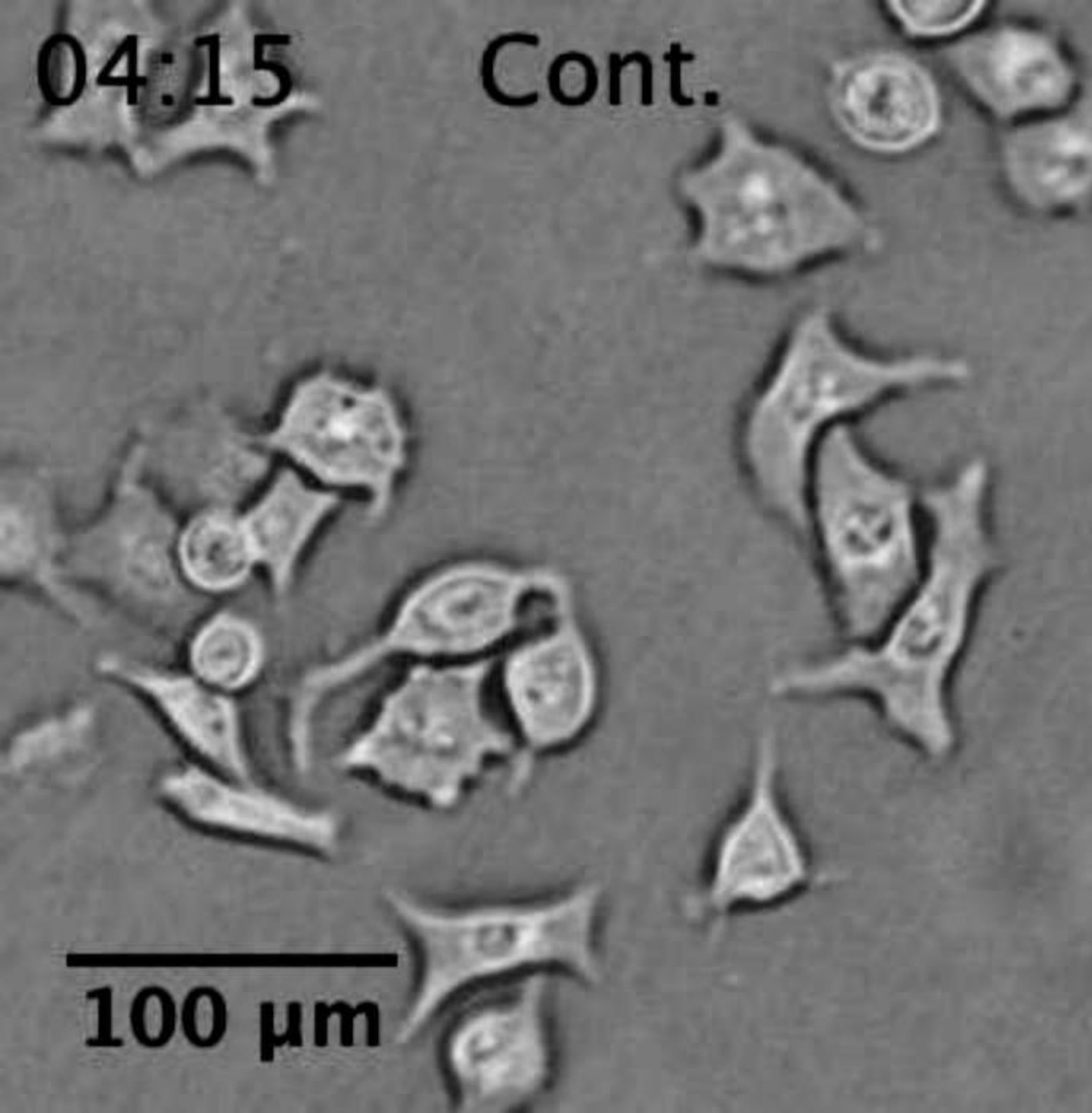

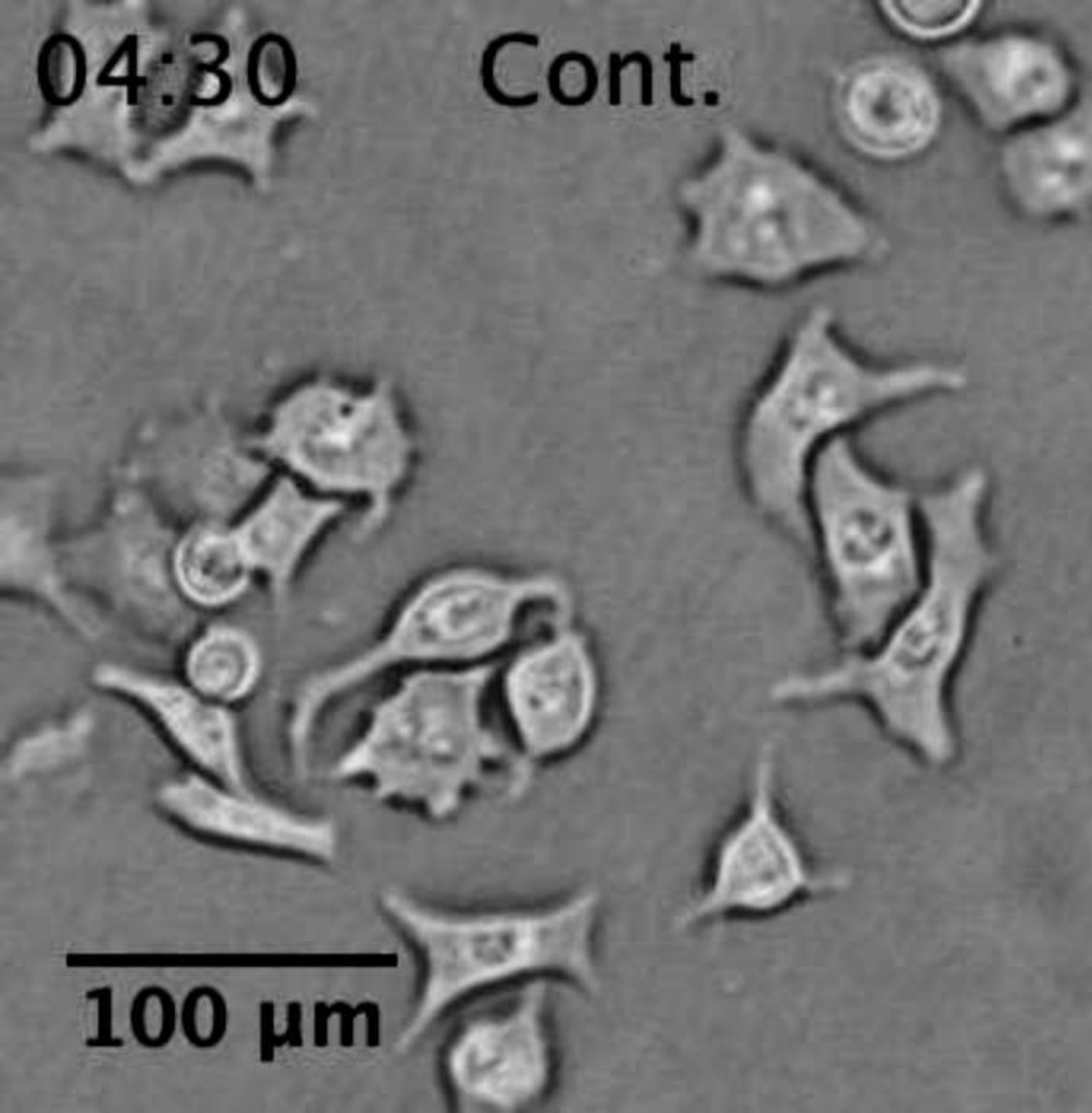

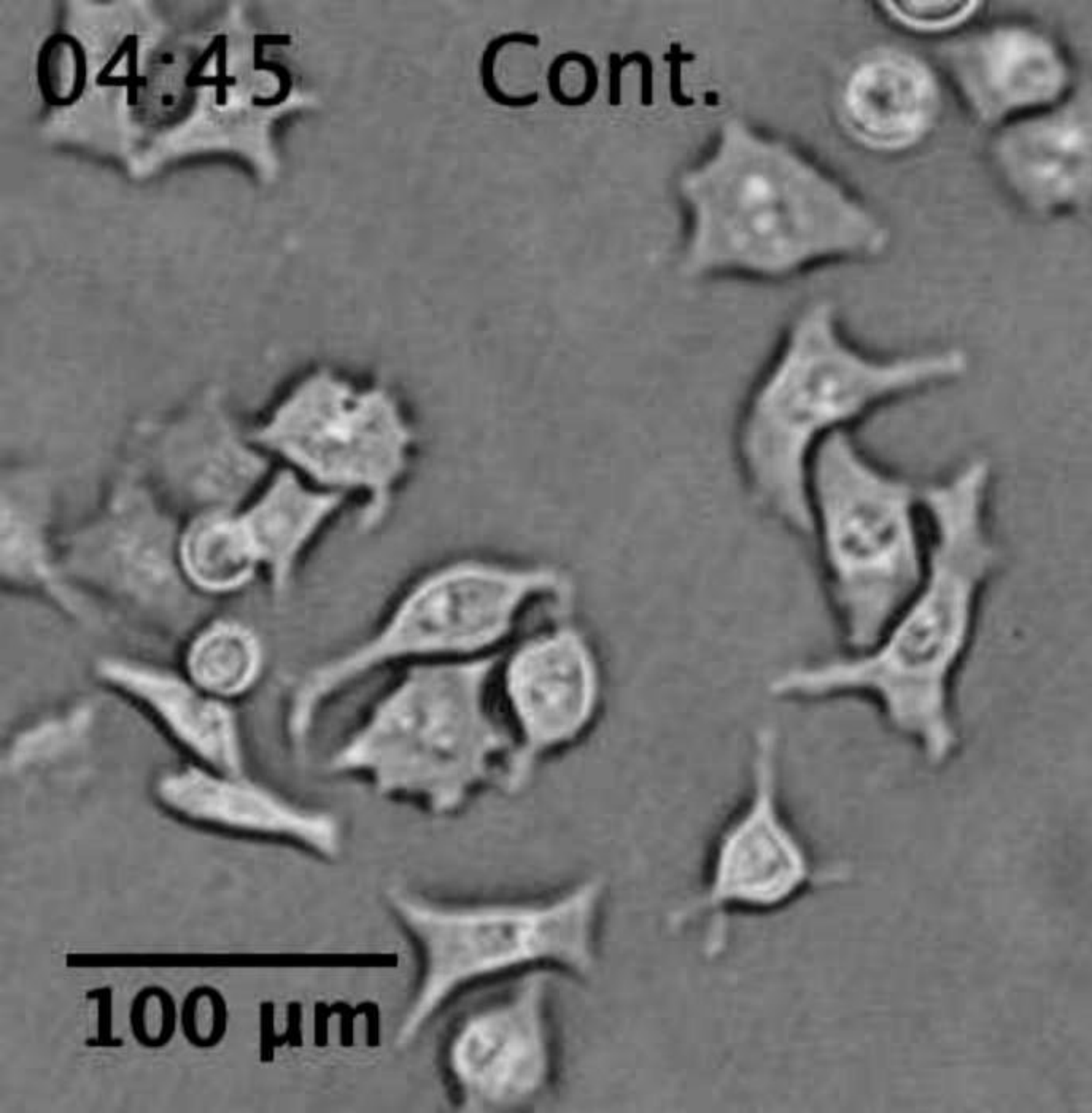

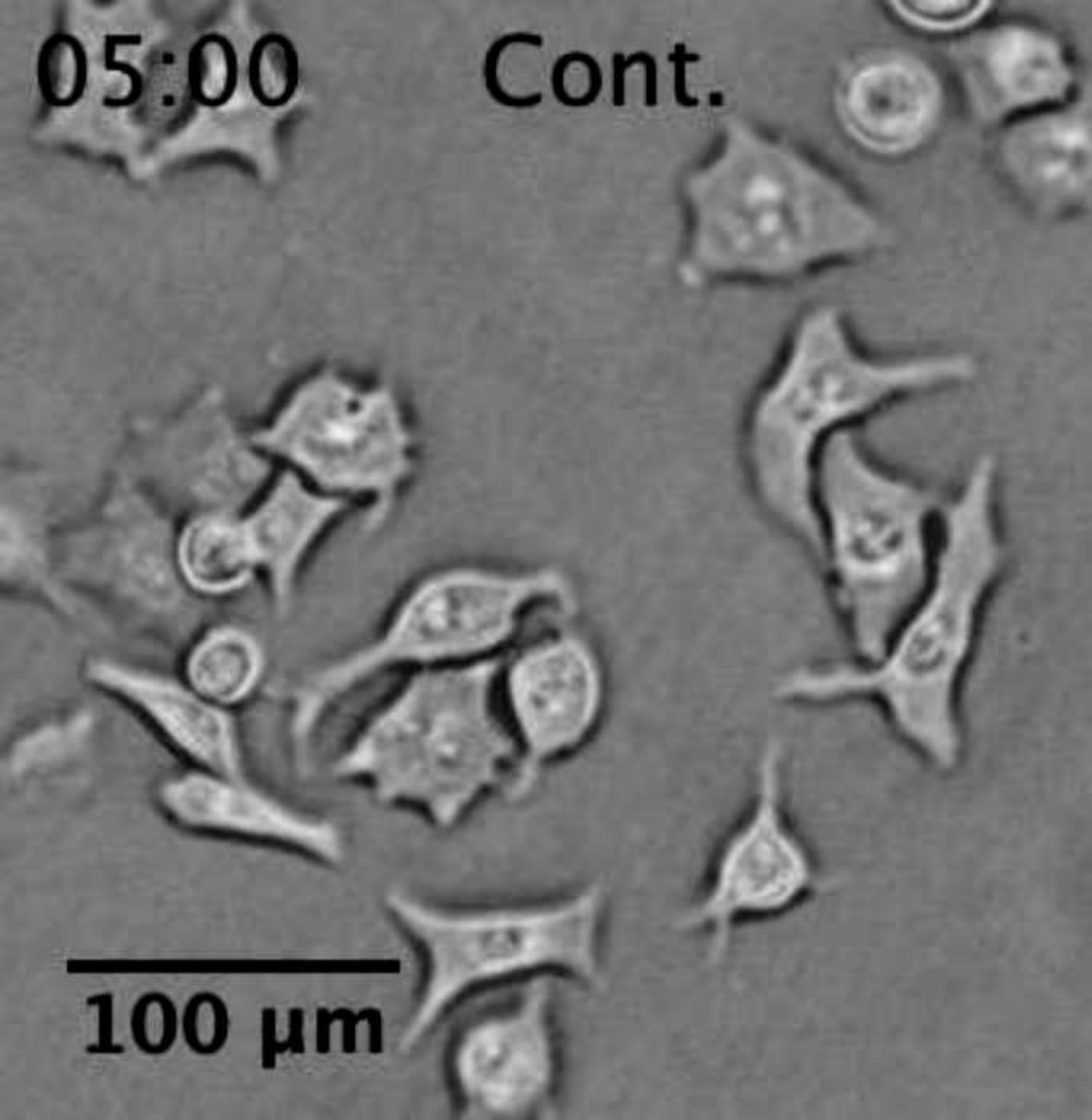

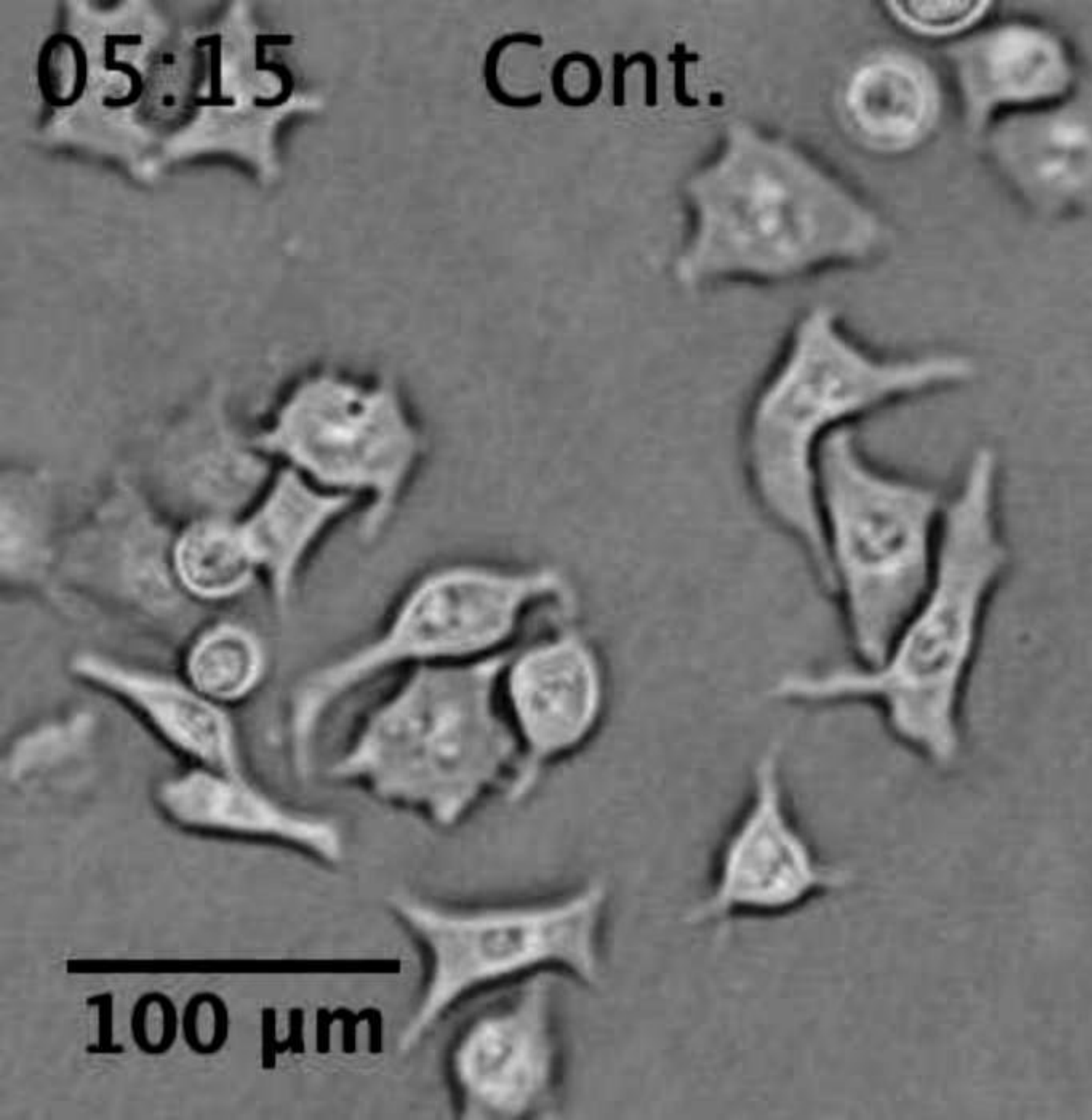

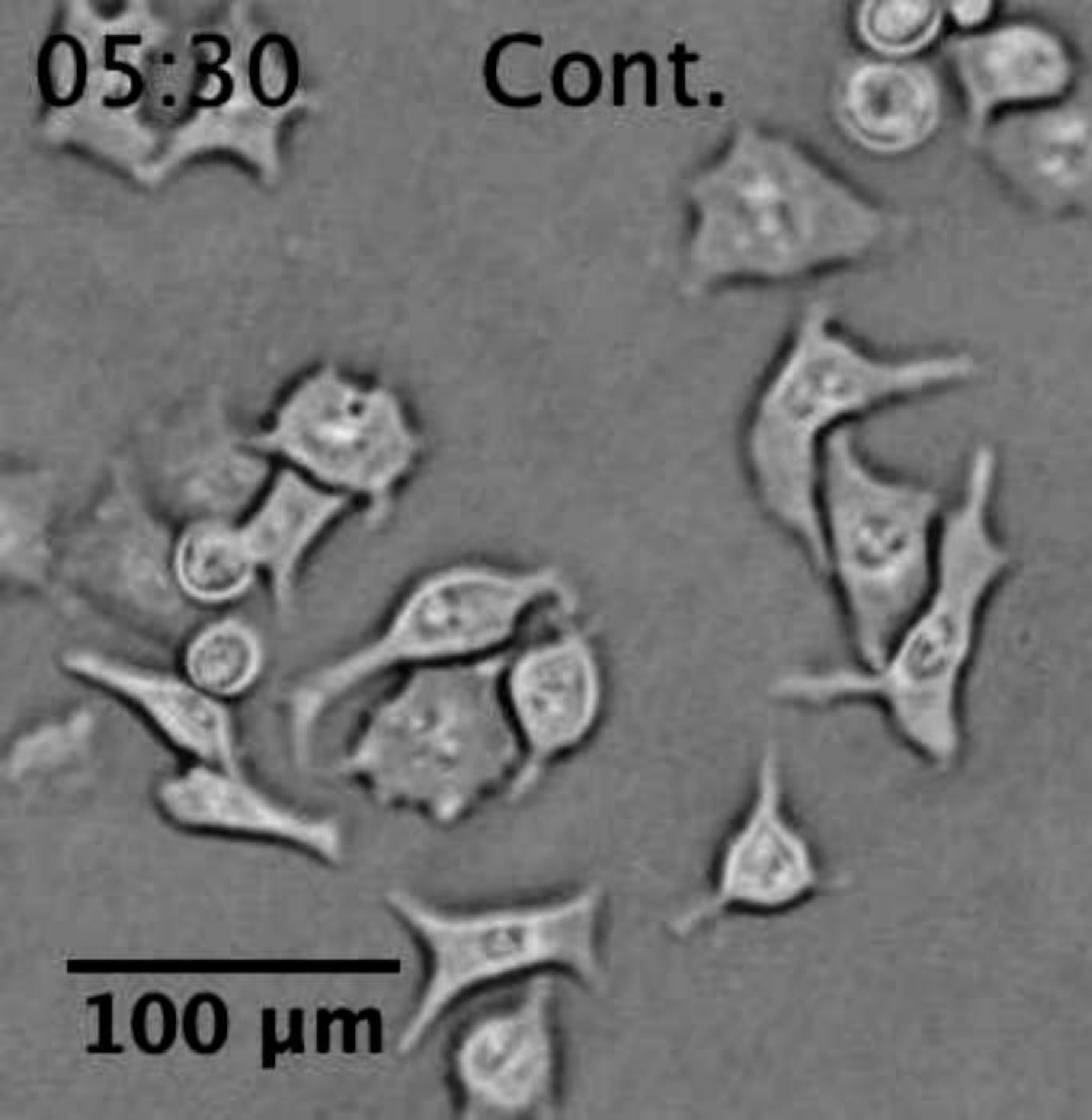

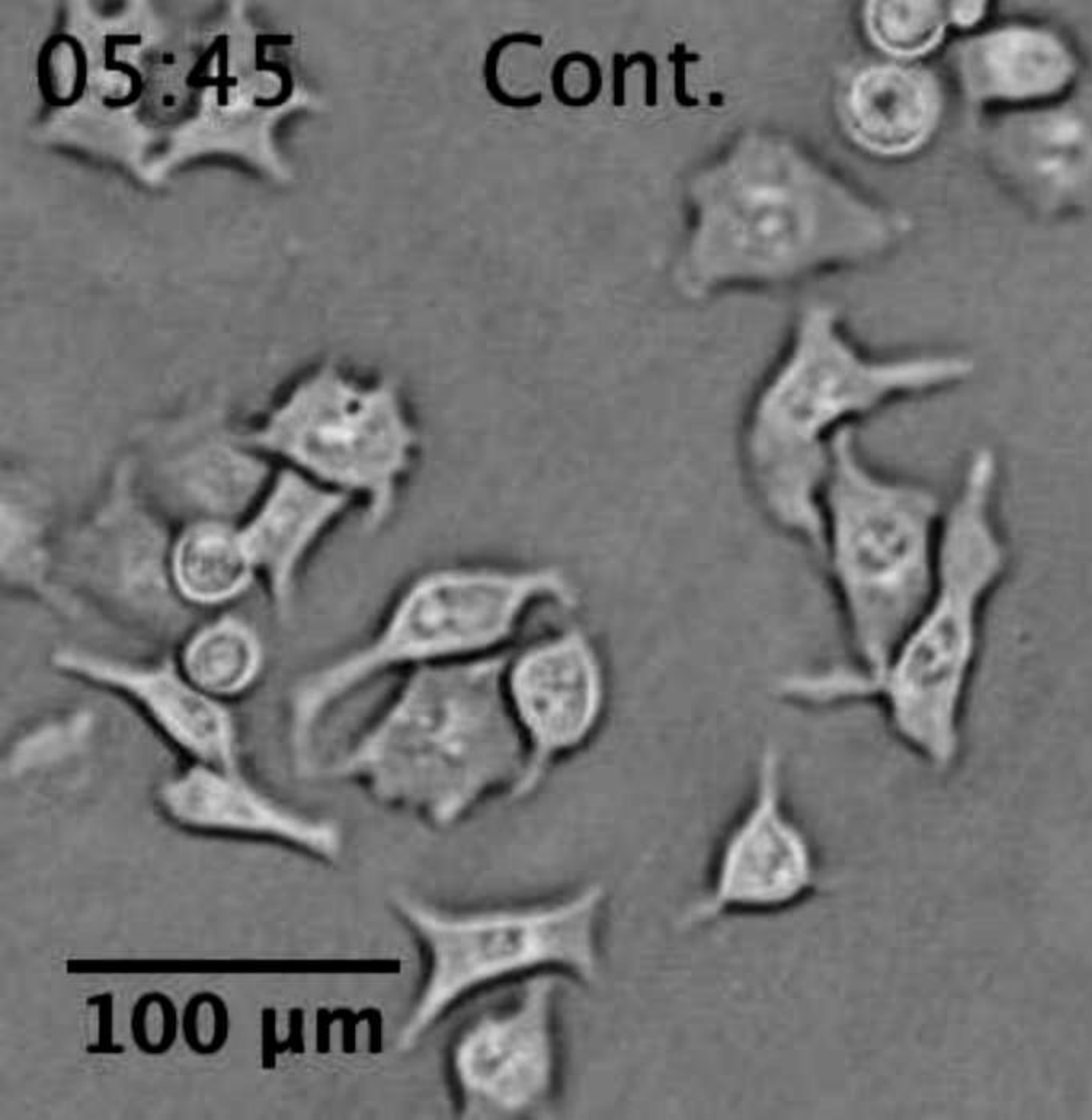

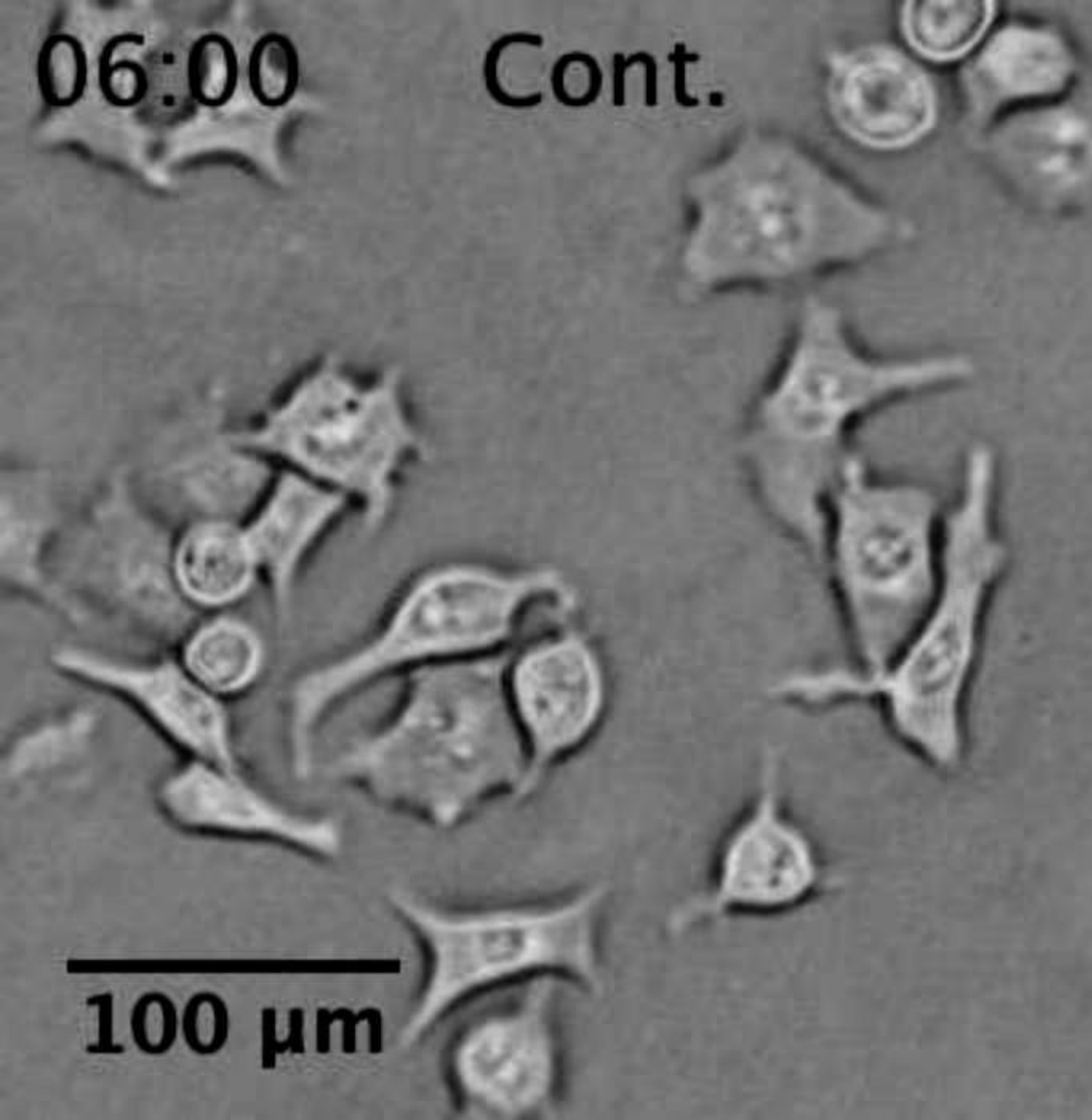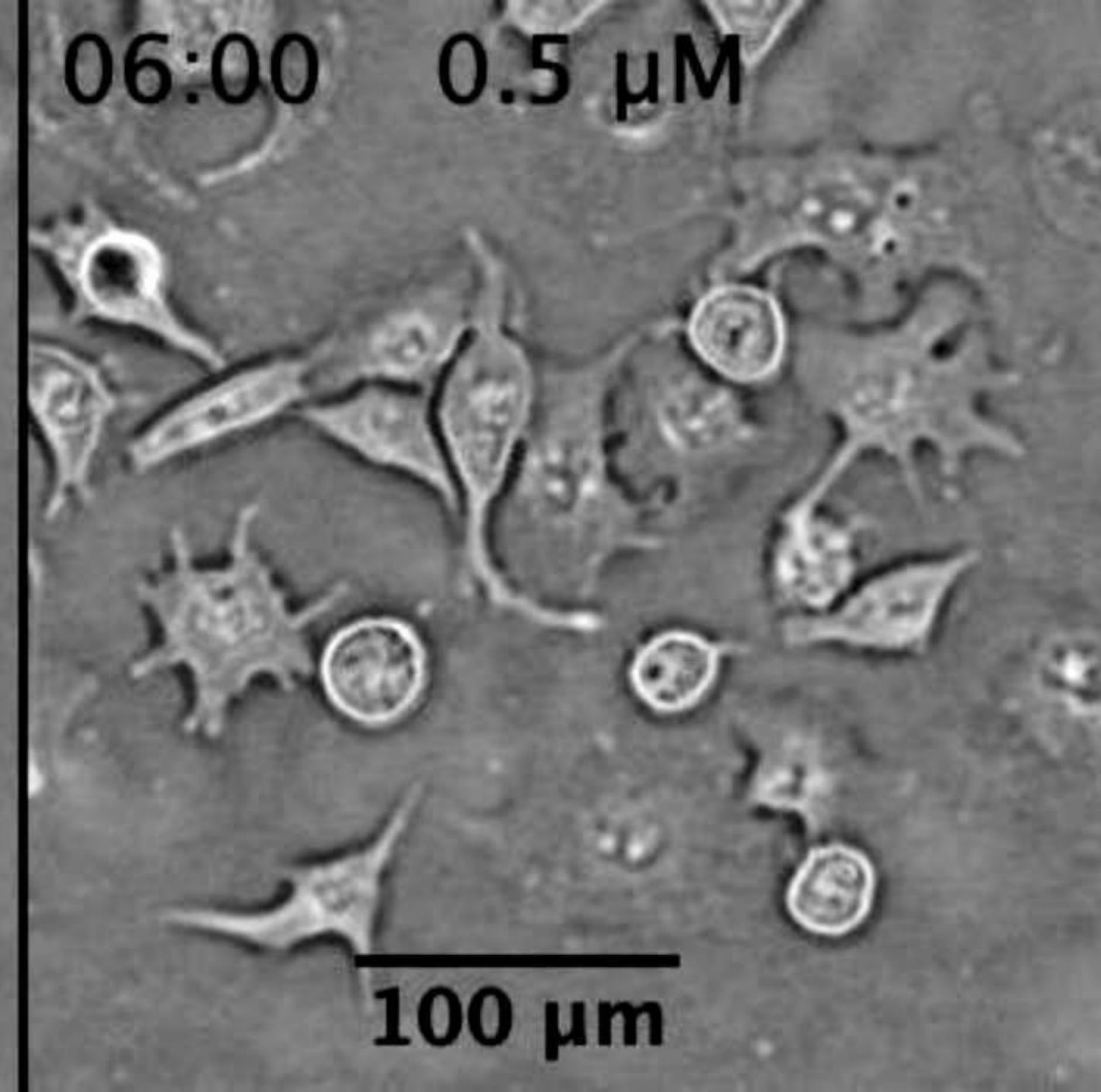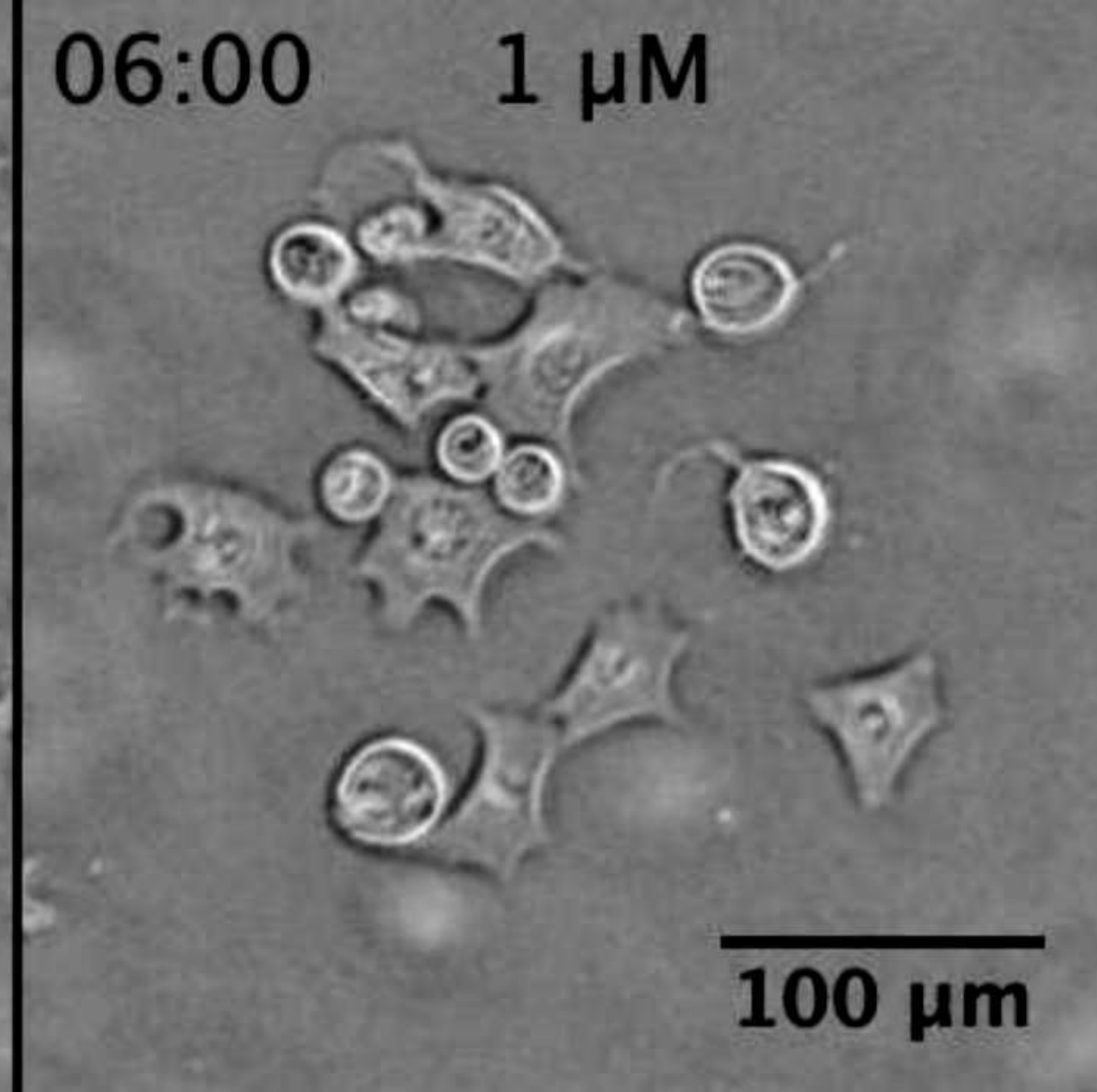

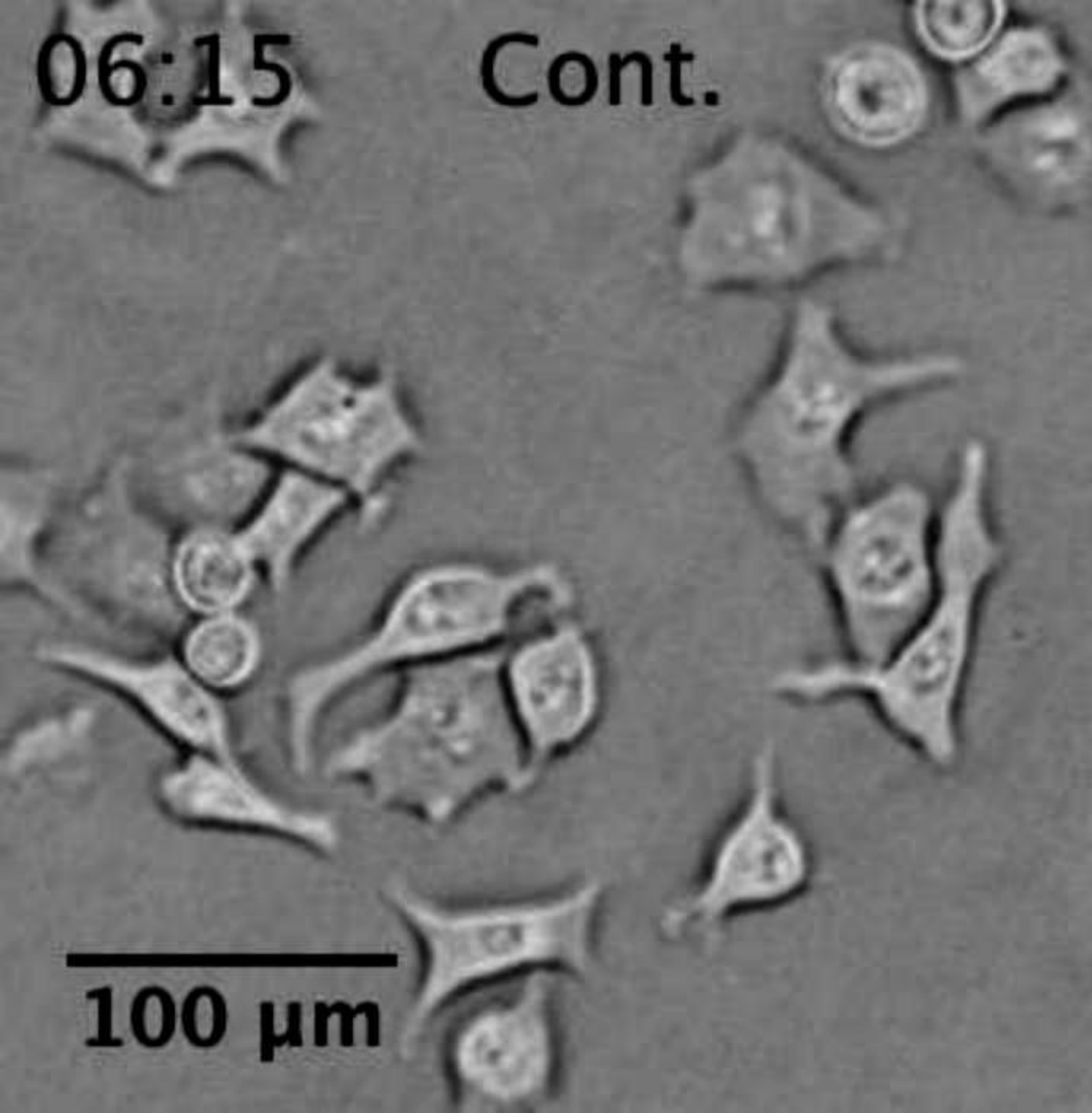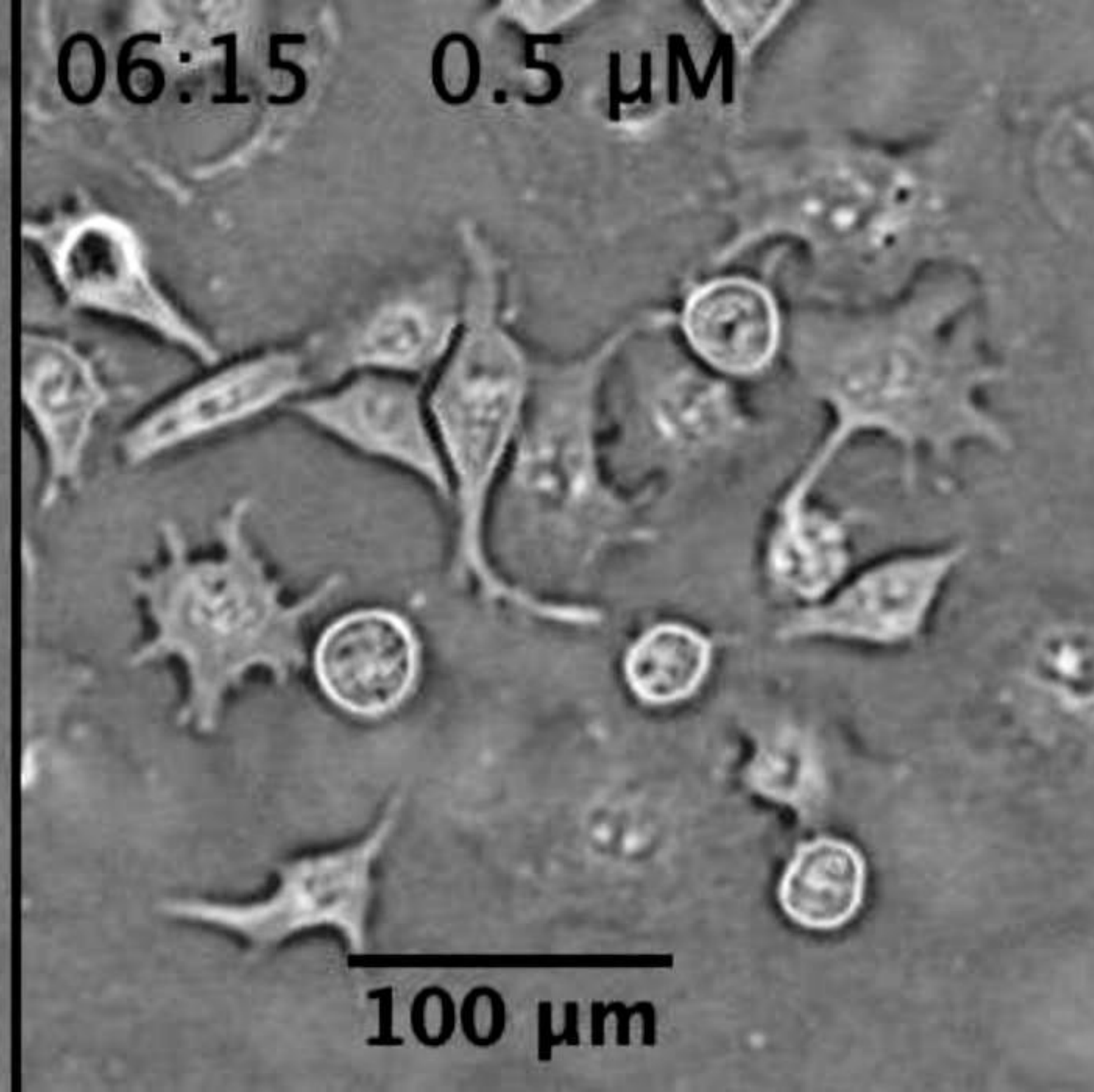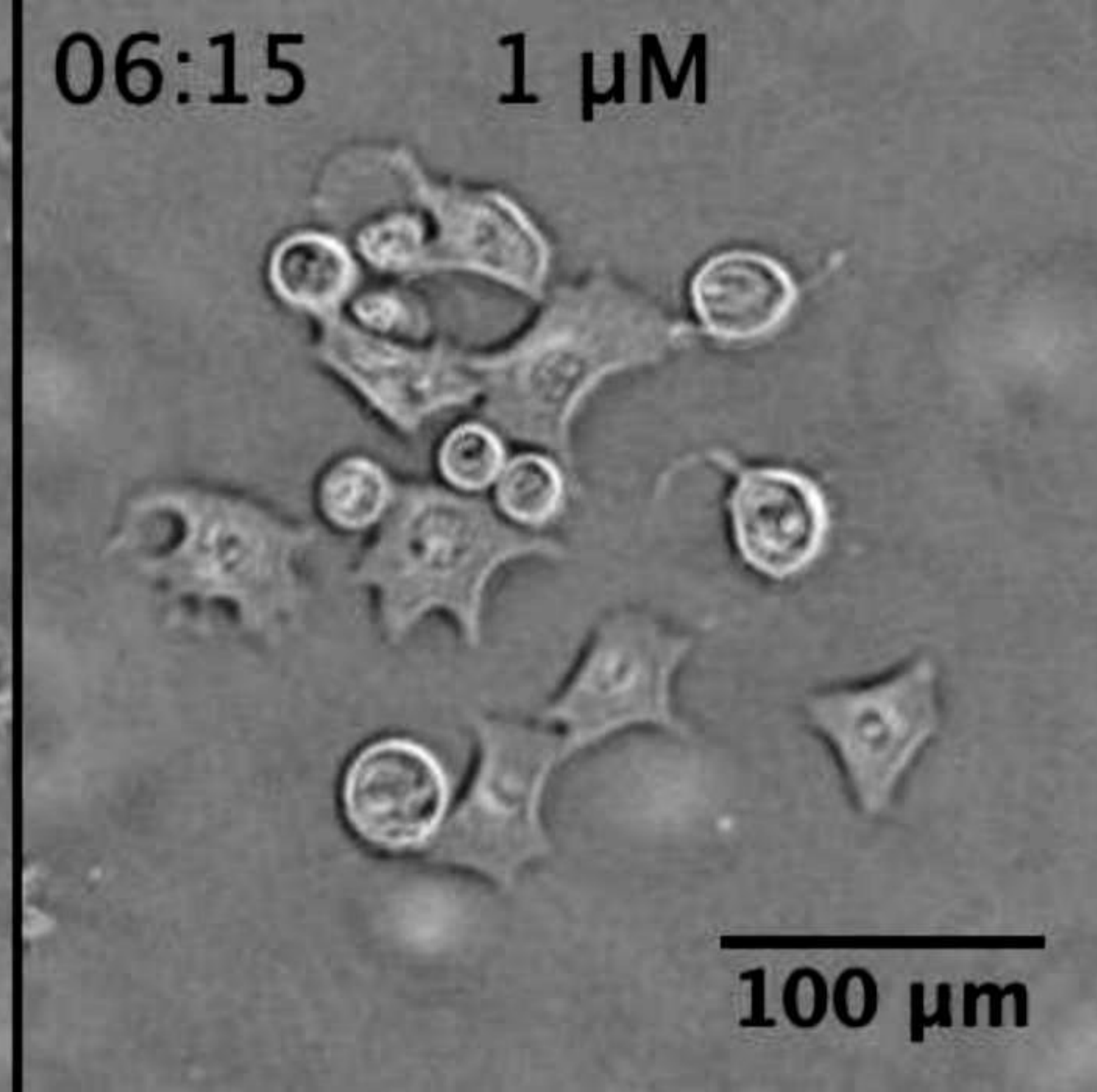

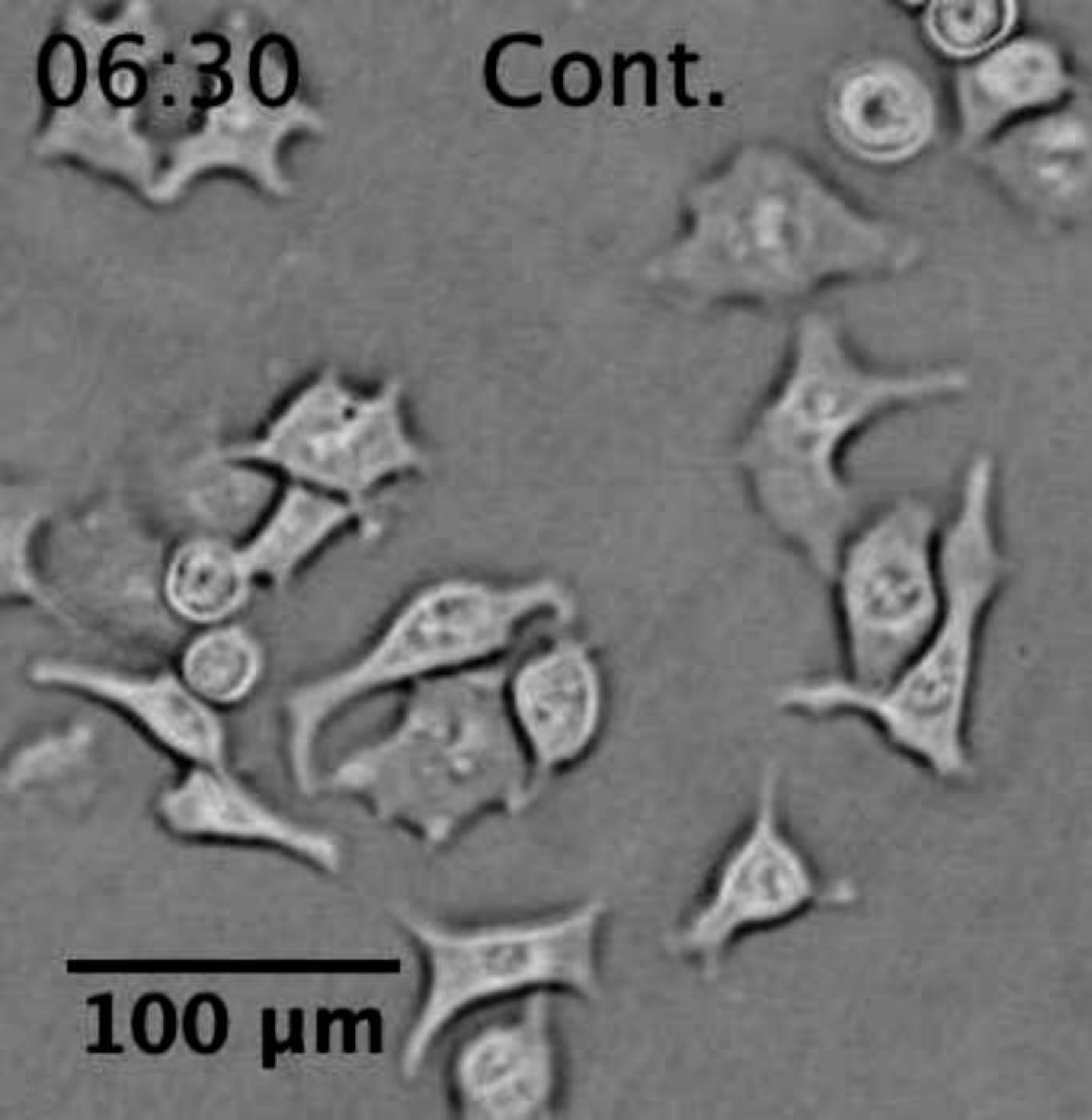

Supplement: Supplementary file 1 [file plants-11-02466-s001.zip › Supporting information-II.pdf]
